# Chemoenzymatic Triazolopyridine Synthesis Enabled by Cryptic Diazo Formation by Vanadium-Dependent Haloperoxidases

**DOI:** 10.1021/acs.orglett.6c00937

**Published:** 2026-04-13

**Authors:** Manik Sharma, Kyle F. Biegasiewicz

**Affiliations:** † Department of Chemistry, 1371 Emory University, Atlanta, Georgia 30322, United States

## Abstract

Triazolopyridines are an important class of heterocycles in the pharmaceutical industry and materials sciences. In particular, [1,2,3]­triazolo­[1,5a]­pyridines have emerged as stable and versatile diazo compound precursors for performing carbene-mediated transformations. Despite their wide range of applications in chemical synthesis, their preparation is often reliant on oxidative cyclization methods using stoichiometric oxidants in an organic solvent, limiting their application in chemoenzymatic synthesis. We have recently discovered that vanadium-dependent haloperoxidase (VHPO) enzymes are effective catalysts for performing the oxidative cyclization of 2-pyridyl ketone hydrazones to give [1,2,3]­triazolo­[1,5a]­pyridines through cryptic diazo formation. Herein, we have developed a chemoenzymatic protocol for conversion of 2-pyridyl ketones to [1,2,3]­triazolo­[1,5a]­pyridines in a single vessel through the *in situ* generation of 2-pyridyl ketone hydrazones followed by VHPO-catalyzed oxidative cyclization to give [1,2,3]­triazolo­[1,5a]­pyridines in high yield and chemoselectivity.

## Graphical abstract

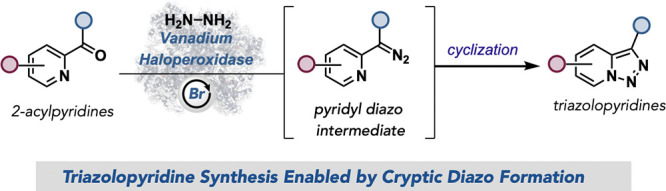

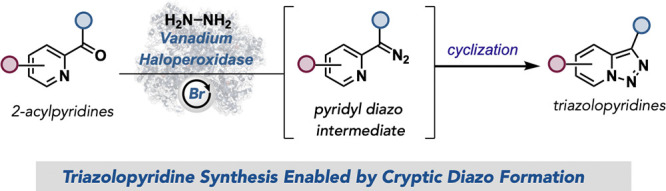

Triazolopyridines are an important class of heterocyclic compounds in medicinal chemistry and the materials industry.^1–3^ Among these, [1,2,3]­triazolo­[1,5a]­pyridines have emerged as antiparasitic agents for Chagas disease treatment,^4,5^ and stable carbene precursors for accessing pyridyl diazo intermediates through ring–chain isomerization in chemocatalysis,^1,6^ and biocatalysis (Figure 1a).^7^ The primary strategy for accessing [1,2,3]­triazolo­[1,5a]­pyridines is through the oxidative cyclization of 2-pyridyl ketone hydrazones. Despite the successful application of this reaction type, established methods rely on the stoichiometric use of oxidizing agents and organic solvents that limit their application in chemoenzymatic synthesis,^8–20^ leaving an efficient, selective, and biocompatible alternative for [1,2,3]­triazolo­[1,5a]­pyridines highly desired (Figure 1b).

**Figure 1:**
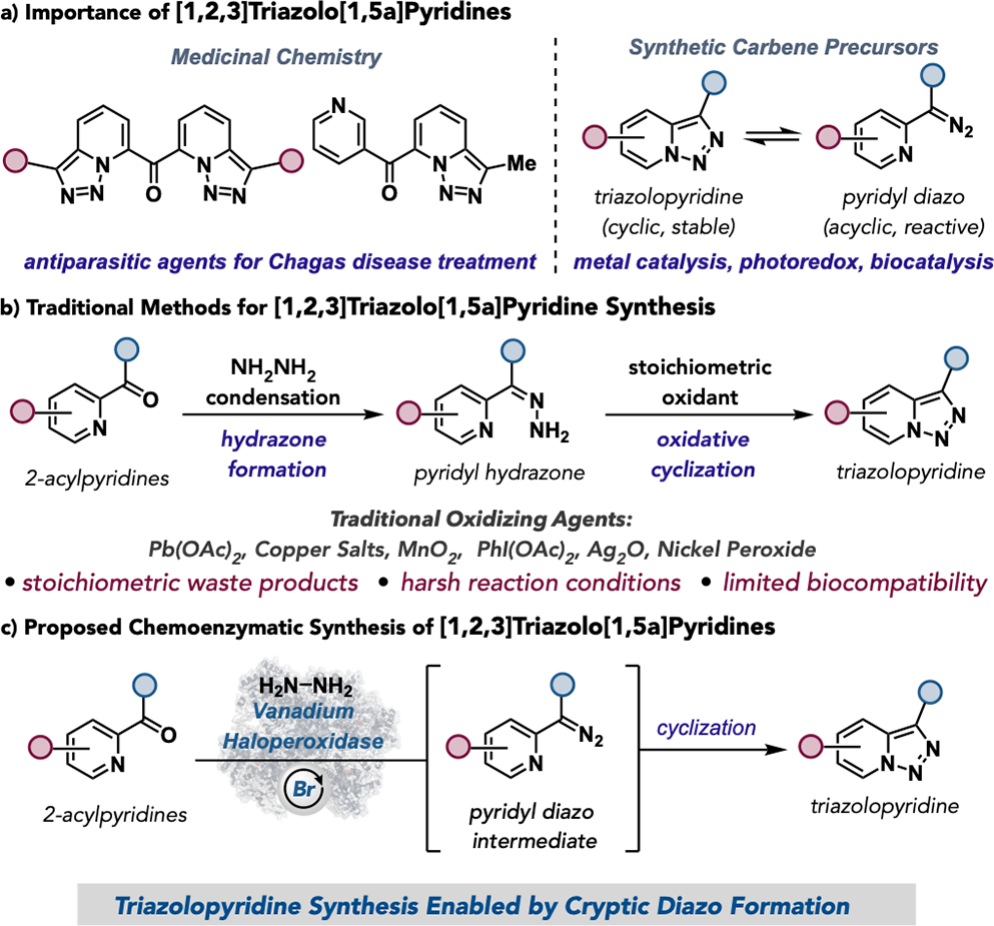
(a) Importance of [1,2,3]­triazolo­[1,5a]­pyridines. (b) Traditional methods for [1,2,3]­triazolo­[1,5a]­pyridine synthesis. (c) Proposed chemoenzymatic synthesis of [1,2,3]­triazolo­[1,5a]­pyridines.

Enzymes are an attractive option for cryptic diazo formation because of their inherent selectivity and sustainability parameters.^21–24^ While a host of enzymes have been identified or implicated in diazo formation in the context of natural product biosynthesis,^25–31^ nature is void of enzymes that have used cryptic diazo formation to facilitate nitrogen–nitrogen (N–N) bond formation. With the rapidly emerging interest in the discovery and application of N–N bond-forming enzymes in biocatalysis,^32–34^ the discovery of enzymes capable of facilitating this type of transformation would provide a unique enzymatic strategy for accessing [1,2,3]­triazolo­[1,5a]­pyridines and allow for the application of selective cryptic diazo formation in chemoenzymatic synthesis. One option for cryptic diazo formation would be through enzymatic halogenation. Among the wide range of halogenating enzymes in nature,^35–38^ the vanadium-dependent haloperoxidase (VHPO) class of enzymes has emerged as a promising biocatalytic platform for chemical synthesis because of their reaction condition tolerance and ability to operate without an exogenous turnover system.^39–42^ In addition to numerous examples using VHPOs for reactions outside of their natural substrate scope,^43–48^ we have recently been interested in the ability of VHPOs to perform cryptic halogenation reactions,^49^ where oxidation and bond formation occurs through an enzymatic halide recycling (EHR) mechanism.^50–53^ As part of these efforts, we discovered that VHPOs are effective catalysts for diazo synthesis across a broad range of substrates,^54^ but this biotechnology has yet to be applied to a cryptic halogenation process whereby generation of the diazo intermediate facilitates a new N–N bond formation. Herein, we report that VHPOs are a viable catalyst system for [1,2,3]­triazolo­[1,5a]­pyridine synthesis through cryptic diazo formation (Figure 1c).

We began our investigation of VHPO-mediated oxidative cyclization with conversion of 2-(1-hydrazineylideneethyl)­pyridine (**1**) to 3-methyl-[1,2,3]­triazolo­[1,5-*a*]­pyridine (**2**). The chloroperoxidase from *Curvularia inaequalis* (*Ci*VCPO) was initially interrogated based on its established synthetic applicability in a wide range of synthetic transformations.^43–45,48,50–56^ Subjection of **1** to *Ci*VCPO (0.0125 mol %), sodium orthovanadate (Na_3_VO_4_, 1.0 mM), potassium bromide (KBr, 1.0 equiv), and H_2_O_2_ (1.0 equiv) in 1,4-piperazinediethanesulfonic acid (PIPES) buffer (100 mM, pH 6.5) and acetonitrile (MeCN) as a cosolvent (30% v/v) provided **2** in 69% yield in 4 h (Figure 2, Entry 1). To identify a more suitable biocatalyst, a collection of structurally diverse bromoperoxidases from *Corallina officinalis* (*Co*VBPO),^57^ *Corallina pilulifera* (*Cp*VBPO),^58^ and *Acaryochloris marina* (*Am*VBPO)^41^ were explored under the same conditions (Figure 2, Entries 2–4). Among all biocatalysts tested, *Co*VBPO emerged as the superior biocatalyst to produce **2** in 84% yield. Gratifyingly, the reaction can be conducted under EHR conditions using *Co*VBPO by reducing the KBr loading to 0.3 equiv and increasing the H_2_O_2_ loading to 3.0 equiv, providing **2** in 94% yield (Figure 2, Entry 5). Control experiments were performed removing enzyme (*Co*VBPO), Na_3_VO_4_, KBr, and H_2_O_2_ in turn, confirming the necessity of all reaction components (Figure 2, Entries 6–9). A KBr loading range between 0.3 to 4.0 equiv is tolerated, but with a notable decrease in yield when increased to more than 0.5 equiv (Figure S1). Increasing the H_2_O_2_ loading beyond 4.0 equiv leads to significant diminishment of yield (Figure S2). Importantly, both pH and buffer type play significant roles in reaction performance (Figure S3). The reaction can also be performed in a wide range of cosolvents (Figure S4), with the best results in MeCN (20–50% v/v) (Figure S5). To further simplify the protocol, hydrazone **1** was generated from 2-acetylpyridine and hydrazine hydrate (NH_2_NH_2_·H_2_O), followed directly by biocatalytic oxidative cyclization to afford **2** in 92% yield over two steps (Figure 2, Entry 10).

**Figure 2:**
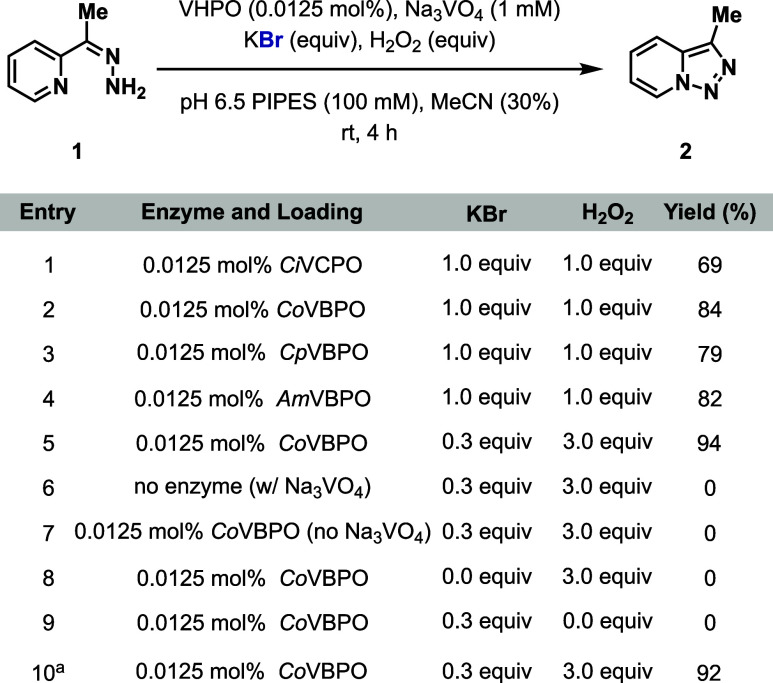
Optimization experiments for biocatalytic oxidative cyclization. Reaction conditions: **1** (4.0 μmol, 0.54 mg), VHPO (0.0125 mol %), Na_3_VO_4_ (1 mM), KBr (0.3–1.0 equiv), H_2_O_2_ (1.0–3.0 equiv), PIPES buffer (100 mM, pH 6.5, 200 μL), MeCN (300 μL), 1 mL total reaction volume, 4 h, rt. Yields determined by HPLC based on a calibration curve. See the Supporting Information for details. ^*a*^Performed with crude generation of **1**.

With optimized conditions in hand, the substrate scope for chemoenzymatic triazolopyridine synthesis was explored. The one-pot sequence accommodates 2-benzoylpyridine, delivering the corresponding triazolopyridine in 90% yield (Figure 3, **3**). The protocol can be readily applied to a range of phenyl-substituted 2-pyridyl ketones including those with methyl- and *tert*-butyl substitution at the *para* position of the aryl appendage, furnishing the corresponding triazolopyridine products in 90% and 93% yield, respectively (Figure 3, 4–5). A substrate containing an electron-donating methoxy group in the same position is tolerated in 89% yield (Figure 3, 6) with no competing overhalogenation observed. Additionally, a substrate containing an electron-withdrawing trifluoromethyl group at the *para* position of the aryl group is accommodated in 85% yield (Figure 3, 7). Methyl- and methoxy- substituents at the *meta* position afforded triazolopyridines in 82–85% yield (Figure 3, 8–9), while the *ortho*-substituted analogues are similarly efficient, giving 82–87% yields (Figure 3, 10–11). The reaction conditions were also compatible with 2,2’-dipyridylketone, providing the respective triazolopyridine in 84% yield (Figure 3, 12). Methyl and bromo substitutions on the pyridine ring of 2-acylpyridines are also tolerated under optimized reaction conditions, producing the corresponding pyridotriazoles in 80–87% yield (Figure 3, 13 and 14, respectively). Notably, 2-acetylpyrazine is also accommodated in 85% yield (Figure 3, 15). Furthermore, picolinaldehyde, quinoline-2-carbaldehyde, and isopropyl 2-pyridyl ketone substrates furnish the corresponding triazolopyridine products in 71–90% yields (Figure 3, 16–18).

**Figure 3:**
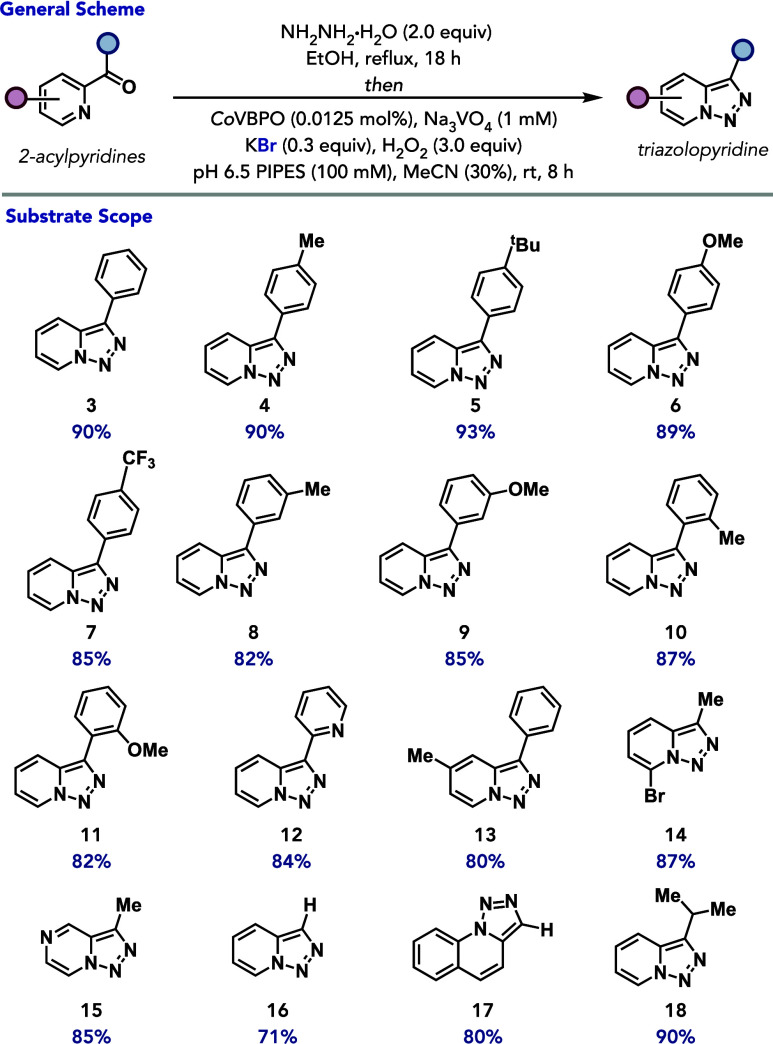
Substrate scope for chemoenzymatic oxidative cyclization. Reaction conditions: **substrate** (0.8 mmol, 1.0 equiv), NH_2_NH_2_·H_2_O (1.6 mmol, 2.0 equiv), EtOH (2 mL), reflux 18 h *then Co*VBPO (0.0125 mol %), Na_3_VO_4_ (1 mM final concentration), KBr (0.3 equiv), H_2_O_2_ (3.0 equiv), PIPES buffer (100 mM, pH 6.5), MeCN (30%), 8 h, rt. used. Yields determined by isolation. See the Supporting Information for more details.

A proposed mechanism for VHPO-mediated oxidative cyclization is outlined in Figure 4. The developed procedure begins with condensation between NH_2_NH_2_ and the starting 2-acylpyridine to generate the corresponding 2-pyridyl acylhydrazone (Figure 4, Step 1). In accordance with previously proposed mechanisms for VHPO halogenation reactions,^35–38^ the vanadate cofactor in VHPOs is bound to a histidine residue in the enzyme active site (**I**). Introduction of H_2_O_2_ leads to the formation of a peroxovanadium intermediate (**II**) that is primed for nucleophilic attack by bromide ion, generating vanadium-bound hypobromite (**III**). This species can either participate directly in a halogenation event or can be released from the coordination sphere to generate hypobromous acid (HOBr) as the brominating agent. We propose that one of these pathways is responsible for bromination of the previously generated 2-pyridyl acylhydrazone leading to the corresponding N-bromopyridyl hydrazone (**IV**) (Figure 4, Step 2). In analogy to other proposed mechanisms for oxidative cyclization of pyridyl hydrazones,^8–20^ this bromination event would ultimately lead to the formation of a pyridyl diazo intermediate (**V**), initiating a cyclization event to generate the corresponding triazolopyridine and releasing bromide to be recycled by the VHPO.

**Figure 4:**
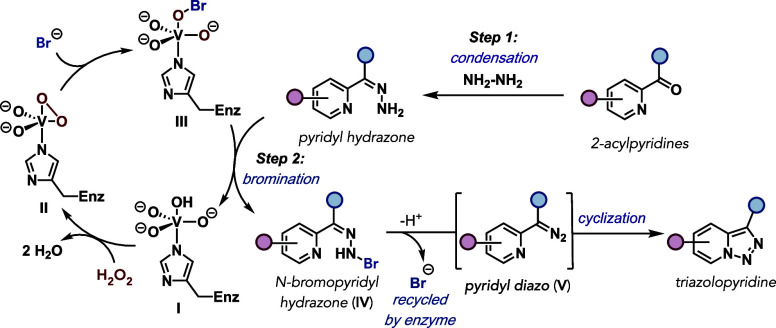
Proposed mechanism for VHPO-catalyzed oxidative cyclization of 2-pyridyl acylhydrazones.

Upon completion of reaction development, the synthetic utility of VHPO-catalyzed oxidative cyclization was examined. The optimized protocol was readily performed on gram-scale, providing 3-phenyl-[1,2,3]­triazolo­[1,5-*a*]­pyridine (**3**) in 89% yield (Figure 5a). Upon generation of gram-quantities of **3**, it was selected as a representative versatile synthon in a range of transformations (Figure 5b). It was first subjected to light-induced metal-free denitrogenative arylation with phenylboronic acid in the presence of potassium carbonate (K_2_CO_3_) under 390 nm light irradiation to give 2-benzhydrylpyridine (**20**) in 83% yield. Using the same light source, **3** underwent cyclopropanation with styrene to give 2-(1,2-diphenylcyclopropyl)­pyridine (**21**) in 78% yield and a 1:0.09 diasteromeric ratio.^59^ Triazolopyridine **3** was also used in a Lewis acid-catalyzed denitrogenative transannulation with benzonitrile in the presence of boron trifluoride diethyl etherate (BF_3_·OEt_2_) to afford 1,3-diphenylimidazo­[1,5-*a*]­pyridine (**22**) in 90% yield.^60^ Finally, a palladium-catalyzed regioselective denitrogenation of **3** with iodobenzene furnished the corresponding 2,6-disubstituted pyridine (**23**) in 72% yield.^61^ These transformations underscore the ability of triazolopyridines to participate in oxidative coupling reactions that construct highly substituted pyridine architectures. Together, these derivatization studies demonstrate the versatility of using [1,2,3]­triazolo­[1,5-*a*]­pyridines as privileged intermediates for accessing diverse heterocyclic scaffolds through chemoenzymatic reaction logic.

**Figure 5:**
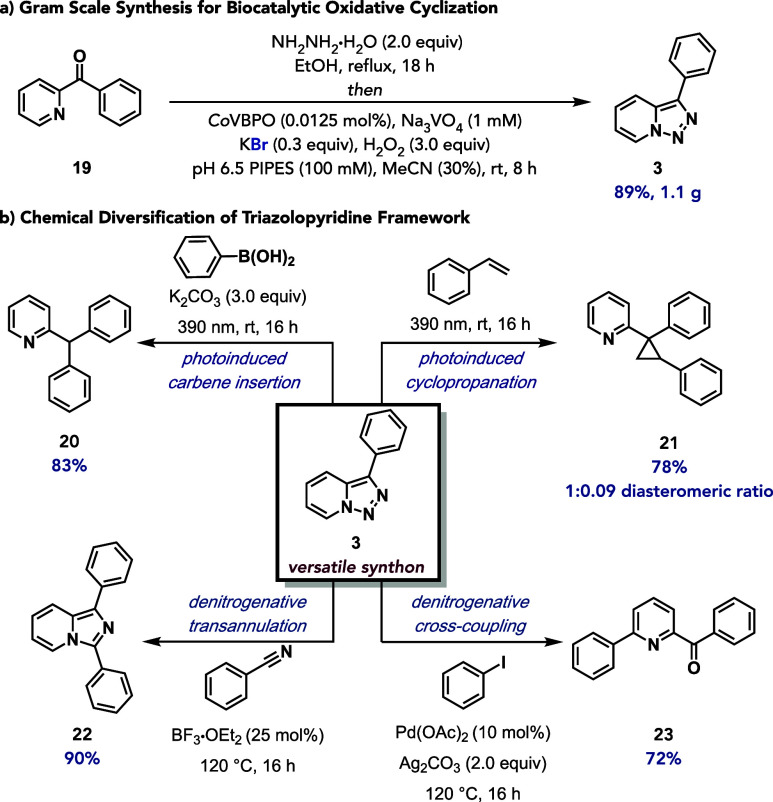
Synthetic utility of VHPO-catalyzed oxidative cyclization. (a) Gram-scale synthesis of 3-phenyl-[1,2,3]­triazolo­[1,5-*a*]­pyridine. Reaction conditions: **19** (6.4 mmol, 1.0 equiv), NH_2_NH_2_·H_2_O (12.8 mmol, 2.0 equiv), EtOH (25 mL), reflux 18 h *then Co*VBPO (0.0125 mol %), Na_3_VO_4_ (1 mM final concentration), H_2_O_2_ (3.0 equiv), PIPES buffer (100 mM, pH 6.5), MeCN (30%), 8 h, rt. used. (b) Chemical diversification of triazolopyridine framework. (i) Visible-light-induced arylation reaction of 3-phenyl-[1,2,3]­triazolo­[1,5-*a*]­pyridine. Reaction conditions: **3** (0.2 mmol, 1.0 equiv), K_2_CO_3_ (0.6 mmol, 3 equiv), phenylboronic acid (0.3 mmol, 1.5 equiv), benzene (2.0 mL), 390 nm, 16 h. (ii) Visible-light-induced cyclopropanation of 3-phenyl-[1,2,3]­triazolo­[1,5-*a*]­pyridine. Reaction conditions: **3** (0.2 mmol, 1.0 equiv), styrene (0.6 mmol, 3.0 equiv), 390 nm, 16 h. (iii) Lewis acid-catalyzed denitrogenative transannulation of 3-phenyl-[1,2,3]­triazolo­[1,5-*a*]­pyridine. Reaction conditions: **3** (0.20 mmol, 1.0 equiv), benzonitrile (0.24 mmol, 1.2 equiv), BF_3_·OEt_2_ (0.05 mmol, 0.25 equiv), 1,2-dichlorobenzene (0.20 mL), 1,2-dichloroethane (0.25 mL), 120 °C, 16 h. (iv) Pd-catalyzed C-6 functionalization of pyridine. Reaction conditions: **3** (0.50 mmol, 1.0 equiv), iodobenzene (1.0 mmol, 2.0 equiv), palladium­(II) acetate (10 mol %), and silver carbonate (1.0 mmol, 2.0 equiv), 120 °C, 16 h.

In conclusion, we have discovered that the VHPO enzymes are effective biocatalysts for the oxidative cyclization of 2-pyridyl acylhydrazones through cryptic diazo formation. The catalyst system is effective for the synthesis of a wide range of [1,2,3]­triazolo­[1,5a]­pyridines in high yield and with excellent chemoselectivity. These studies expand the synthetic repertoire of VHPOs in organic chemistry and provide a biocompatible platform for cryptic diazo formation in chemoenzymatic synthesis.

## Supplementary Material



## Data Availability

The data underlying this study are available in the published article and its Supporting Information.

## Notes

Detailed experimental procedures, NMR spectra, NMR data, Supplementary Figures S1–S5 (PDF)
