# Supplementary material for: Chemoenzymatic Triazolopyridine Synthesis Enabled by Cryptic Diazo Formation by Vanadium-Dependent Haloperoxidases
Source: Org Lett. 2026 Apr 13;28(16):5108–13. doi: 10.1021/acs.orglett.6c00937 (PMC13122641; doi:10.1021/acs.orglett.6c00937)

Supplementary Materials for

Chemoenzymatic Triazolopyridine Synthesis Enabled by Cryptic Diazo Formation by Vanadium-Dependent Haloperoxidases

Manik Sharma[+], Kyle F. Biegasiewicz[+*]

[+] Department of Chemistry, Emory University, Atlanta, Georgia 30322, USA

Corresponding Author E-mail: kbiegas@emory.edu

This PDF file includes:

Materials and Methods
Product Characterizations
Supplementary Text
Figs. S1-S5
Spectral Data
References

Table of Contents

General Experimental Information……………………………………..……………….…. 3

Synthesis and Characterization of 2-Acylpyridine Substrates…………………….…...……..4

General Procedures for VHPO-Catalyzed [1, 2, 3]Triazolo[1, 5-a]Pyridine Formation...15

Product Characterization for [1, 2, 3]Triazolo[1, 5-a]Pyridine Compounds……….…….. 17

Additional Reaction Procedures……………………………………………………........…33

Optimization Data……………………………………………………………….....…..... 38

References…………………………………………………………………….…..…..…. 41

Spectroscopic Data…………………………………………………………..…......… 42

General Experimental Information

General: Unless specified, all reagents and solvents used in this study were purchased from commercial suppliers and used as received (Combi-Blocks, Sigma-Aldrich, Oakwood Chemicals, Fischer Scientific, VWR). All nonaqueous reactions were performed using glassware that was flame-dried and capped with a rubber septum under nitrogen atmosphere using an inlet and outlet needle connected to a mineral oil bubbler. All aqueous reactions were conducted using glassware without flame-drying prior to experimental set up and without nitrogen atmosphere. For experiments requiring dried or degassed solvent, it was obtained from a solvent purification system from Pure Process Technology. Unless otherwise indicated, deionized water (H_2O) was used in any experiments where H_2O is included in the procedure. Ultrapure Milli-Q water (Milli-Q H_2O) was accessed through a Milli-Q® EQ 7000 Ultrapure Water Purification System.

Chromatography: Flash chromatography was performed on SiliaFlash® P60 (230-400 mesh, particle size 0.040-0.063 mm) using the listed solvent systems in each procedure. Thin-layer chromatography (TLC) was performed using Uniplate HLF 250 micron F254 precoated glass plates and preparative TLC was performed on Uniplate GF 1000 micron F254 precoated glass plates. For TLC analysis, a short-wave UV lamp and/or plate staining was used.

Spectroscopy Anaylsis: ^{1}H- and ^{13}C-NMR were obtained on a Bruker AVIII or Bruker NEO (400 and 101 MHz, respectively). Chemical shifts are reported in ppm (δ) downfield from tetramethylsilane and are internally referenced to the internal deuterated solvent indicated. ^{1}H-NMR data is reported as follows: chemical shift [multiplicity, coupling constant (Hz), number of hydrogens]. Multiplicities are reported as follows: s (singlet), b (broad signal), d (doublet), dd (doublet of doublets), ddd (doublet of doublet of doublets), t (triplet), dt (doublet of triplets), tt (triplet of triplets), q (quartet), dq (doublet of quartets), p (pentet), m (multiplet). High-resolution mass spectra were obtained on a Thermo Finnigan LTQ-FTMS spectrometer using APCI with an orbitrap mass analyzer.

Analytical: Analytical high-performance liquid chromatography (HPLC) was carried out using a Shimadzu LCMS-2020 System with a Kromasil EternityXT-2.5-C18 column (Dimensions: 4.6x50mm, Batch/Serial: 0000016627/A, Part No. XH2CLA05).

Protein Expression and Purification: All protein expression and purification were performed using previously reported methods.[1]

Synthesis and Characterization of 2-Acylpyridine Substrates

General Procedure for the Preparation of 2-Acylpyridine Substrates (General Procedure A):

The following procedure was adapted according to a literature procedure with slight modifications.[2] A solution containing magnesium (19.5 mmol, 1.3 equiv), 15 mg I_2 in dry THF (30 mL) was treated with corresponding bromobenzene derivative (15 mmol, 1.0 equiv) stirred at room temperature under inert atmosphere. After the formation of the Grignard reagent (the color changed to gray), the corresponding picolinonitrile derivative (15 mmol, 1.0 equiv) was dissolved in dry THF (10 mL), which was dropwise added into the mixture solution of Grignard reagent at 0 °C. Upon addition of picolinonitrile derivative, the reaction mixture was further stirred at room temperature for 6 h. After the indicated time, the reaction mixture was quenched using saturated aqueous NH_4Cl (100 mL) and extracted with ethyl acetate (2 x 50 mL). After evaporation under reduced pressure, the residue was re-dissolved in diethyl ether (100 mL) and 6 M HCl (10 mL) was added into the solution. After 30 min of stirring at room temperature, the organic layer was separated. The aqueous layer was basified with saturated $NaHCO_3$ and then extracted with ethyl acetate (3 x 60 mL). The combined organic extracts were washed with 100 mL of brine, then dried over anhydrous sodium sulfate, and concentrated under reduced pressure. The resulting crude residue was purified by flash column chromatography, yielding the desired substrate.

Pyridin-2-yl(p-tolyl)methanone (SM-2)[2]

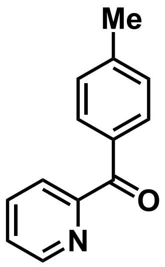

Synthesized from commercially available picolinonitrile and 1-bromo-4-methylbenzene following General Procedure A and was obtained as a yellow oil.

Yield: 78% (2.31 g)

Purification: Eluted with 20% EtOAc in Hexanes

^{1}H NMR (400 MHz, $CDCl_3$) δ 8.71 (ddd, J = 4.8, 1.7, 0.9 Hz, 1H), 8.03 – 7.94 (m, 3H), 7.88 (td, J = 7.7, 1.8 Hz, 1H), 7.47 (ddd, J = 7.6, 4.8, 1.3 Hz, 1H), 7.28 (d, J = 8.0 Hz, 2H), 2.42 (s, 3H).

^{13}C NMR (101 MHz, $CDCl_3$) δ 193.7, 155.5, 148.6, 143.9, 137.1, 133.7, 131.4, 129.0, 126.1, 124.7, 21.9.

(4-(tert-Butyl)phenyl)(pyridin-2-yl)methanone (SM-3)[3]

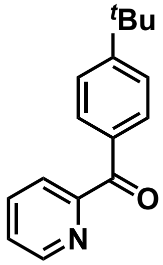

Synthesized from commercially available picolinonitrile and 1-bromo-4-(tert-butyl)benzene following General Procedure A and was obtained as a light yellow solid.

Yield: 72% (2.58 g)

Purification: Eluted with 20% EtOAc in Hexanes

^{1}H NMR (400 MHz, $CDCl_3$) δ 8.72 (ddd, *J* = 4.8, 1.7, 0.9 Hz, 1H), 8.04 – 8.00 (m, 3H), 7.89 (td, *J* = 7.7, 1.7 Hz, 1H), 7.52 – 7.46 (m, 3H), 1.35 (s, 9H).

^{13}C NMR (101 MHz, $CDCl_3$) δ 193.7, 156.8, 155.5, 148.6, 137.2, 131.1, 126.1, 125.4, 124.7, 35.3, 31.2.

(4-Methoxyphenyl)(pyridin-2-yl)methanone (SM-4)[3]

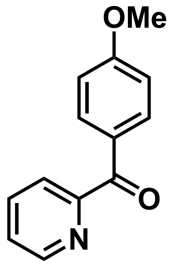

Synthesized from commercially available picolinonitrile and 1-bromo-4-methoxybenzene following General Procedure A and was obtained as a light brown solid.

Yield: 85% (2.72 g)

Purification: Eluted with 30% EtOAc in Hexanes

^{1}H NMR (400 MHz, $CDCl_3$) δ 8.73 – 8.65 (m, 1H), 8.13 – 8.06 (m, 2H), 7.96 (d, J = 7.8 Hz, 1H), 7.85 (td, J = 7.7, 1.8 Hz, 1H), 7.44 (ddd, J = 7.6, 4.8, 1.3 Hz, 1H), 6.97 – 6.91 (m, 2H), 3.85 (s, 3H).

^{13}C NMR (101 MHz, $CDCl_3$) δ 192.3, 163.6, 155.8, 148.4, 137.1, 133.5, 129.0, 125.9, 124.6, 113.6, 55.5.

Pyridin-2-yl(4-(trifluoromethyl)phenyl)methanone (SM-5)[2]

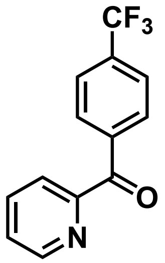

Synthesized from commercially available picolinonitrile and 1-bromo-4 (trifluoromethyl)benzene following General Procedure A and was obtained as a light yellow liquid.

Yield: 70% (2.64 g)

Purification: Eluted with 20% EtOAc in Hexanes

^{1}H NMR (400 MHz, $CDCl_3$) δ 8.72 (ddd, *J* = 4.8, 1.8, 0.9 Hz, 1H), 8.21 – 8.15 (m, 2H), 8.13 (dt, *J* = 7.9, 1.1 Hz, 1H), 7.93 (td, *J* = 7.7, 1.8 Hz, 1H), 7.77 – 7.72 (m, 2H), 7.52 (ddd, *J* = 7.6, 4.8, 1.3 Hz, 1H).

^{13}C NMR (101 MHz, $CDCl_3$) δ 192.9, 154.2, 148.8, 139.5, 137.4, 134.0 (q, *J* = 32.7 Hz), 131.3, 126.9, 125.2 (q, *J* = 3.9 Hz), 124.9, 123.8 (q, *J* = 273.9 Hz).

Pyridin-2-yl(m-tolyl)methanone (SM-6)[2]

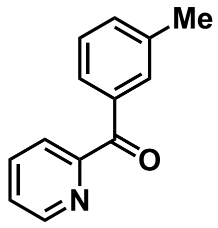

Synthesized from commercially available picolinonitrile and 1-bromo-3-methylbenzene following General Procedure A and was obtained as a yellow oil.

Yield: 81% (2.40 g)

Purification: Eluted with 20% EtOAc in Hexanes

^{1}H NMR (400 MHz, $CDCl_3$) δ 8.72 (d, J = 4.8 Hz, 1H), 8.03 – 7.98 (m, 1H), 7.88 (td, J = 7.7, 1.7 Hz, 1H), 7.83 (d, J = 7.4 Hz, 2H), 7.49 – 7.44 (m, 1H), 7.42 – 7.33 (m, 2H), 2.41 (s, 3H).

^{13}C NMR (101 MHz, $CDCl_3$) δ 194.4, 155.4, 148.7, 138.1, 137.1, 136.4, 133.9, 131.4, 128.4, 128.7, 126.2, 124.7, 21.5.

(3-Methoxyphenyl)(pyridin-2-yl)methanone (SM-7)[2]

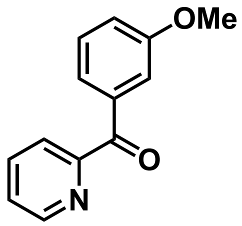

Synthesized from commercially available picolinonitrile and 1-bromo-3-methoxybenzene following General Procedure A and was obtained as a white solid.

Yield: 86% (2.75 g)

Purification: Eluted with 20% EtOAc in Hexanes

^{1}H NMR (400 MHz, $CDCl_3$) δ 8.72 (ddd, *J* = 4.8, 1.8, 0.9 Hz, 1H), 8.02 (dt, *J* = 7.9, 1.1 Hz, 1H), 7.89 (td, *J* = 7.7, 1.7 Hz, 1H), 7.66 – 7.55 (m, 2H), 7.48 (ddd, *J* = 7.6, 4.8, 1.3 Hz, 1H), 7.39 (t, *J* = 7.9 Hz, 1H), 7.14 (ddd, *J* = 8.2, 2.7, 1.0 Hz, 1H), 3.86 (s, 3H).

^{13}C NMR (101 MHz, $CDCl_3$) δ 193.8, 159.5, 155.2, 148.7, 137.6, 137.2, 129.3, 126.3, 124.7, 124.0, 119.6, 115.2, 55.6.

Pyridin-2-yl(o-tolyl)methanone (SM-8)[2]

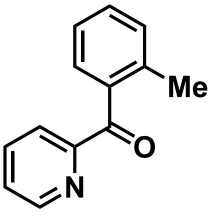

Synthesized from commercially available picolinonitrile and 1-bromo-2-methylbenzene following General Procedure A and was obtained as a white solid.

Yield: 80% (2.37 g)

Purification: Eluted with 20% EtOAc in Hexanes

^{1}H NMR (400 MHz, $CDCl_3$) δ 8.69 (ddd, *J* = 4.8, 1.7, 0.9 Hz, 1H), 8.08 (dt, *J* = 7.9, 1.2 Hz, 1H), 7.88 (td, *J* = 7.7, 1.8 Hz, 1H), 7.48 – 7.37 (m, 3H), 7.30 – 7.23 (m, 2H), 2.37 (s, 3H).

^{13}C NMR (101 MHz, $CDCl_3$) δ 197.5, 155.1, 149.2, 137.9, 137.1, 131.2, 130.1, 126.6, 125.2, 124.2, 20.6.

(2-Methoxyphenyl)(pyridin-2-yl)methanone (SM-9)[3]

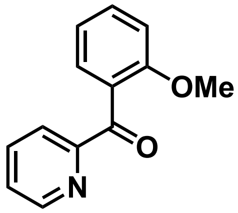

Synthesized from commercially available picolinonitrile and 1-bromo-2-methoxybenzene following General Procedure A and was obtained as a white solid.

Yield: 83% (2.65 g)

Purification: Eluted with 20% EtOAc in Hexanes

^{1}H NMR (400 MHz, $CDCl_3$) δ 8.64 (dt, *J* = 4.9, 1.3 Hz, 1H), 7.98 (dt, *J* = 7.8, 1.3 Hz, 1H), 7.84 (td, *J* = 7.7, 1.7 Hz, 1H), 7.53 (dd, *J* = 7.6, 1.8 Hz, 1H), 7.48 (ddd, *J* = 8.9, 7.5, 1.8 Hz, 1H), 7.41 (ddd, *J* = 7.6, 4.8, 1.3 Hz, 1H), 7.05 (td, *J* = 7.5, 0.9 Hz, 1H), 6.97 (d, *J* = 8.3 Hz, 1H), 3.64 (s, 3H).

^{13}C NMR (101 MHz, $CDCl_3$) δ 196.2, 158.3, 155.5, 149.0, 136.7, 132.9, 130.4, 128.2, 126.2, 123.2, 120.6, 111.7, 55.7.

(4-Methylpyridin-2-yl)(phenyl)methanone (SM-10)[2]

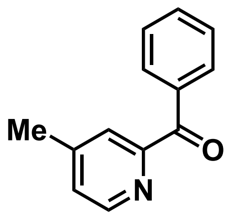

Synthesized from commercially available4-methylpicolinonitrile and bromobenzene following General Procedure A and was obtained as a yellow oil.

Yield: 80% (2.37 g)

Purification: Eluted with 15% EtOAc in Hexanes

^{1}H NMR (400 MHz, $CDCl_3$) δ 8.57 (d, *J* = 5.0 Hz, 1H), 8.08 – 8.01 (m, 2H), 7.86 (dd, *J* = 1.7, 0.9 Hz, 1H), 7.62 – 7.54 (m, 1H), 7.48 (dd, *J* = 8.3, 7.0 Hz, 2H), 7.33 – 7.29 (m, 1H), 2.46 (s, 3H).

^{13}C NMR (101 MHz, $CDCl_3$) δ 194.4, 155.1, 148.6, 148.5, 133.0, 131.1, 128.3, 127.2, 125.5, 21.3.

Synthesis and Characterization of 2-(1-Hydrazineylideneethyl)pyridine (1)

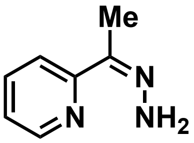

The following procedure was adapted according to a literature procedure.[4] A solution of hydrazine hydrate (64-65% in water, 30.0 mmol, 3.0 equiv) was added, to the solution containing 2-acetylpyridine (10.0 mmol, 1.0 equiv) and ethanol (30 mL). The reaction mixture was refluxed for 8 h. After the indicated time, the reaction mixture was allowed to cool to room temperature, and the ethanol was evaporated under reduced pressure. The resulting crude residue was extracted with ethyl acetate (3 x 50 mL). The combined organic layers were then washed with brine (50 mL), dried over sodium sulfate (Na_2SO_4), and concentrated under reduced pressure. The resulting crude sample was purified recrystallized using dichloromethane and hexanes to obtain the corresponding pure product as a white solid. The characterization data is in agreement with literature precedent.[4]

Yield: 95% (1.28 g)

^{1}H NMR (400 MHz, $CDCl_3$) δ 8.54 (ddd, *J* = 4.9, 1.9, 1.0 Hz, 1H), 7.92 (dt, *J* = 8.1, 1.1 Hz, 1H), 7.63 (ddd, *J* = 8.2, 7.4, 1.8 Hz, 1H), 7.16 (ddd, *J* = 7.4, 4.9, 1.2 Hz, 1H), 5.54 (s, 2H), 2.25 (s, 3H).

^{13}C NMR (101 MHz, $CDCl_3$) δ 156.6, 148.5, 147.8, 136.2, 122.7, 119.8, 9.7.

General Procedures for VHPO-Catalyzed [1, 2, 3]Triazolo[1, 5-a]Pyridine Formation

General Analytical Procedure for the Synthesis of [1, 2, 3]Triazolo[1, 5-a]Pyridines from Hydrazones (General Procedure B):

An enzyme aliquot of the VHPO from *Corallina officinalis* (*Co*VBPO, 10 µM, 50 µL) was removed from a -80 °C freezer and warmed to room temperature over 5 min. After thawing, a 250 mM solution of aqueous Na_3VO_4 (4 µL) was added to the enzyme aliquot, and the resulting mixture was centrifuged for 10 seconds using a Chemglass Life Sciences MLX-108-CLS mini centrifuge and then placed at room temperature until further use. To a 1-dram vial was then added H_2O purified by an Elga purification system (435 µL), 500 mM pH 6.5 PIPES buffer (200 µL), and 176 mM aqueous KBr (6.82 µL, 0.3 equiv) followed by addition of 200 µL MeCN. A 40 mM solution of corresponding hydrazone in MeCN (100 µL, 1.0 equiv, 0.004 mmol substrate) was added. The aliquot containing the *Co*VBPO (0.025 mol%, 0.5 µM in reaction) and Na_3VO_4 (0.25 equiv) was then added to the reaction mixture followed by the addition of a 10% stock of H_2O_2 (3.95 µL, 3.0 equiv). The vial was then capped and placed on a shaker at room temperature for 4 h. After this time, the reaction mixture was diluted with MeCN (650 µL), transferred to an Eppendorf tube, and centrifuged in a Benchmark MC-24TM Touch Centrifuge at 12,500 rpm for 5 min. After centrifugation, 650 µL of the top layer of the reaction mixture was transferred to an LCMS vial, which was then placed on an LCMS for analysis.*

**100 µL of 1,3,5-trimethoxybenzene was added as an internal standard for yield confirmation, where applicable.*

General Preparative Procedure for the Synthesis of [1, 2, 3]Triazolo[1, 5-a]Pyridines from 2-Acylpyridines (General Procedure C):

A solution of hydrazine hydrate (64-65% in water, 1.6 mmol) was added to a 1-dram vial containing a mixture of 2-acylpyridine (0.8 mmol) and ethanol (2 mL) at room temperature. The reaction was then refluxed for 18 h. After this time, the reaction mixture was allowed to cool to room temperature and transferred to a 250 mL round-bottom flask containing MeCN (60 mL). An enzyme aliquot of VHPO from *Corallina officinalis* (*Co*VBPO, 10 µM, 10 mL) was taken from a -80 °C freezer, thawed at room temperature for 10 minutes, mixed with 800 µL of a 250 mM aqueous solution of Na_3VO_4, and allowed to sit at room temperature for 30 minutes. The crude hydrazone mixture in the 250 mL flask was then combined with water purified by an Elga purification system (87 mL), 500 mM pH 6.5 PIPES (40 mL), and 176 mM aqueous KBr (1.36 mL, 0.3 equiv). The contents of the centrifuge tube containing *Co*VBPO (0.0125 mol%, 0.5 µM in reaction) and Na_3VO_4 (0.25 equiv) was then added to the reaction mixture followed by addition of 10% stock of H_2O_2 (790 µL, 3.0 equiv). The reaction was stirred for 8 h at 900 rpm at room temperature. Upon completion, the reaction mixture was washed three times with ethyl acetate (3 x 40 mL) to extract the product. The combined organic layers were then washed with brine (70 mL), dried over Na_2SO_4, and concentrated under reduced pressure. The resulting crude sample was purified via flash column chromatography the pure product.

Product Characterization for [1,2,3]triazolo[1,5-a]pyridines

3-Methyl-[1,2,3]triazolo[1,5-a]pyridine (2)[4]

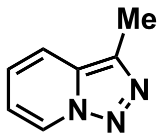

Synthesized from commercially available 2-acetylpyridine following General Procedure C and was obtained as a white solid.

Yield: 91% (96.9 mg)

Purification: Eluted in 40% EtOAc in Hexanes

^{1}H NMR (400 MHz, $CDCl_3$) δ 8.60 (dq, J = 7.1, 1.0 Hz, 1H), 7.58 (dt, J = 8.9, 1.2 Hz, 1H), 7.13 (ddd, J = 8.9, 6.6, 1.0 Hz, 1H), 6.89 (td, J = 6.7, 1.2 Hz, 1H), 2.58 (d, J = 1.0 Hz, 3H).

^{13}C NMR (101 MHz, $CDCl_3$) δ 134.4, 131.6, 125.1, 123.7, 117.6, 115.0, 10.4.

Standard Curve for Analytical Runs

Procedure for using standard curve is as follows: 1,3,5-trimethoxybenzene (8 mg/mL solution, 100 μL) is added to 900 μL of the reaction mixture and yield is determined by LCMS analysis based on the below standard curve. LCMS conditions: 2.5 μL injection volume, 0.5 mL/min mobile phase rate, 10-98% solvent B over 6.25 min. Mobile Phase: Solvent A – H_2O w/ 0.1% formic acid, Solvent B – MeCN w/ 0.1% formic acid.

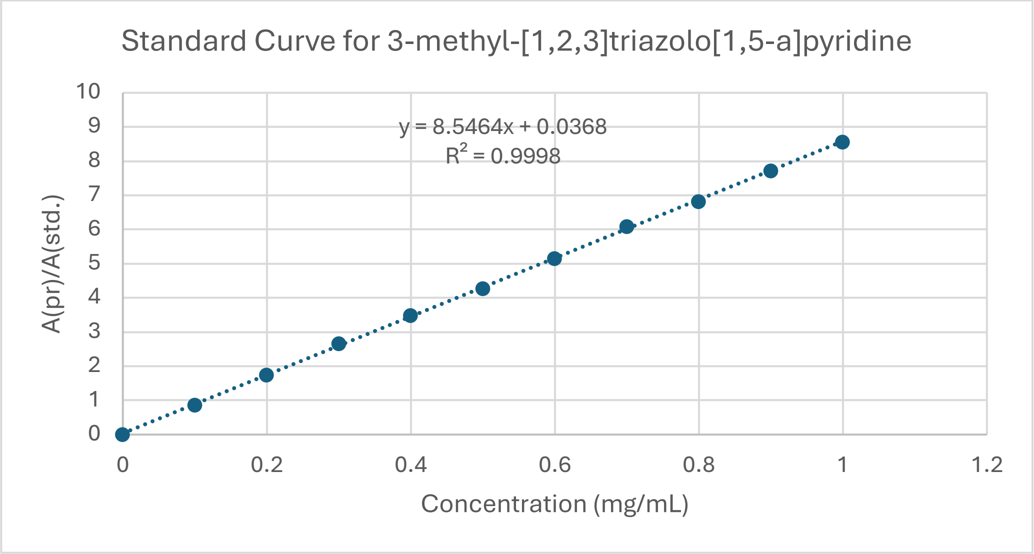

3-Phenyl-[1,2,3]triazolo[1,5-a]pyridine (3)[4]

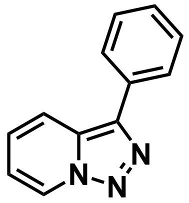

Synthesized from commercially available 2-benzoylpyridine following General Procedure C and was obtained as a white solid.

Yield: 90% (140.1 mg)

Purification: Eluted in 40% EtOAc in Hexanes

^{1}H NMR (400 MHz, $CDCl_3$) δ 8.73 (dt, *J* = 7.1, 1.1 Hz, 1H), 8.00 – 7.93 (m, 3H), 7.50 (dd, *J* = 8.4, 7.0 Hz, 2H), 7.41 – 7.35 (m, 1H), 7.28 (ddd, *J* = 9.0, 6.7, 1.0 Hz, 1H), 6.98 (td, *J* = 6.8, 1.2 Hz, 1H).

^{13}C NMR (101 MHz, $CDCl_3$) δ 138.1, 131.6, 130.6, 129.2, 128.1, 126.8, 125.8, 118.6, 115.4.

3-(p-Tolyl)-[1,2,3]triazolo[1,5-a]pyridine (4)[4]

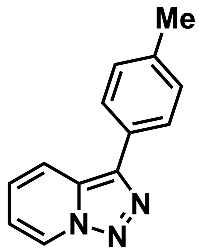

Synthesized from Pyridin-2-yl(p-tolyl)methanone (SM-2) following General Procedure C and was obtained as a white solid.

Yield: 90% (150.3 mg)

Purification: Eluted in 30% EtOAc in Hexanes

^{1}H NMR (400 MHz, $CDCl_3$) δ 8.74 (dd, J = 7.1, 1.1 Hz, 1H), 8.00 (dt, J = 9.0, 1.2 Hz, 1H), 7.81 (d, J = 1.9 Hz, 1H), 7.73 (d, J = 7.7 Hz, 1H), 7.40 (t, J = 7.7 Hz, 1H), 7.29 (ddd, J = 8.9, 6.6, 1.0 Hz, 1H), 7.21 (d, J = 7.6 Hz, 1H), 6.99 (td, J = 6.8, 1.2 Hz, 1H), 2.45 (s, 3H).

^{13}C NMR (101 MHz, $CDCl_3$) δ 138.9, 138.2, 131.4, 130.6, 129.0, 128.8, 127.5, 125.7, 125.6, 123.8, 118.6, 115.4, 21.7.

3-(4-(tert-Butyl)phenyl)-[1,2,3]triazolo[1,5-a]pyridine (5)

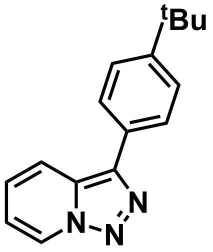

Synthesized from (4-(tert-butyl)phenyl)(pyridin-2-yl)methanone (SM-3) following General Procedure C and was obtained as a light yellow solid.

Yield: 93% (186.9 mg)

Purification: Recrystallized using Dichloromethane & Hexanes

^{1}H NMR (400 MHz, $CDCl_3$) δ 8.72 (dt, J = 7.1, 1.1 Hz, 1H), 7.98 (dt, J = 9.0, 1.2 Hz, 1H), 7.92 – 7.86 (m, 2H), 7.56 – 7.50 (m, 2H), 7.29 – 7.24 (m, 1H), 6.97 (td, J = 6.8, 1.2 Hz, 1H), 1.38 (s, 9H).

^{13}C NMR (101 MHz, $CDCl_3$) δ 151.1, 138.1, 130.4, 128.7, 126.5, 126.0, 125.4, 118.6, 115.3, 34.8, 31.4.

HRMS (APCI) m/z: calculated for $C_{16}H_{18}N_3$ $[M+H]^+$: 252.1495. Found: 252.1494.

3-(4-Methoxyphenyl)-[1,2,3]triazolo[1,5-a]pyridine (6)[4]

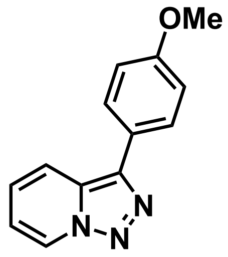

Synthesized from (4-methoxyphenyl)(pyridin-2-yl)methanone (SM-4) following General Procedure C and was obtained as a white solid.

Yield: 89% (160.4 mg)

Purification: Eluted in 40% EtOAc in Hexanes

^{1}H NMR (400 MHz, $CDCl_3$) δ 8.70 (d, J = 7.1 Hz, 1H), 7.93 (dd, J = 8.9, 1.3 Hz, 1H), 7.89 – 7.83 (m, 2H), 7.27 – 7.22 (m, 1H), 7.06 – 7.01 (m, 2H), 6.96 (td, J = 6.9, 1.2 Hz, 1H), 3.86 (s, 3H).

^{13}C NMR (101 MHz, $CDCl_3$) δ 159.5, 138.0, 130.1, 128.0, 125.6, 125.3, 124.1, 118.5, 115.3, 114.6, 55.5.

3-(4-(Trifluoromethyl)phenyl)-[1,2,3]triazolo[1,5-a]pyridine (7)[4]

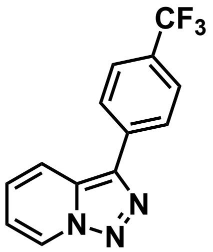

Synthesized from pyridin-2-yl(4-(trifluoromethyl)phenyl)methanone (SM-5) following General Procedure C and was obtained as a white solid.

Yield: 85% (178.9 mg)

Purification: Recrystallized using Dichloromethane & Hexanes

^{1}H NMR (400 MHz, $CDCl_3$) δ 8.77 (dt, J = 7.1, 1.1 Hz, 1H), 8.12 – 8.04 (m, 2H), 8.00 (dt, J = 9.0, 1.2 Hz, 1H), 7.74 (d, J = 8.1 Hz, 2H), 7.38 (ddd, J = 9.0, 6.7, 1.0 Hz, 1H), 7.05 (td, J = 6.8, 1.2 Hz, 1H).

^{13}C NMR (101 MHz, $CDCl_3$) δ 136.5, 135.1, 130.9, 129.7 (q, J = 32.7 Hz), 126.7, 126.6, 126.1 (q, J = 3.8 Hz), 126.0, 124.3 (q, J = 273.2 Hz), 118.2, 115.7.

3-(m-Tolyl)-[1,2,3]triazolo[1,5-a]pyridine (8)

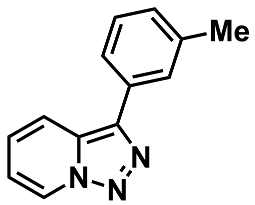

Synthesized from Pyridin-2-yl(m-tolyl)methanone (SM-6) following General Procedure C and was obtained as a white solid.

Yield: 82% (137.3 mg)

Purification: Eluted in 30% EtOAc in Hexanes

^{1}H NMR (400 MHz, $CDCl_3$) δ 8.72 (dt, J = 7.1, 1.1 Hz, 1H), 7.97 (dt, J = 9.0, 1.2 Hz, 1H), 7.88 – 7.80 (m, 2H), 7.31 (d, J = 8.0 Hz, 2H), 7.29 – 7.24 (m, 1H), 6.97 (td, J = 6.8, 1.2 Hz, 1H), 2.41 (s, 3H).

^{13}C NMR (101 MHz, $CDCl_3$) δ 138., 137.9, 130.4, 129.8, 128.7, 126.7, 125.6, 125.4, 118.6, 115.3, 21.4.

HRMS (APCI) m/z: calculated for $C_{13}H_{12}N_3$ $[M+H]^+$: 210.1026. Found: 210.1025.

3-(3-Methoxyphenyl)-[1,2,3]triazolo[1,5-a]pyridine (9)

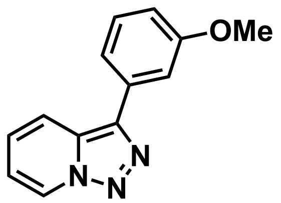

Synthesized from (3-methoxyphenyl)(pyridin-2-yl)methanone (SM-7) following General Procedure C and was obtained as a colorless oil.

Yield: 85% (153.2 mg)

Purification: Eluted in 40% EtOAc in Hexanes

^{1}H NMR (400 MHz, $CDCl_3$) δ 8.74 (dd, *J* = 7.1, 1.1 Hz, 1H), 8.00 (dt, *J* = 9.0, 1.2 Hz, 1H), 7.55 (t, *J* = 2.1 Hz, 1H), 7.50 (dt, *J* = 7.6, 1.3 Hz, 1H), 7.41 (t, *J* = 7.9 Hz, 1H), 7.30 (ddd, *J* = 9.0, 6.6, 1.0 Hz, 1H), 7.00 (td, *J* = 6.9, 1.2 Hz, 1H), 6.94 (ddd, *J* = 8.2, 2.7, 1.0 Hz, 1H), 3.90 (s, 3H).

^{13}C NMR (101 MHz, $CDCl_3$) δ 160.3, 138.0, 132.9, 130.7, 130.1, 125.8, 125.8, 119.0, 118.6, 115.5, 114.0, 112.0, 55.5.

HRMS (APCI) m/z: calculated for $C_{13}H_{12}N_3O$ $[M+H]^+$: 226.0975. Found: 226.0974.

3-(o-Tolyl)-[1,2,3]triazolo[1,5-a]pyridine (10)

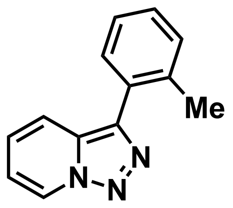

Synthesized from Pyridin-2-yl(o-tolyl)methanone (SM-8) following General Procedure C and was obtained as a white solid.

Yield: 87% (145.6 mg)

Purification: Eluted in 30% EtOAc in Hexanes

^{1}H NMR (400 MHz, $CDCl_3$) δ 8.74 (dt, *J* = 7.1, 1.1 Hz, 1H), 7.66 (dt, *J* = 8.9, 1.2 Hz, 1H), 7.45 (dd, *J* = 7.1, 1.9 Hz, 1H), 7.38 – 7.21 (m, 4H), 6.99 (td, *J* = 6.8, 1.3 Hz, 1H), 2.43 (s, 3H).

^{13}C NMR (101 MHz, $CDCl_3$) δ 138.5, 137.5, 131.5, 131.1, 130.1, 130.0, 128.5, 126.0, 125.4, 125.2, 118.3, 115.3, 20.6.

HRMS (APCI) m/z: calculated for $C_{13}H_{12}N_3$ $[M+H]^+$: 210.1026. Found: 210.1025.

3-(2-Methoxyphenyl)-[1,2,3]triazolo[1,5-a]pyridine (11)

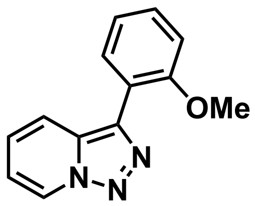

Synthesized from (2-methoxyphenyl)(pyridin-2-yl)methanone (SM-9) following General Procedure C and was obtained as a colorless oil.

Yield: 82% (147.8 mg)

Purification: Eluted in 40% EtOAc in Hexanes

^{1}H NMR (400 MHz, $CDCl_3$) δ 8.70 (d, J = 7.1 Hz, 1H), 7.92 – 7.78 (m, 2H), 7.40 (ddd, J = 8.2, 7.4, 1.8 Hz, 1H), 7.20 (ddd, J = 9.0, 6.6, 1.0 Hz, 1H), 7.11 (td, J = 7.5, 1.1 Hz, 1H), 7.04 (dd, J = 8.3, 1.1 Hz, 1H), 6.95 (td, J = 6.8, 1.3 Hz, 1H), 3.86 (s, 3H).

^{13}C NMR (101 MHz, $CDCl_3$) δ 156.4, 135.7, 131.8, 131.2, 129.7, 125.2, 124.4, 121.3, 120.5, 115.2, 111.3, 55.5.

HRMS (APCI) m/z: calculated for $C_{13}H_{12}N_3O$ $[M+H]^+$: 226.0975. Found: 226.0974.

3-(Pyridin-2-yl)-[1,2,3]triazolo[1,5-a]pyridine (12)[4]

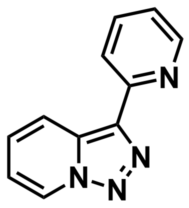

Synthesized from commercially available di(pyridin-2-yl)methanone following General Procedure C and was obtained as a white solid.

Yield: 84% (131.9 mg)

Purification: Eluted in 70% EtOAc in Hexanes

^{1}H NMR (400 MHz, $CDCl_3$) δ 8.70 (ddd, *J* = 17.2, 8.0, 1.3 Hz, 2H), 8.63 (dt, *J* = 5.0, 1.2 Hz, 1H), 8.32 (dd, *J* = 8.0, 1.3 Hz, 1H), 7.76 (td, *J* = 7.8, 1.8 Hz, 1H), 7.33 (dd, *J* = 8.9, 6.6 Hz, 1H), 7.18 (ddd, *J* = 7.6, 4.9, 1.2 Hz, 1H), 7.00 (td, *J* = 6.8, 1.3 Hz, 1H).

^{13}C NMR (101 MHz, $CDCl_3$) δ 152.1, 149.4, 137.5, 136.6, 132.0, 126.4, 125.2, 122.0, 121.3, 120.4, 115.9.

5-Methyl-3-phenyl-[1,2,3]triazolo[1,5-a]pyridine (13)

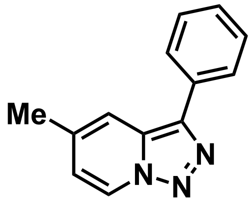

Synthesized from (4-methylpyridin-2-yl)(phenyl)methanone (SM-10) following General Procedure C and was obtained as a white solid.

Yield: 80% (133.9 mg)

Purification: Eluted in 30% EtOAc in Hexanes

^{1}H NMR (400 MHz, $CDCl_3$) δ 8.58 (dd, *J* = 7.2, 1.0 Hz, 1H), 7.95 – 7.89 (m, 2H), 7.69 (q, *J* = 1.3 Hz, 1H), 7.48 (dd, *J* = 8.5, 7.0 Hz, 2H), 7.38 – 7.32 (m, 1H), 6.78 (dd, *J* = 7.2, 1.7 Hz, 1H), 2.43 (d, *J* = 1.2 Hz, 3H).

^{13}C NMR (101 MHz, $CDCl_3$) δ 136.9, 136.7, 131.8, 130.8, 129.0, 127.7, 126.5, 124.8, 118.1, 116.4, 21.5.

HRMS (APCI) m/z: calculated for $C_{13}H_{12}N_3$ $[M+H]^+$: 210.1026. Found: 210.1027.

7-Bromo-3-methyl-[1,2,3]triazolo[1,5-a]pyridine (14)[4]

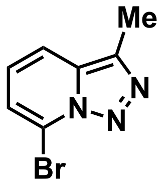

Synthesized from commercially available 2-acetyl-6-bromopyridine following General Procedure C and was obtained as a light yellow solid.

Yield: 87% (147.5 mg)

Purification: Eluted in 40% EtOAc in Hexanes

^{1}H NMR (400 MHz, $CDCl_3$) δ 7.58 (dd, *J* = 8.8, 1.1 Hz, 1H), 7.14 (dd, *J* = 7.1, 1.1 Hz, 1H), 7.04 (dd, *J* = 8.7, 7.0 Hz, 1H), 2.57 (s, 3H).

^{13}C NMR (101 MHz, $CDCl_3$) δ 136.4, 133.3, 124.4, 119.2, 116.6, 115.2, 10.8.

3-Methyl-[1,2,3]triazolo[1,5-a]pyrazine (15)

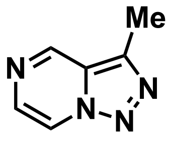

Synthesized from commercially available 2-acetylpyrazine following General Procedure C and was obtained as a white solid.

Yield: 85% (91.2 mg)

Purification: Eluted in 100% EtOAc

^{1}H NMR (400 MHz, $CDCl_3$) δ 9.19 (dd, *J* = 2.8, 1.5 Hz, 1H), 8.54 (dt, *J* = 4.8, 1.4 Hz, 1H), 7.96 (dd, *J* = 4.9, 2.5 Hz, 1H), 2.73 (s, 3H).

^{13}C NMR (101 MHz, $CDCl_3$) δ 145.3, 137.5, 131.6, 127.6, 118.2, 10.6.

HRMS (APCI) m/z: calculated for $C_6H_7N_4$ $[M+H]^+$: 135.0665. Found: 135.0666.

[1,2,3]Triazolo[1,5-a]pyridine (16)

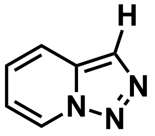

Synthesized from commercially available pyridine-2-carbaldehyde following General Procedure C and was obtained as a light yellow solid.

Yield: 71% (67.6 mg)

Purification: Eluted in 80% EtOAc in Hexanes

^{1}H NMR (400 MHz, $CDCl_3$) δ 8.72 (dq, J = 7.0, 1.1 Hz, 1H), 8.04 (d, J = 1.1 Hz, 1H), 7.71 (dt, J = 8.9, 1.2 Hz, 1H), 7.23 (ddd, J = 8.9, 6.6, 1.0 Hz, 1H), 6.96 (td, J = 6.8, 1.2 Hz, 1H).

^{13}C NMR (101 MHz, $CDCl_3$) δ 133.7, 125.7, 125.2, 118.0, 115.3.

HRMS (APCI) m/z: calculated for $C_6H_6N_3$ $[M+H]^+$: 120.0556. Found: 120.0557.

[1,2,3]triazolo[1,5-a]quinoline (17)[4]

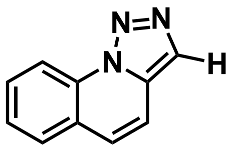

Synthesized from commercially available quinoline-2-carbaldehyde following General Procedure C and was obtained as a white solid.

Yield: 80% (108.3 mg)

Purification: Eluted in 30% EtOAc in Hexanes

^{1}H NMR (400 MHz, $CDCl_3$) δ 8.77 (dd, J = 8.4, 1.2 Hz, 1H), 8.10 (s, 1H), 7.82 (dd, J = 7.9, 1.5 Hz, 1H), 7.74 (ddd, J = 8.6, 7.3, 1.5 Hz, 1H), 7.58 (ddd, J = 8.2, 7.2, 1.2 Hz, 1H), 7.55 – 7.47 (m, 2H).

^{13}C NMR (101 MHz, $CDCl_3$) δ 131.9, 131.8, 130.2, 128.6, 127.7, 127.2, 126.8, 124.0, 116.4, 114.8.

3-Isopropyl-[1,2,3]triazolo[1,5-a]pyridine (18)

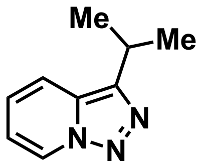

Synthesized from commercially available 2-methyl-1-(pyridin-2-yl)propan-1-one following General Procedure C and was obtained as a yellow oil.

Yield: 90% (116.1 mg)

Purification: Eluted in 30% EtOAc in Hexanes

^{1}H NMR (400 MHz, $CDCl_3$) δ 8.65 (dt, *J* = 7.1, 1.1 Hz, 1H), 7.67 (dt, *J* = 9.0, 1.3 Hz, 1H), 7.13 (ddd, *J* = 9.0, 6.6, 1.0 Hz, 1H), 6.90 (td, *J* = 6.9, 1.3 Hz, 1H), 3.42 (hept, *J* = 7.0 Hz, 1H), 1.46 (d, *J* = 7.0 Hz, 6H).

^{13}C NMR (101 MHz, $CDCl_3$) δ 144.2, 130.5, 125.4, 123.6, 118.0, 115.0, 26.3, 22.6.

HRMS (APCI) m/z: calculated for $C_9H_{12}N_3$ $[M+H]^+$: 162.1026. Found: 162.1026.

Additional Reaction Procedures

Gram Scale Procedure for the Biocatalytic Synthesis of [1, 2, 3]Triazolo[1, 5-a]Pyridines:

A solution of hydrazine hydrate (64-65% in water, 12.8 mmol, 2.0 equiv) was added, to the solution containing 2-benzoylpyridine (6.4 mmol, 1.0 equiv) and ethanol (25 mL). The reaction mixture was refluxed for 18 h. After this time, the reaction mixture was allowed to cool to room temperature and concentrated under reduced pressure, yielding a crude hydrazone mixture that was transferred to a 2 L round-bottom flask containing MeCN (480 mL). Eight enzyme aliquots of VHPO from *Corallina officinalis* (*Co*VBPO, 10 µM, 10 mL) were removed from a -80 °C freezer and allowed to warm to room temperature over 10 min. After thawing the enzyme aliquots, 80 mL solution of *Corallina officinalis* (*Co*VBPO, 10.0 µM) was transferred to a 250 mL round bottom flask and combined with 250 mM solution of aqueous Na_3VO_4 (6.40 mL). This solution was then allowed to sit at room temperature for 30 minutes. The crude hydrazone mixture in the 2 L round-bottom was then combined with Milli-Q H_2O (716 mL), and 500 mM pH 6.5 PIPES (320 mL). The contents of the 250 mL round bottom flask containing *Co*VBPO (0.0125 mol%, 0.5 µM in reaction) and Na_3VO_4 (0.25 equiv) were added to the reaction mixture followed by addition of a 10% stock of H_2O_2 (6.32 mL, 3.0 equiv). The reaction was then left to stir at room temperature at 900 rpm for 8 hr. After this time, the reaction mixture was washed three times with ethyl acetate (3 x 250 mL) to extract the product. The combined organic layers were then washed with brine (100 mL), dried over Na_2SO_4, and concentrated under reduced pressure. The resulting crude sample was purified via flash column chromatography the pure product. The resulting crude sample was purified on a silica gel hand column to obtain the pure product (**3**) in 89% yield, 1.1 g.

Procedure for Visible-Light-Induced Arylation Reaction of [1, 2, 3]Triazolo[1, 5-a]Pyridines:

The following procedure was adapted according to a literature procedure.[5] An oven-dried 20 mL scintillation vial equipped with a magnetic stir bar was charged with 3-phenyl-[1,2,3]triazolo[1,5-a]pyridine (0.2 mmol, 1.0 equiv) in dry benzene (2.0 mL) under an argon atmosphere, followed by the addition of K_2CO_3 (0.6 mmol, 3.0 equiv), phenylboronic acid (0.3 mmol, 1.5 equiv). The reaction mixture was irradiated with a 40 W 390 nm Kessil lamp for 16 h while cooling with a fan, maintaining a distance of approximately 2–3 cm between the lamp and the vial. After completion, the resulting mixture was passed through a pad of Celite and concentrated under reduced pressure. The crude residue was purified by flash column chromatography to afford the pure product (**20**) as a white solid. The characterization data is in agreement with literature precedent.[5]

Yield: 83% (40.7 mg)

Purification: Eluted with 10% EtOAc in Hexanes

^{1}H NMR (400 MHz, $CDCl_3$) δ 8.60 (ddd, *J* = 4.9, 1.9, 0.9 Hz, 1H), 7.61 (td, *J* = 7.7, 1.9 Hz, 1H), 7.34 – 7.27 (m, 4H), 7.25 – 7.20 (m, 2H), 7.19 – 7.11 (m, 5H), 7.09 (dt, *J* = 7.9, 1.1 Hz, 1H), 5.71 (s, 1H).

^{13}C NMR (101 MHz, $CDCl_3$) δ 163.3, 149.7, 142.8, 136.6, 129.5, 128.6, 126.7, 123.9, 121.6, 59.5.

Procedure for Visible-Light-Induced Cyclopropanation of [1, 2, 3]Triazolo[1, 5-a]Pyridines:

The following procedure was adapted according to a literature procedure.[5] An oven dried 20 mL scintillation vial containing a stirring bar was charged with 3-phenyl-[1,2,3]triazolo[1,5-a]pyridine (0.2 mmol, 1.0 equiv), in dry benzene (2 mL) under argon atmosphere and styrene (0.6 mmol, 3.0 equiv) were added. The vial was irradiated with 40 W 390 nm Kessil Lamp for 16 h with cooling from a fan. The vial distance from the lamp was about 2-3 cm. The solvent was removed under a reduced pressure. The crude residue was purified via flash column chromatography to afford the pure product (**21**) as a colorless oil. The characterization data is in agreement with literature precedent.[5]

Yield: 78%, dr 1:0.9, (42.3 mg)

Purification: Eluted with 10% EtOAc in Hexanes

^{1}H NMR (400 MHz, $CDCl_3$) δ 8.63 – 8.55 (m, 1H), 8.46 – 8.37 (m, 1H), 7.50 – 7.45 (m, 2H), 7.40 (td, *J* = 7.7, 1.9 Hz, 1H), 7.37 – 7.27 (m, 3H), 7.25 – 7.16 (m, 4H), 7.15 – 7.01 (m, 9H), 7.02 – 6.96 (m, 2H), 6.93 (dd, *J* = 7.5, 4.2 Hz, 2H), 6.83 (dd, *J* = 7.8, 1.8 Hz, 2H), 6.76 (dd, *J* = 8.1, 1.1 Hz, 1H), 3.33 (dd, *J* = 9.0, 6.9 Hz, 1H), 3.01 (dd, *J* = 9.0, 6.8 Hz, 1H), 2.54 (dd, *J* = 6.8, 5.3 Hz, 1H), 2.25 (dd, *J* = 9.0, 4.5 Hz, 1H), 2.00 (dd, *J* = 6.9, 4.6 Hz, 1H), 1.75 (dd, *J* = 9.0, 5.3 Hz, 1H).

^{13}C NMR (101 MHz, $CDCl_3$) δ 164.0, 159.5, 149.1, 148.7, 145.4, 139.0, 138.4, 138.3, 135.8, 135.7, 132.6, 128.8, 128.6, 128.3, 128.2, 128.1, 127.8, 127.6, 126.9, 126.6, 126.1, 125.8, 125.6, 122.0, 121.1, 120.4, 41.1, 40.4, 34.3, 32.1, 23.1, 19.1.

Procedure for Lewis Acid-Catalyzed Denitrogenative Transannulation of [1, 2, 3]Triazolo[1, 5-a]Pyridines with Benzonitrile:

The following procedure was adapted according to a literature procedure.[6] A reaction vial equipped with a magnetic stir bar was charged with 1,2-dichloroethane (0.25 mL) and boron trifluoride diethyl etherate (25 mol%). To this mixture, 3-phenyl-[1,2,3]triazolo[1,5-a]pyridine (39.0 mg, 0.20 mmol, 1.0 equiv) and benzonitrile (25.7 mg, 0.24 mmol, 1.2 equiv) were added, followed by addition of 1,2-dichlorobenzene (0.20 mL). The reaction mixture was stirred and heated at 120 °C for 16 h. After completion, the mixture was cooled to room temperature and diluted with water (30 mL), then extracted with ethyl acetate (3 x 25 mL). The combined organic layers were washed with brine (25 mL), dried over anhydrous Na_2SO_4, filtered, and concentrated under reduced pressure. The crude residue was purified by flash column chromatography to afford the pure product (**22**) as a white solid. The characterization data is in agreement with literature precedent.[6]

Yield: 90% (48.7 mg)

Purification: Eluted with 5% EtOAc in Hexanes

^{1}H NMR (400 MHz, $CDCl_3$) δ 8.25 (dt, *J* = 7.2, 1.1 Hz, 1H), 7.97 – 7.92 (m, 2H), 7.85 (dt, *J* = 8.0, 1.1 Hz, 3H), 7.54 (dd, *J* = 8.3, 6.8 Hz, 2H), 7.50 – 7.43 (m, 3H), 7.33 – 7.28 (m, 1H), 6.79 (ddd, *J* = 9.3, 6.3, 1.0 Hz, 1H), 6.58 (ddd, *J* = 7.4, 6.3, 1.2 Hz, 1H).

^{13}C NMR (101 MHz, $CDCl_3$) δ 138.3, 135.1, 132.1, 130.3, 129.2, 129.0, 128.5, 127.8, 127.0, 126.7, 121.9, 119.8, 119.3, 113.4.

Procedure for Pd-catalyzed C-6 Functionalization of Pyridines:

The following procedure was adapted according to a literature procedure.[7] A mixture of 3-phenyl-[1,2,3]triazolo[1,5-a]pyridine (97.6 mg, 0.50 mmol, 1.0 equiv), iodobenzene (204.0 mg, 1.0 mmol, 2.0 equiv), palladium(II) acetate (11.2 mg, 10 mol%), and silver carbonate (276.0 mg, 1.0 mmol, 2.0 euiv) was placed in a reaction vial equipped with a magnetic stir bar. Dry toluene (2.0 mL) was then added, and the suspension was heated at 120 °C under stirring for 16. Upon completion, the reaction mixture was cooled to room temperature and diluted with water (30 mL), followed by extraction with ethyl acetate (3 x 25 mL). The combined organic layers were washed with brine (25 mL), dried over anhydrous Na_2SO_4, filtered, and concentrated under reduced pressure. The crude product was purified by flash column chromatography to yield the pure compound (**23**) as a slight yellow liquid. The characterization data is in agreement with literature precedent.[7]

Yield: 72% (93.4 mg)

Purification: Eluted with 10% EtOAc in Hexanes

^{1}H NMR (400 MHz, $CDCl_3$) δ 8.22 (dt, *J* = 7.1, 1.4 Hz, 2H), 8.08 – 7.92 (m, 5H), 7.65 – 7.60 (m, 1H), 7.54 – 7.41 (m, 5H).

^{13}C NMR (101 MHz, $CDCl_3$) δ 193.8, 156.0, 154.9, 138.5, 138.0, 136.5, 133.0, 131.5, 129.6, 129.0, 128.2, 127.1, 123.0, 122.6.

Optimization Data

All optimization reactions for the synthesis of 3-methyl-[1,2,3]triazolo[1,5-a]pyridine (**2**) were performed using General Procedure B. The only variable changed is the one indicated in the Figures below. *(Note: 100 µL of a 8 mg/mL solution of 1,3,5-trimethoxybenzene was added as an internal standard for yield confirmation, where applicable).*

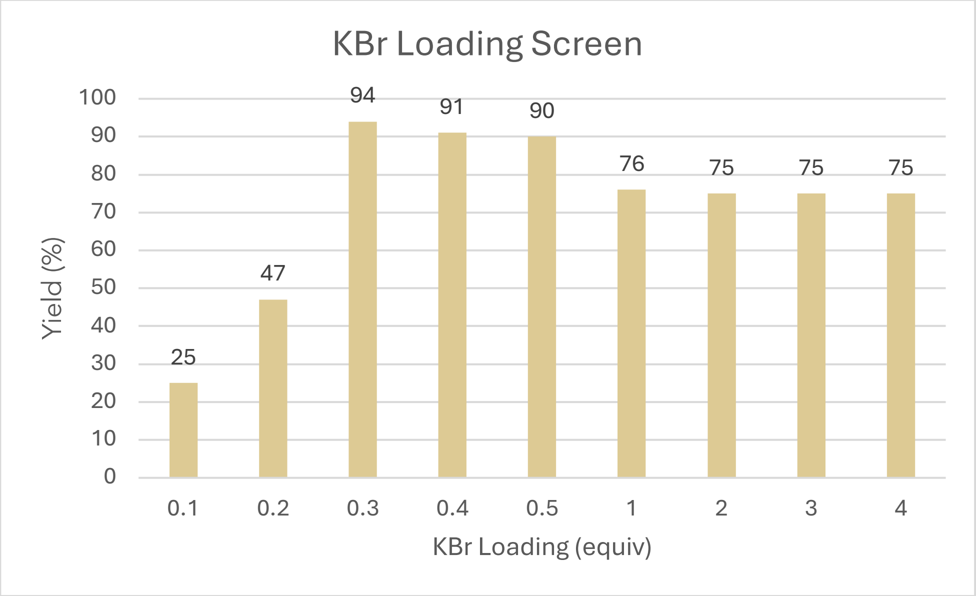

Figure S1. Potassium Bromide (KBr) Loading Screen

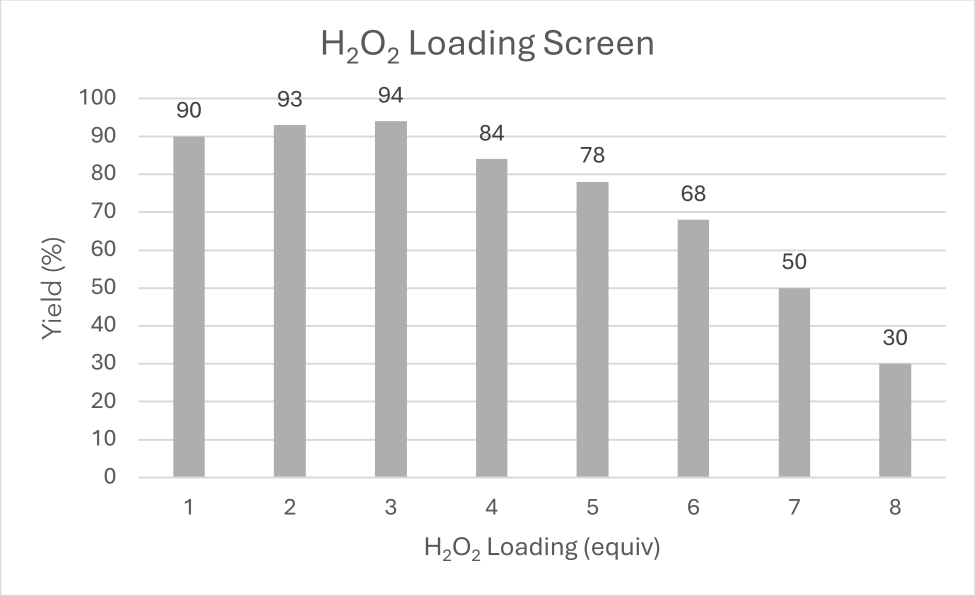

Figure S2. Hydrogen Peroxide (H_2O_2) Loading Screen

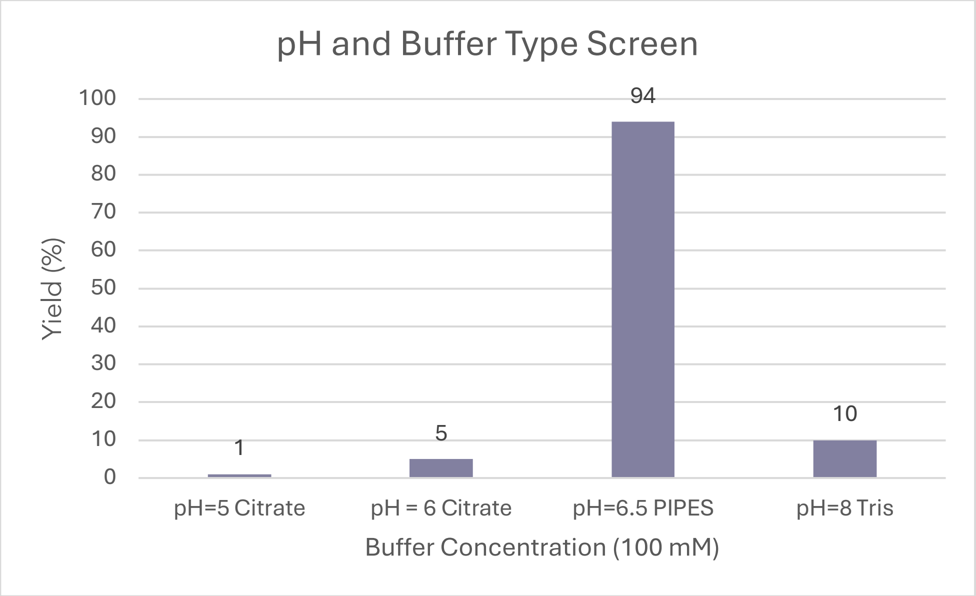

Figure S3. pH and Buffer Type Screen

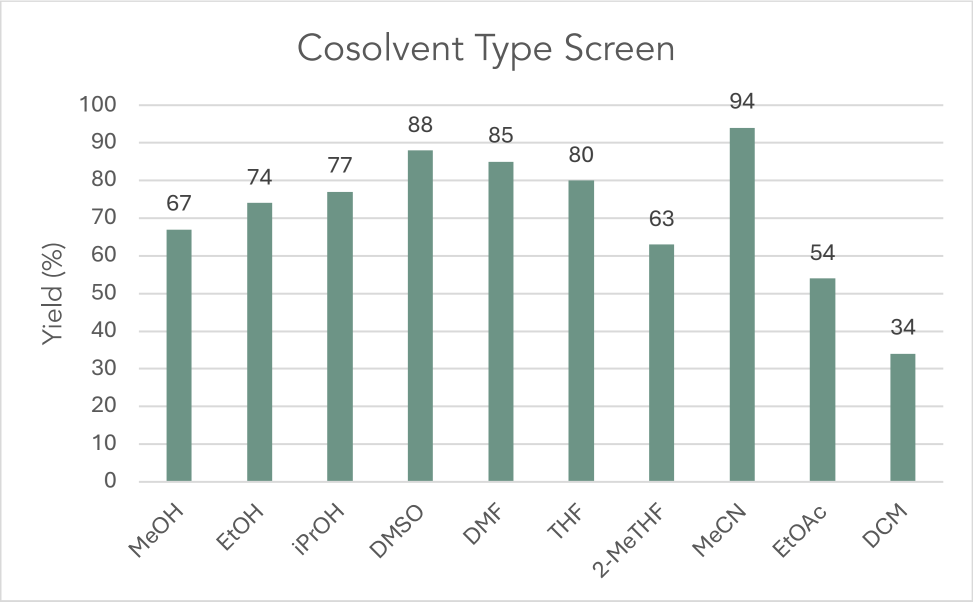

Figure S4. Cosolvent Type Screen

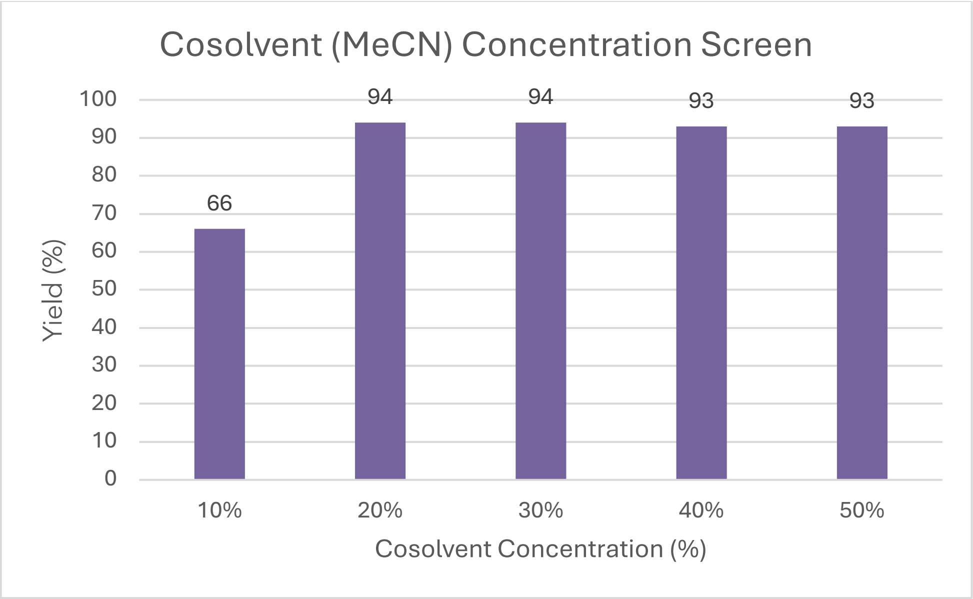

Figure S5. Cosolvent (MeCN) Loading Screen

References:

(1) Wells, C. E.; Ramos, L. P. T.; Harstad, L. J.; Hessefort, L. Z.; Lee, H. J.; Sharma, M.; Biegasiewicz, K.F. Decarboxylative Bromooxidation of Indoles by a Vanadium Haloperoxidase. *ACS Catal.* **2023**, *13* (7), 4622-4628.

(2) Liu, Q.; Wang, C.; Zhou, H.; Wang, B.; Lv, J.; Cao, L.; Fu, Y. Iridium-Catalyzed Highly Enantioselective Transfer Hydrogenation of Aryl *N*-Heteroaryl Ketones with *N*-Oxide as a Removable *ortho*-Substituent. *Org. Lett.* **2018**, *20* (4), 971–974.

(3) Song, S.; Li, Z.; Wang, L.; Zeng, T.; Hu, Q.; Zhu, J. Photoredox and NHC Enabled Deoxygenative Alcohol Homologation via Formal 1,2-Addition. *Org. Lett.* **2024**, *26* (1), 264–268.

(4) Hirayama, T.; Ueda, S.; Okada, T.; Tsurue, N.; Okuda, K.; Nagasawa, H. Facile One-Pot Synthesis of [1, 2, 3]Triazolo[1, 5-*a*]Pyridines from 2-Acylpyridines by Copper(II)-Catalyzed Oxidative N-N Bond Formation.*Chem. Eur. J.* **2014**, *20* (14), 4156–4162.

(5) Zhang, Z.; Yadagiri, D.; Gevorgyan, V. Light-induced metal-free transformations of unactivated pyridotriazoles. *Chem. Sci.* **2019**, *10* (36), 8399–8404.

(6) Joshi, A.; Mohan, D. C.; Adimurthy, S. Copper-Catalyzed Denitrogenative Transannulation Reaction of Pyridotriazoles: Synthesis of Imidazo[1,5-*a*]pyridines with Amines and Amino Acids. *J. Org. Chem.* **2016**, *81* (19), 9461–9469.

(7) Joshi, A.; Semwal, R.; Suresh, E.; Adimurthy, S. Pd-Catalyzed regioselective synthesis of 2,6-disubstituted pyridines through denitrogenation of pyridotriazoles and 3,8-diarylation of imidazo[1,2-*a*]pyridines. *Chem. Commun.* **2019**, *55*, 10888-10891.

Spectroscopic Data

2-(1-Hydrazineylideneethyl)pyridine (1)

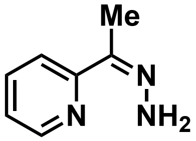

^{1}H-NMR
400 MHz
$CDCl_3$

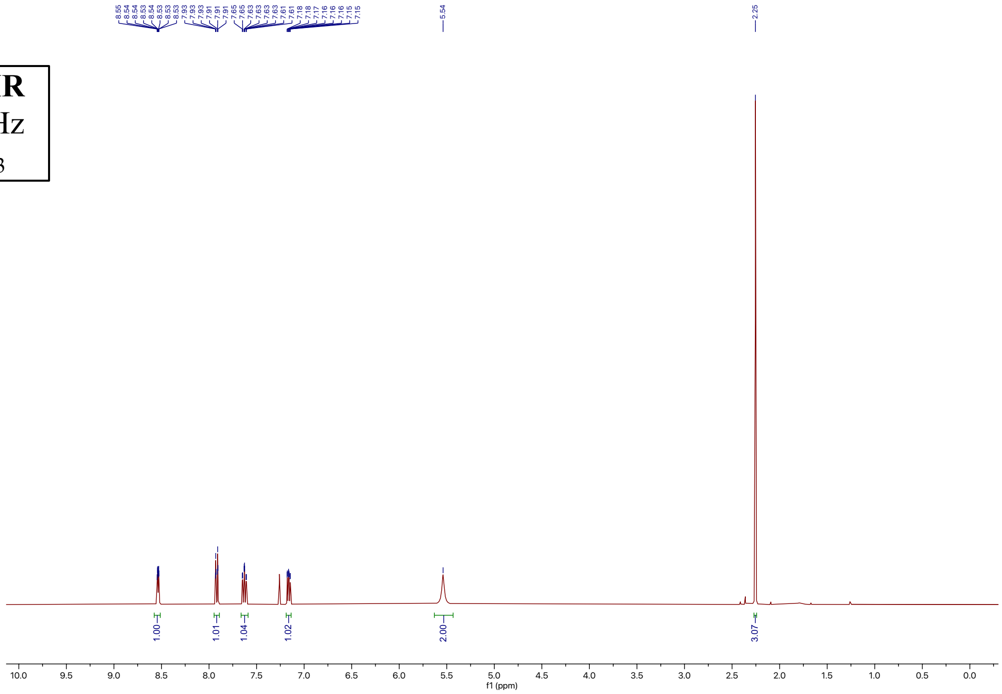

^{13}C-NMR
101 MHz
$CDCl_3$

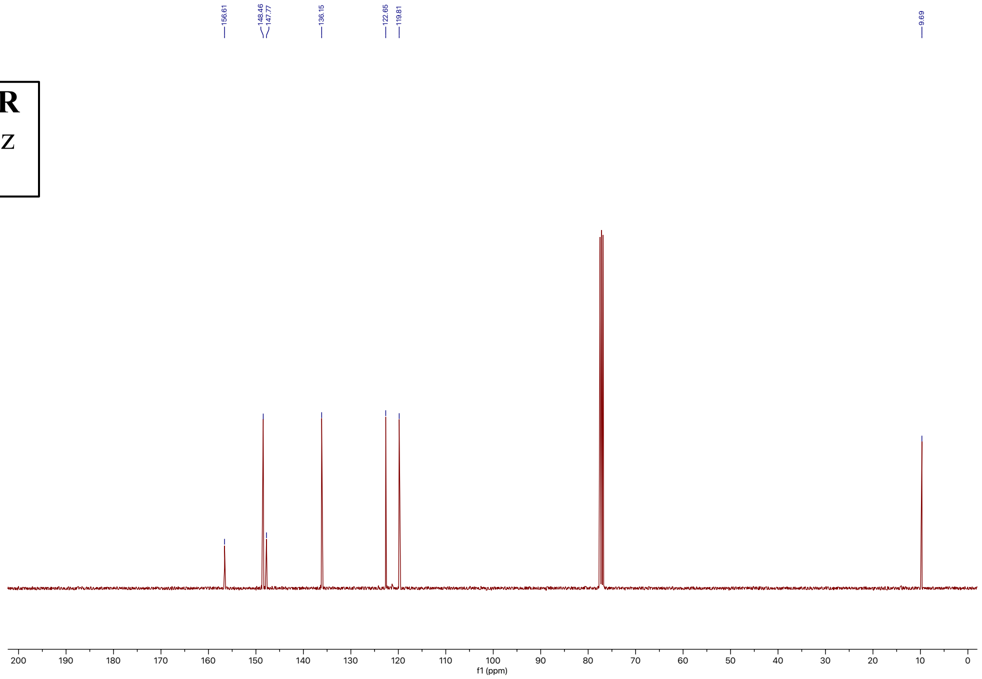

Pyridin-2-yl(p-tolyl)methanone (SM-2)

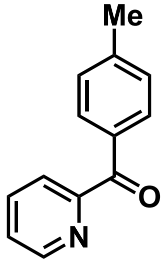

^{1}H-NMR
400 MHz
$CDCl_3$

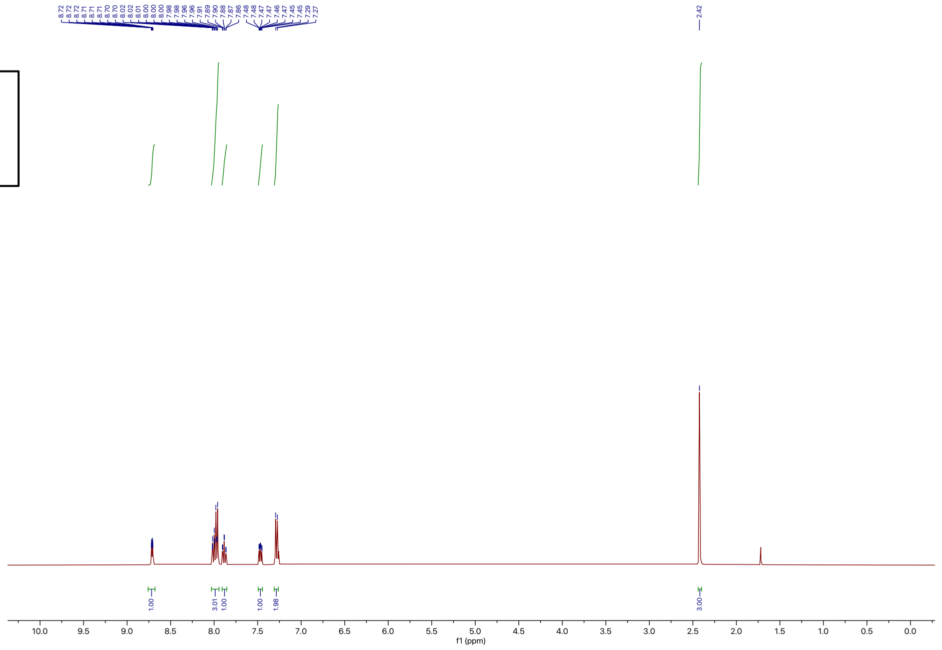

^{13}C-NMR
101 MHz
$CDCl_3$

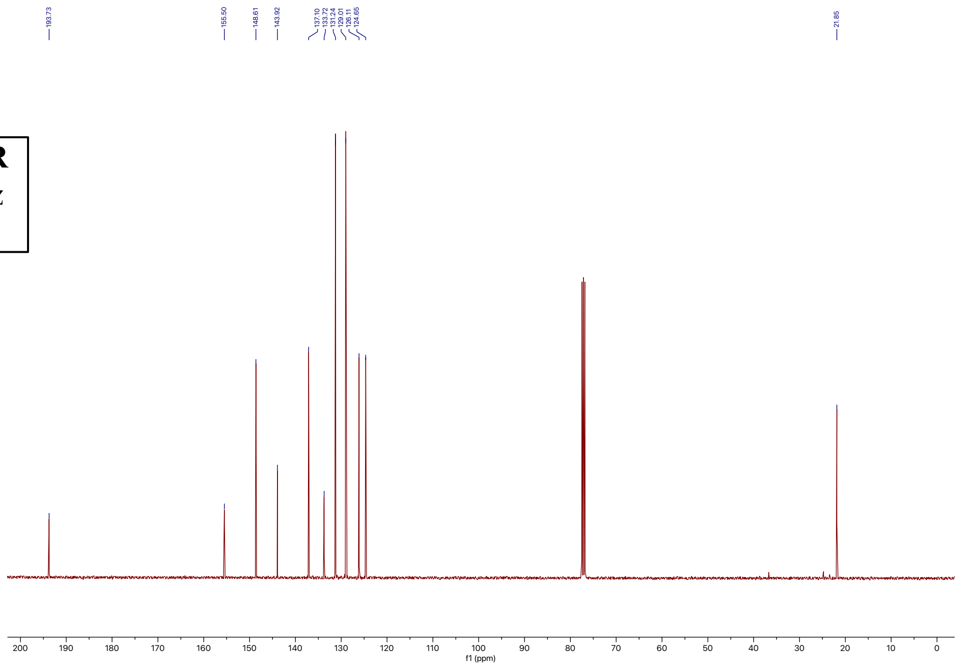

(4-(tert-Butyl)phenyl)(pyridin-2-yl)methanone (SM-3)

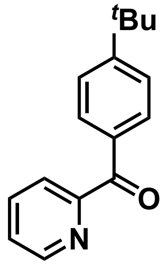

^{1}H-NMR
400 MHz
$CDCl_3$

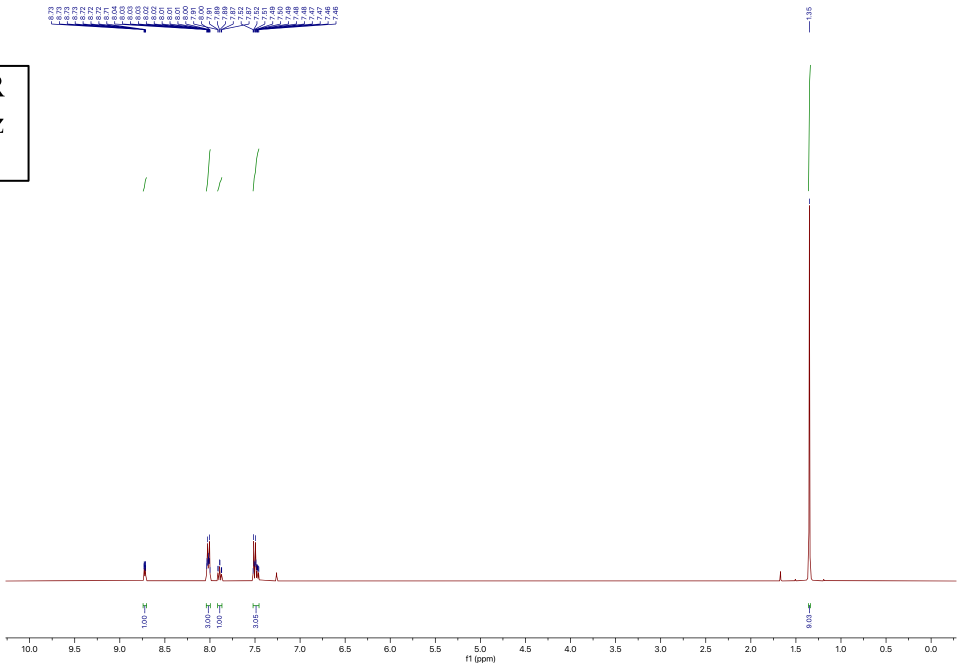

^{13}C-NMR
101 MHz
$CDCl_3$

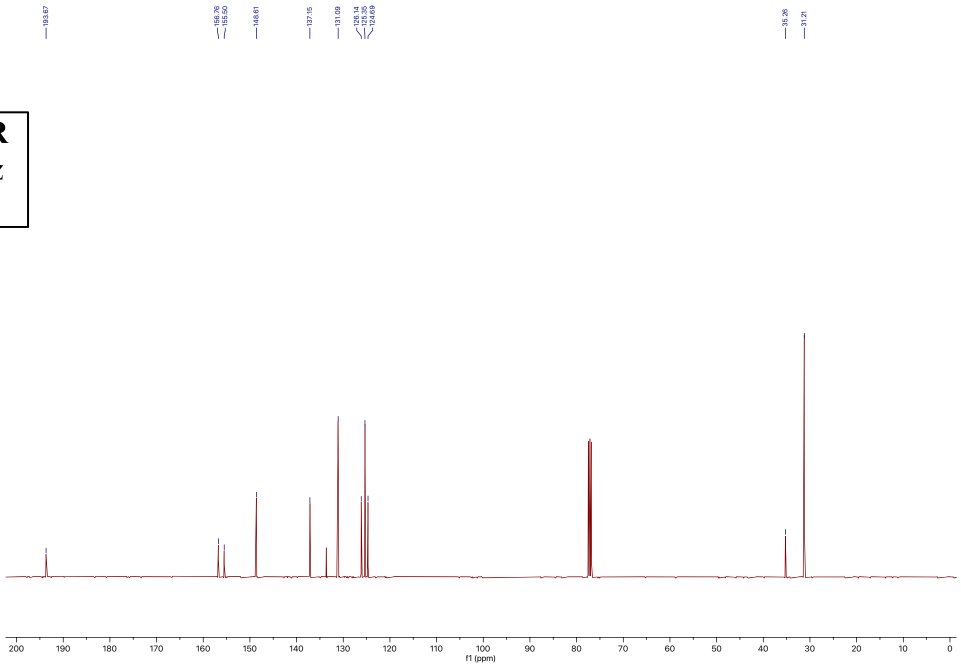

(4-Methoxyphenyl)(pyridin-2-yl)methanone (SM-4)

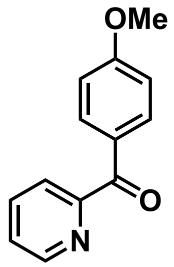

^{1}H-NMR
400 MHz
$CDCl_3$

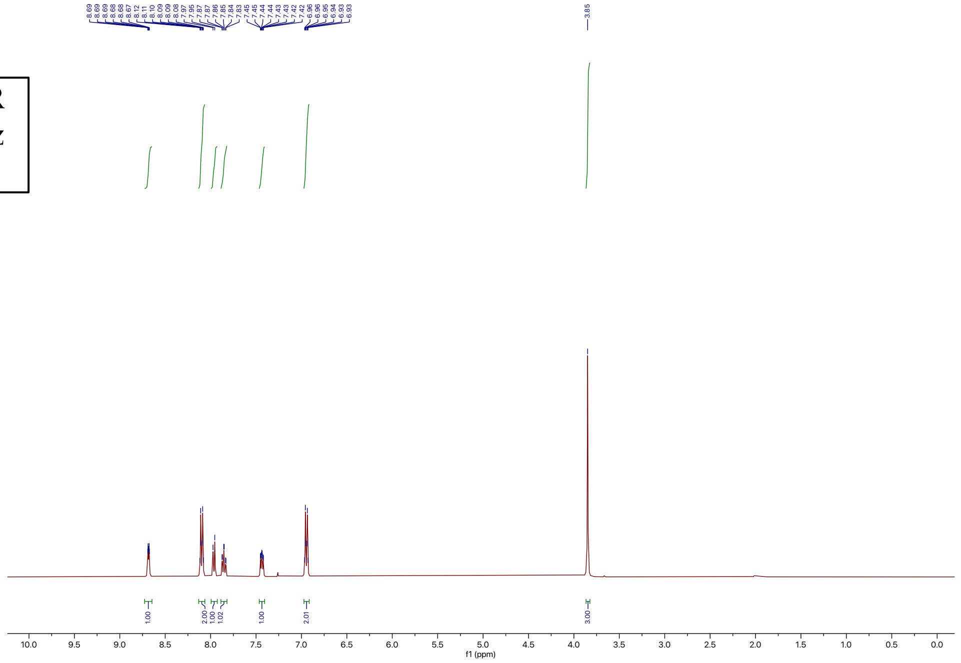

^{13}C-NMR
101 MHz
$CDCl_3$

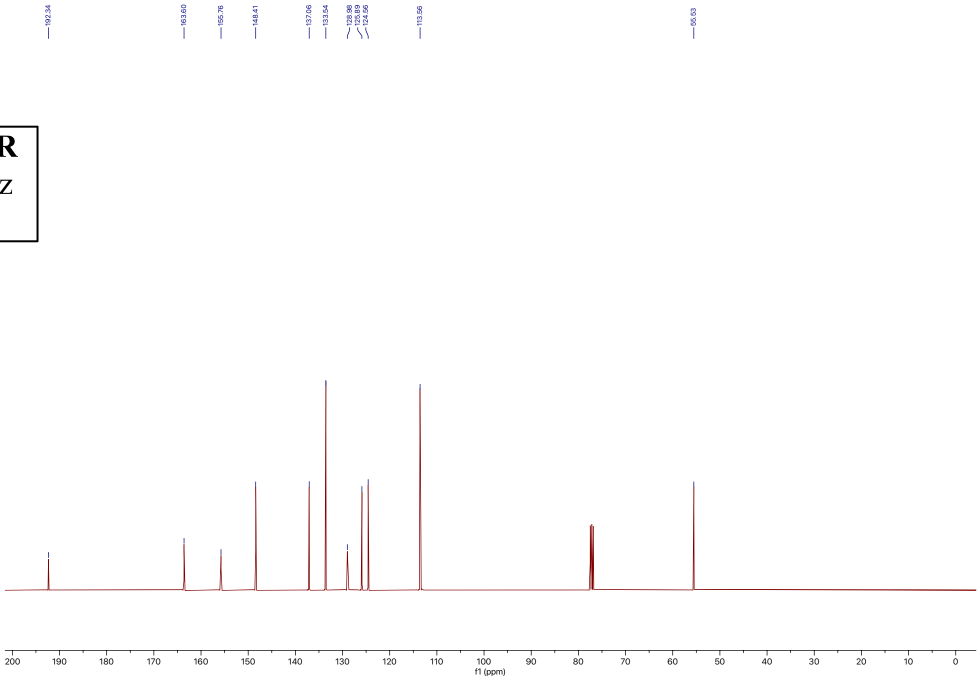

Pyridin-2-yl(4-(trifluoromethyl)phenyl)methanone (SM-5)

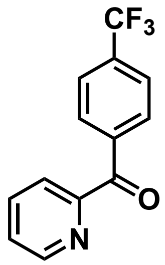

^{1}H-NMR
400 MHz
$CDCl_3$

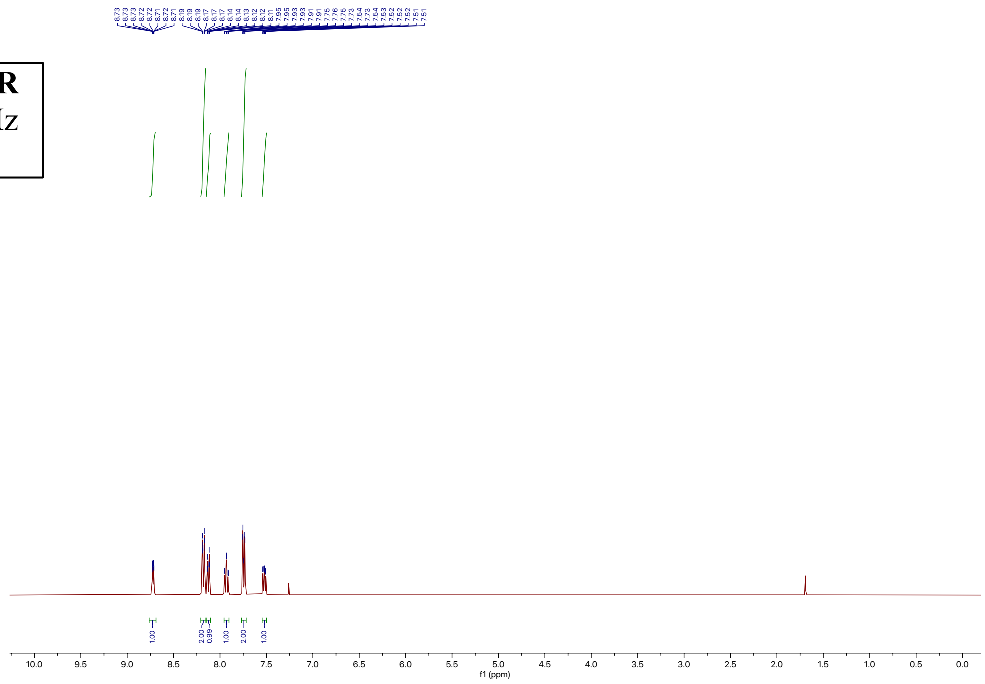

^{13}C-NMR
101 MHz
$CDCl_3$

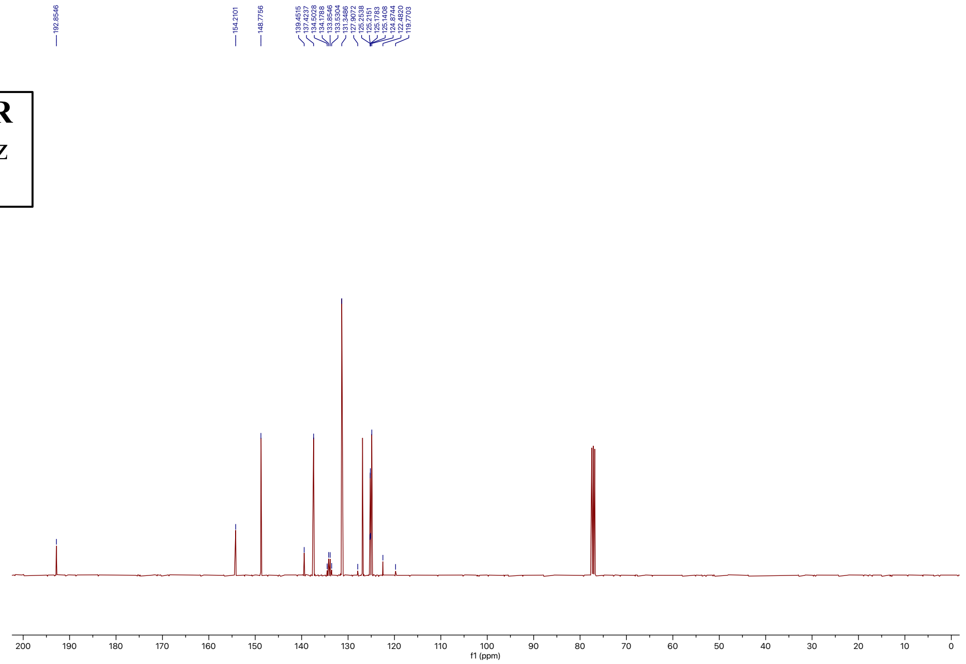

Pyridin-2-yl(m-tolyl)methanone (SM-6)

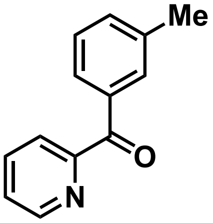

^{1}H-NMR
400 MHz
$CDCl_3$

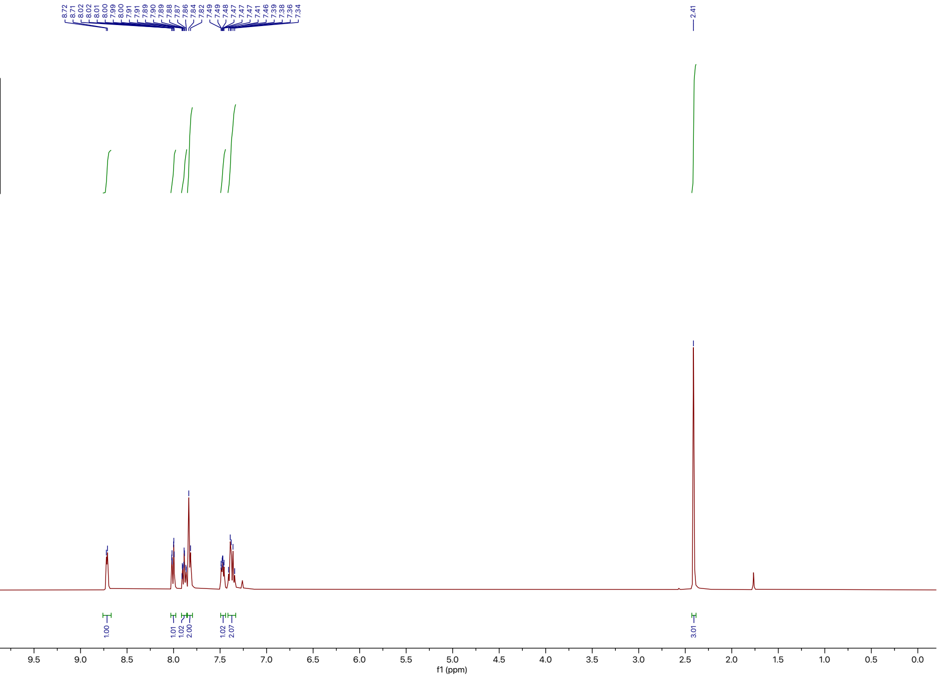

^{13}C-NMR
101 MHz
$CDCl_3$

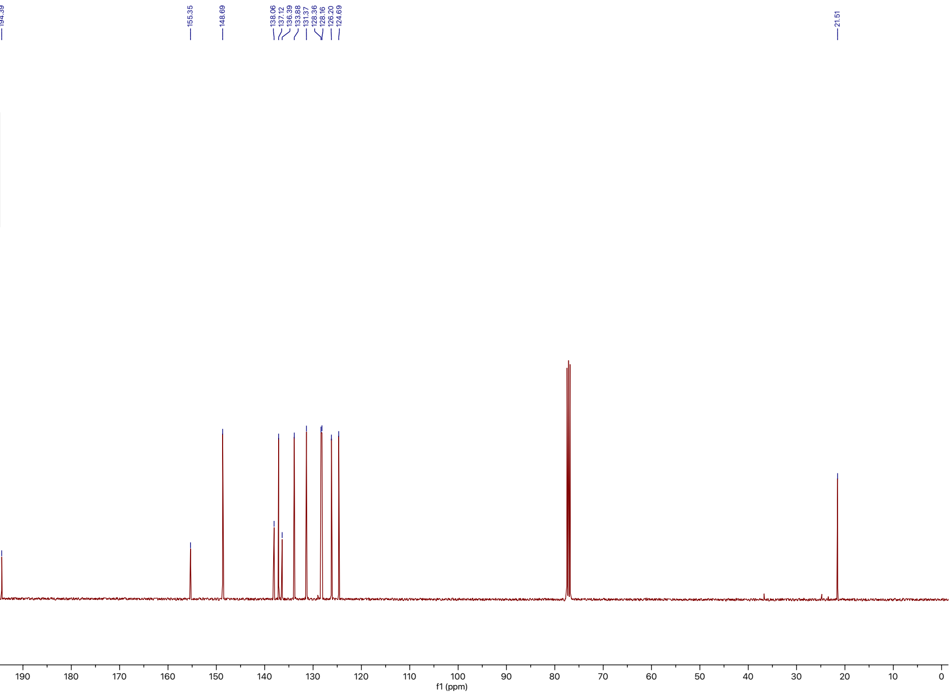

(3-Methoxyphenyl)(pyridin-2-yl)methanone (SM-7)

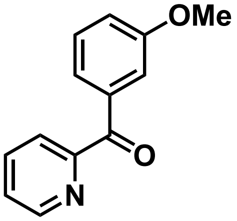

^{1}H-NMR
400 MHz
$CDCl_3$

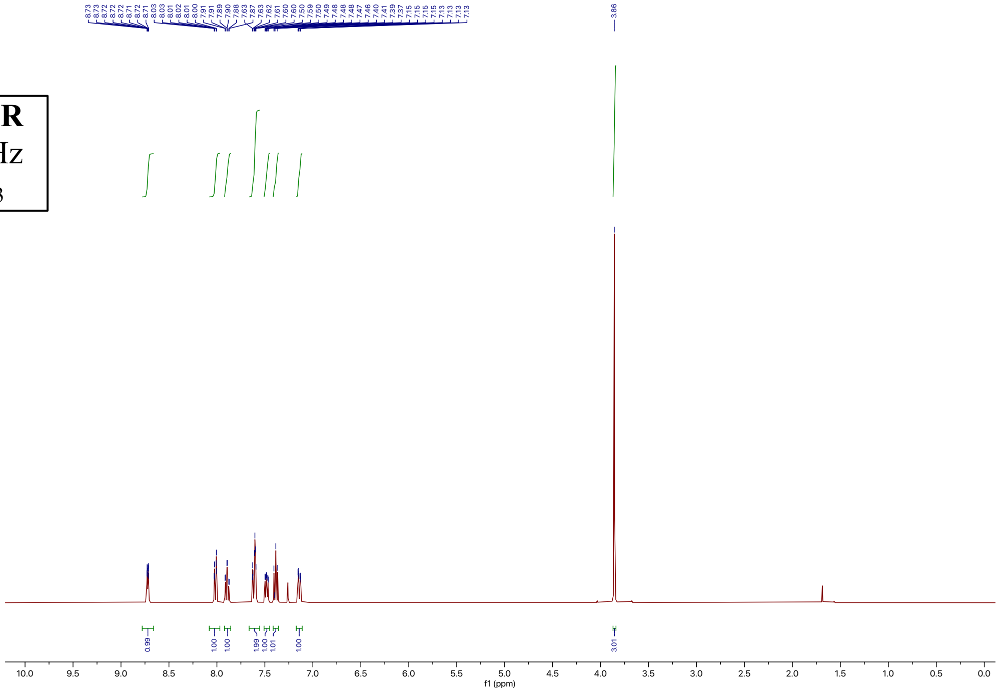

^{13}C-NMR
101 MHz
$CDCl_3$

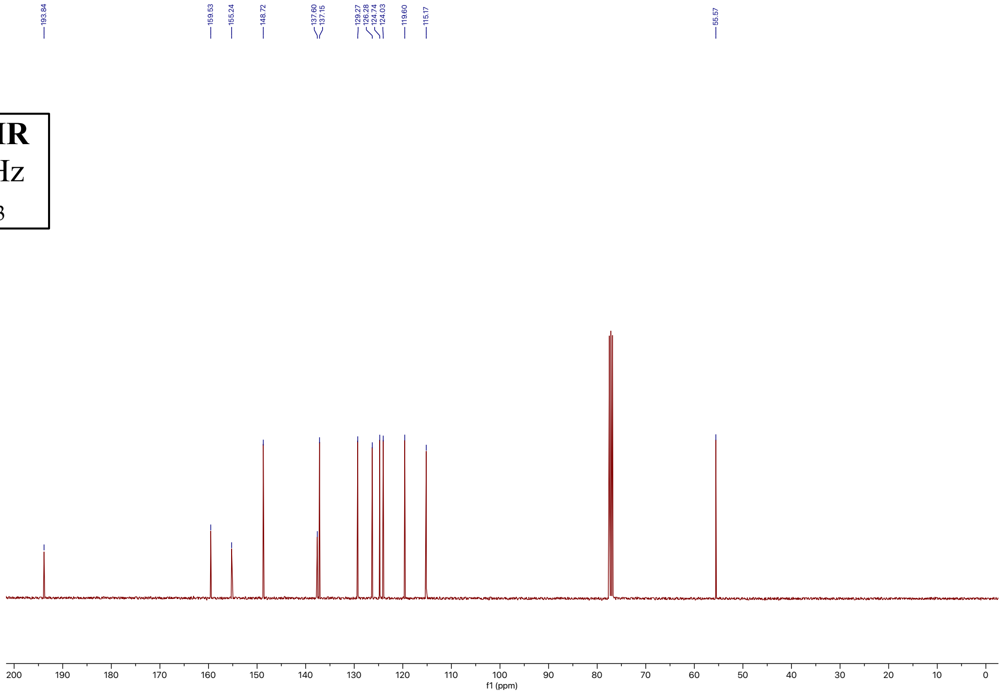

Pyridin-2-yl(o-tolyl)methanone (SM-8)

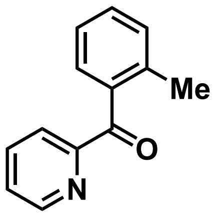

^{1}H-NMR
400 MHz
$CDCl_3$

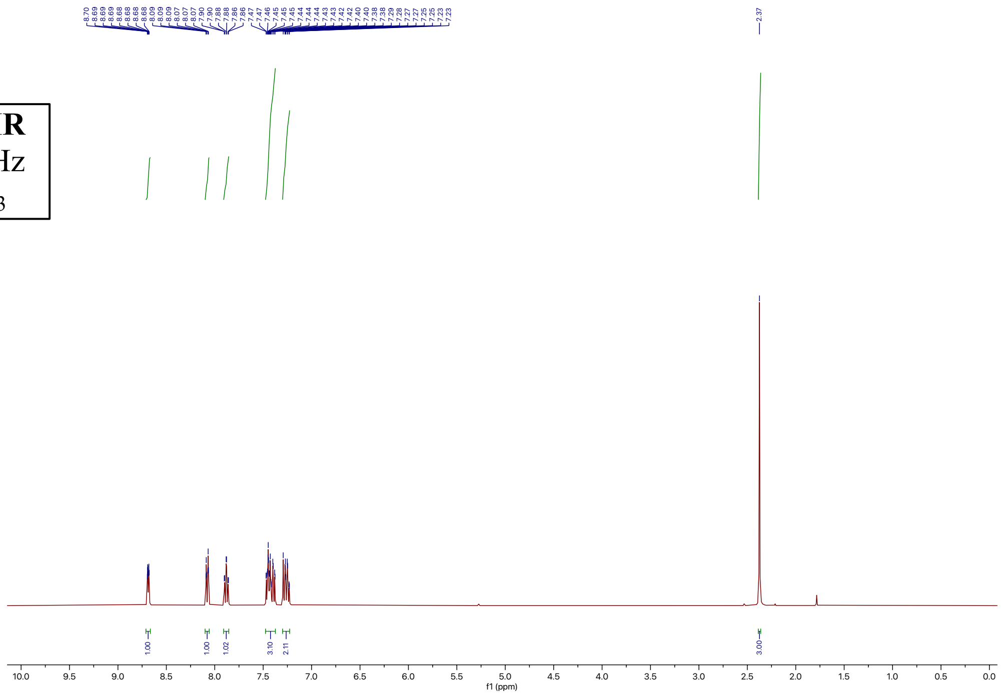

^{13}C-NMR
101 MHz
$CDCl_3$

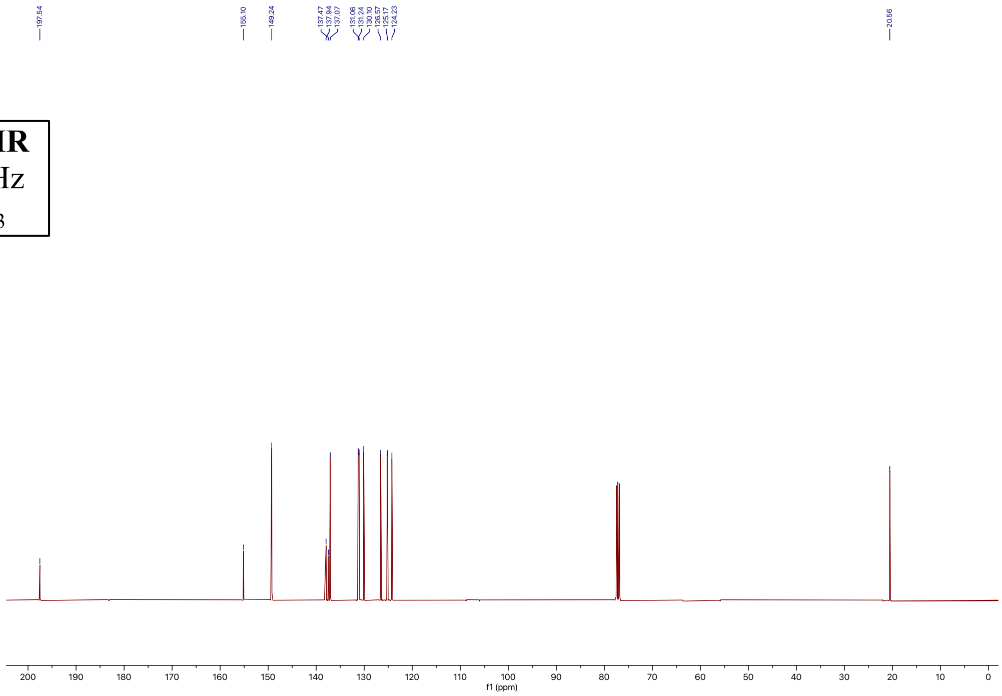

(2-Methoxyphenyl)(pyridin-2-yl)methanone (SM-9)

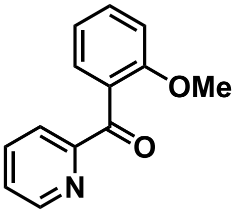

^{1}H-NMR
400 MHz
$CDCl_3$

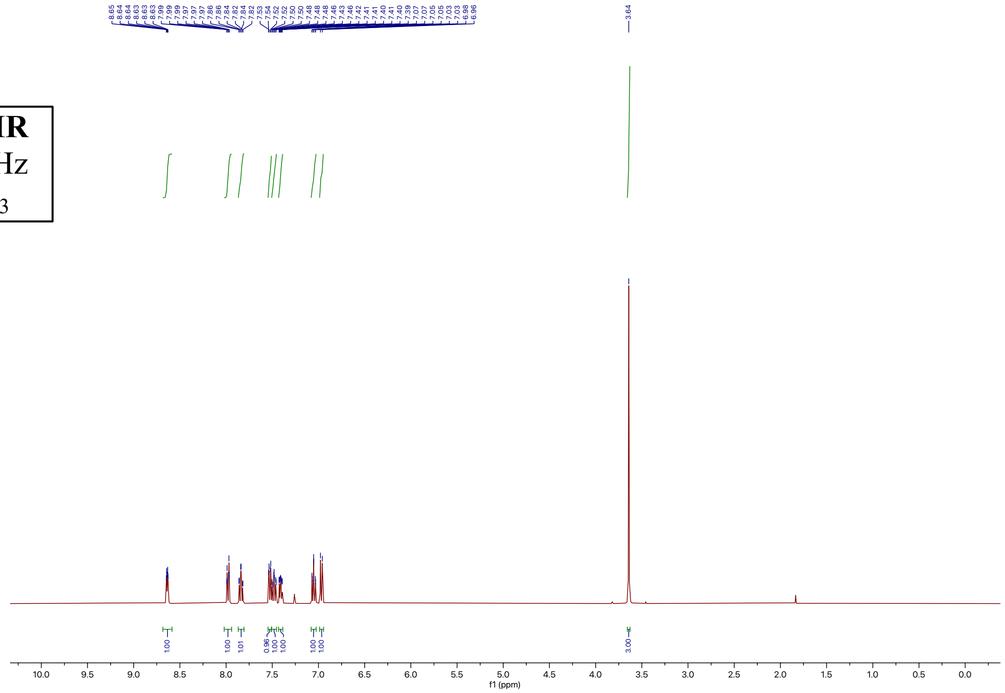

^{13}C-NMR
101 MHz
$CDCl_3$

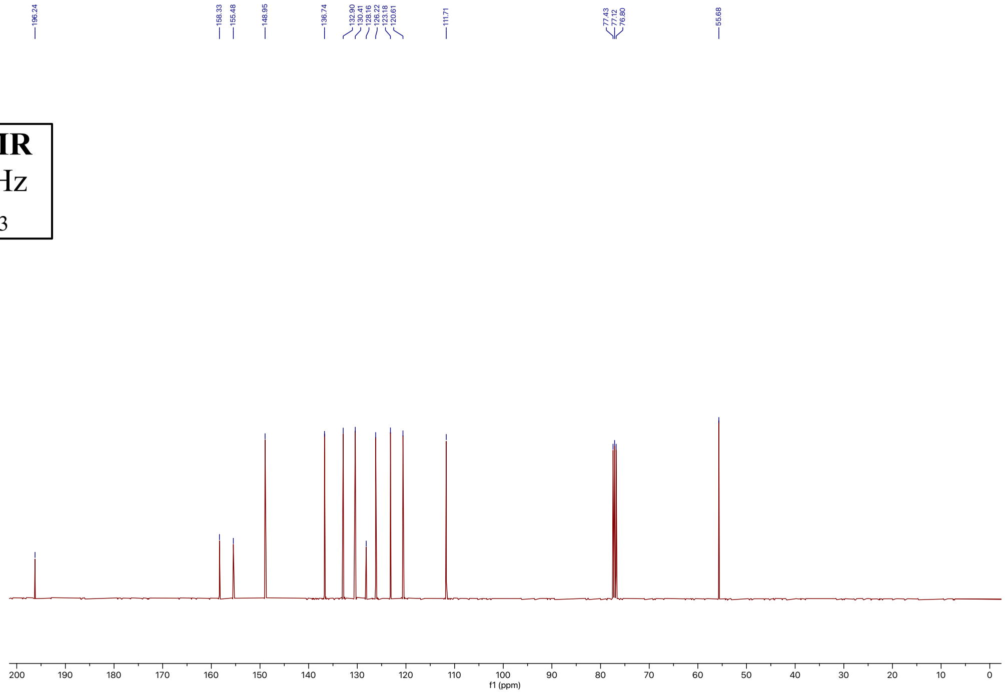

(4-Methylpyridin-2-yl)(phenyl)methanone (SM-10)

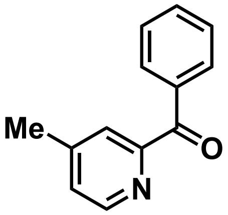

^{1}H-NMR
400 MHz
$CDCl_3$

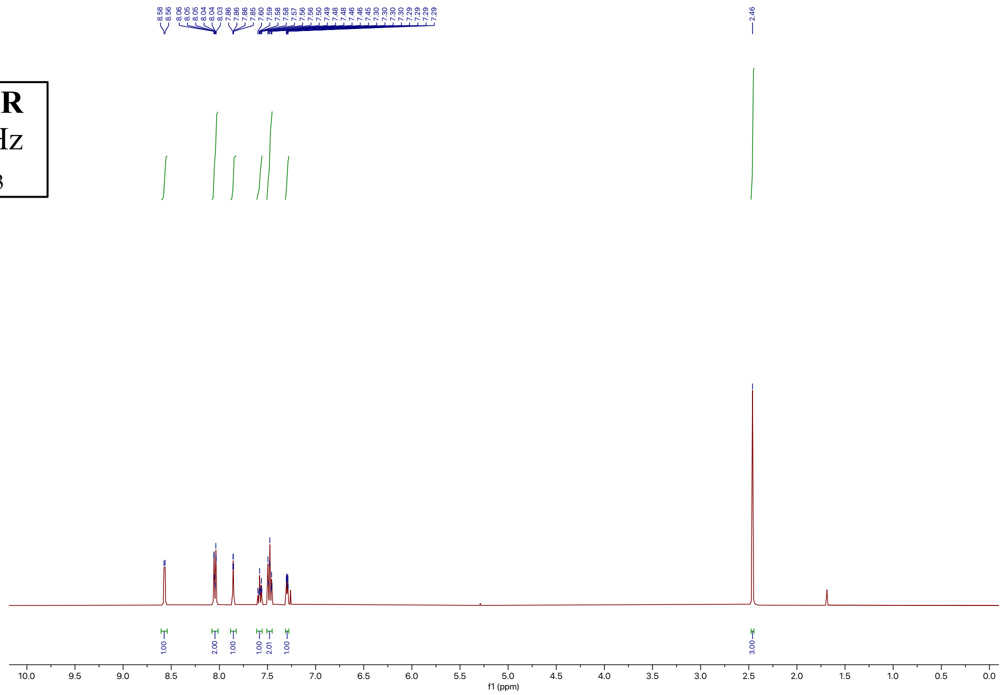

^{13}C-NMR
101 MHz
$CDCl_3$

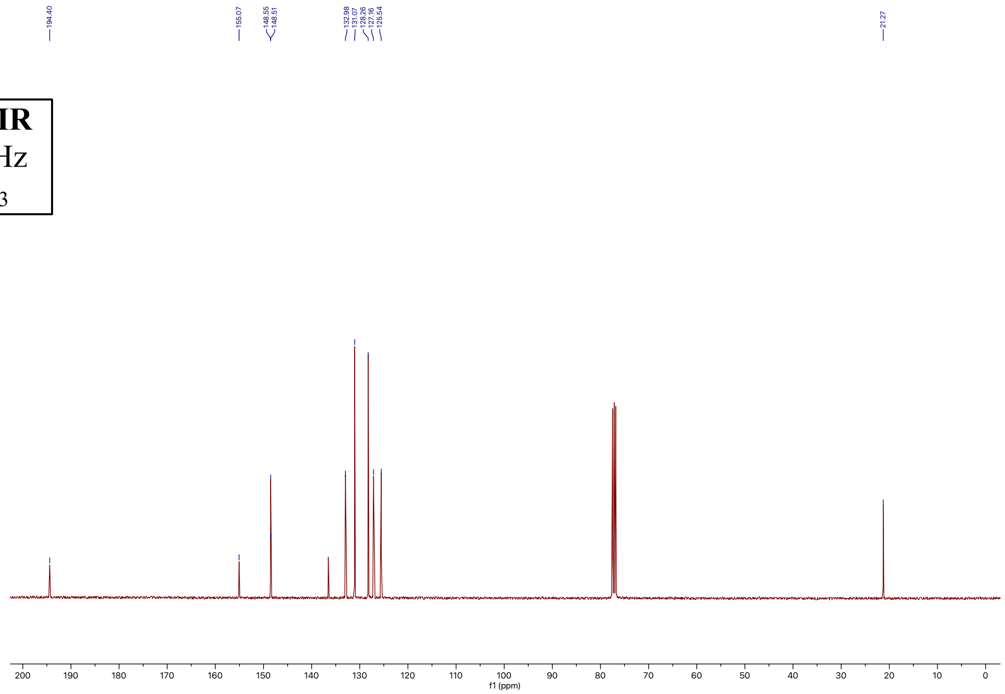

3-Methyl-[1,2,3]triazolo[1,5-a]pyridine (2)

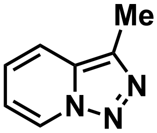

^{1}H-NMR
400 MHz
$CDCl_3$

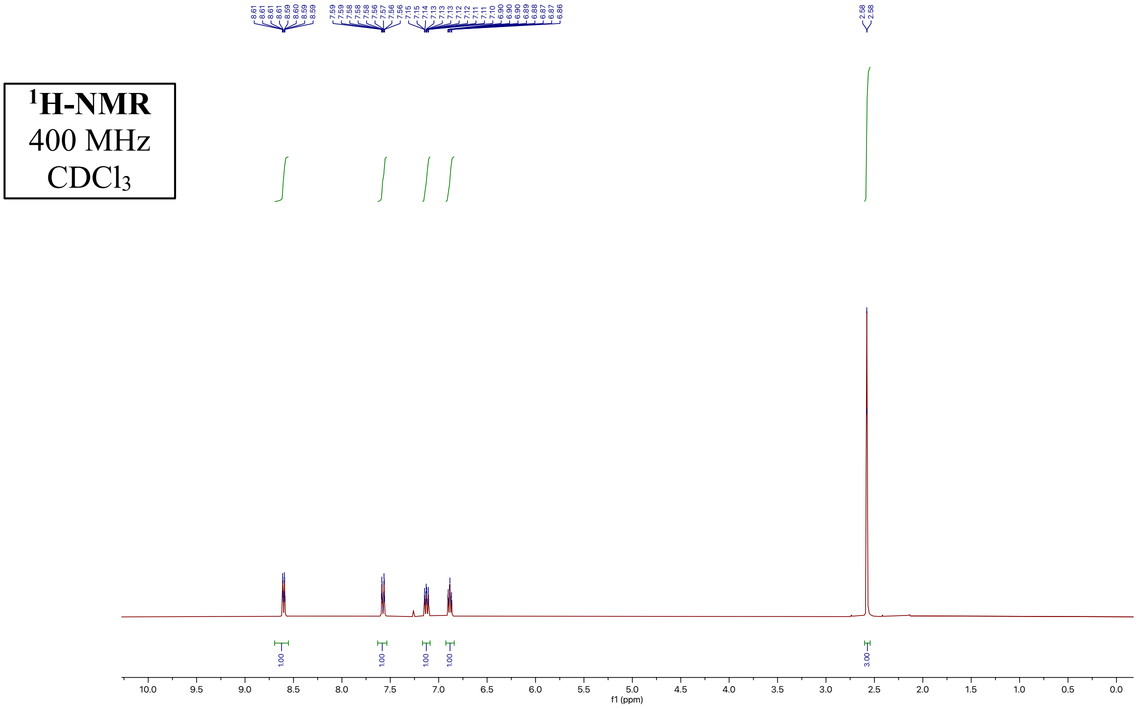

^{13}C-NMR
101 MHz
$CDCl_3$

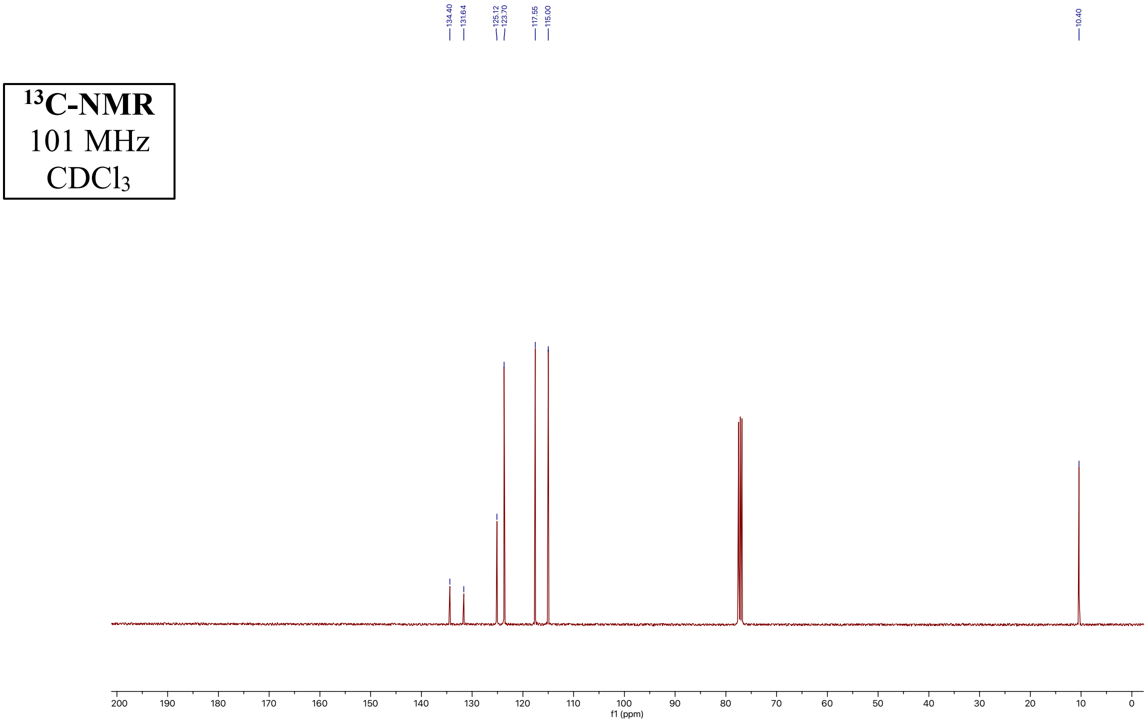

3-Phenyl-[1,2,3]triazolo[1,5-a]pyridine (3)

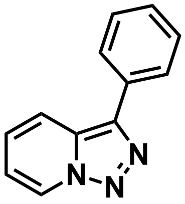

^{1}H-NMR
400 MHz
$CDCl_3$

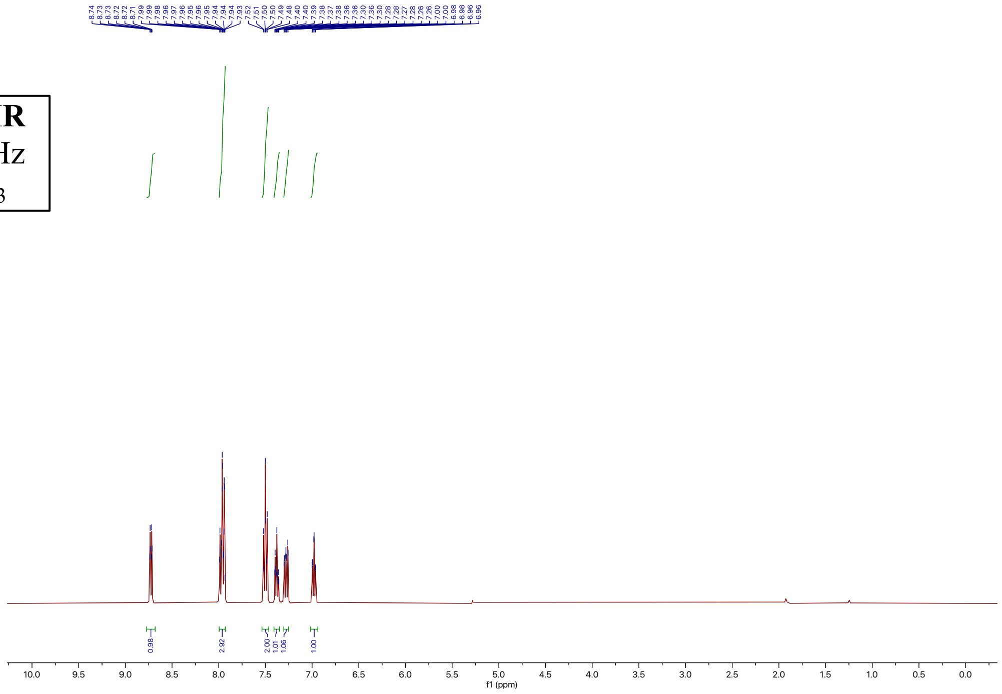

^{13}C-NMR
101 MHz
$CDCl_3$

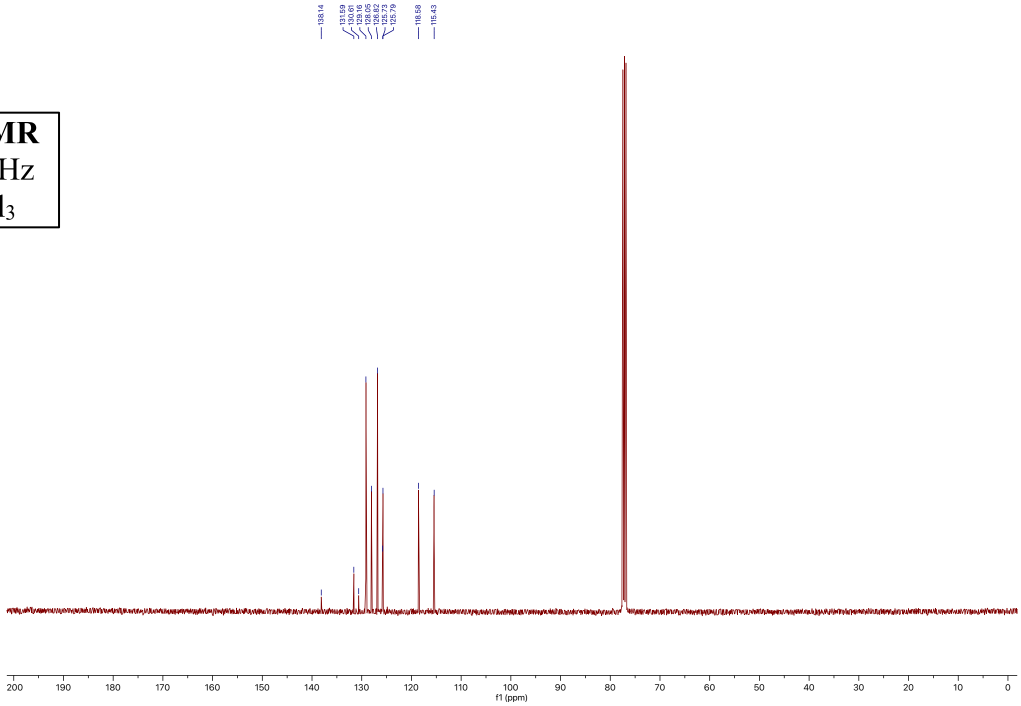

3-(p-Tolyl)-[1,2,3]triazolo[1,5-a]pyridine (4)

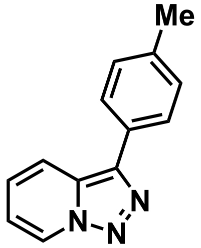

^{1}H-NMR
400 MHz
$CDCl_3$

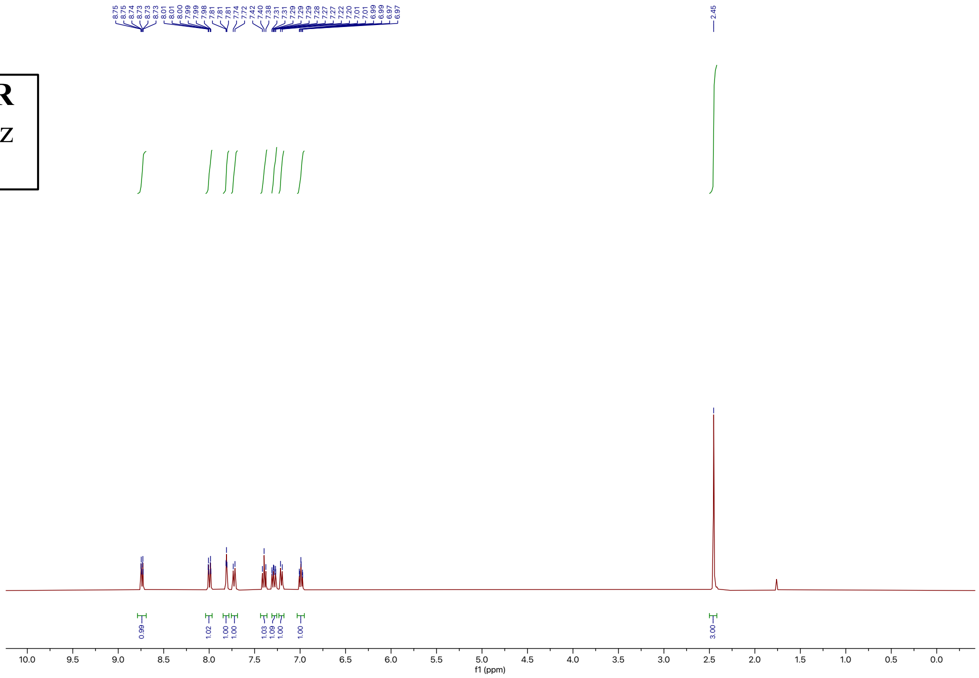

^{13}C-NMR
101 MHz
$CDCl_3$

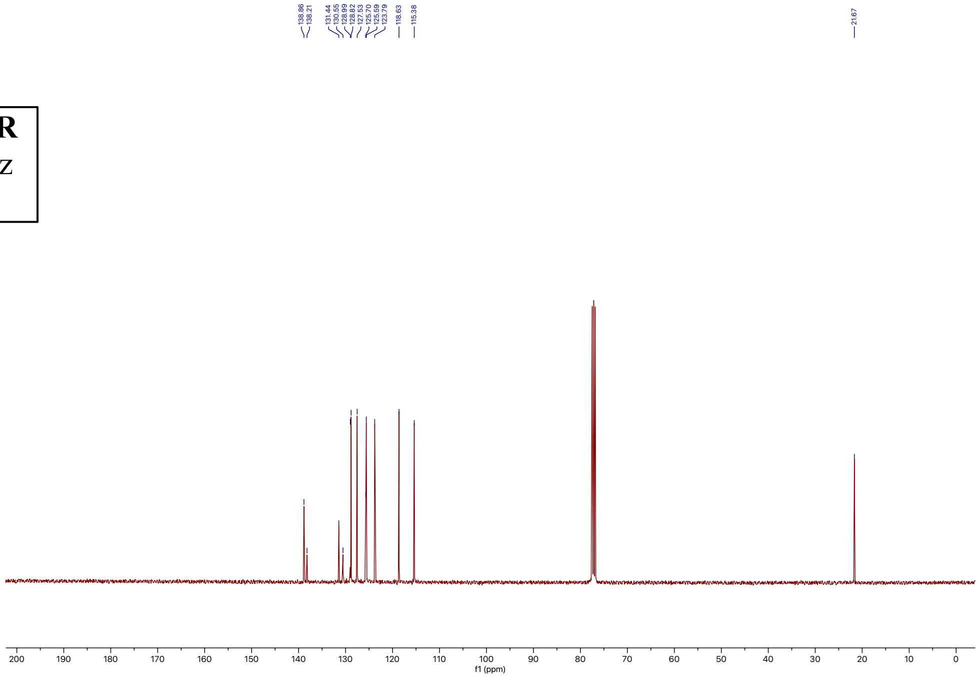

3-(4-(tert-Butyl)phenyl)-[1,2,3]triazolo[1,5-a]pyridine (5)

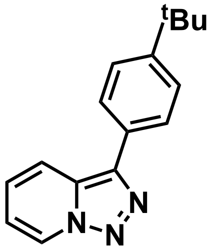

^{1}H-NMR
400 MHz
$CDCl_3$

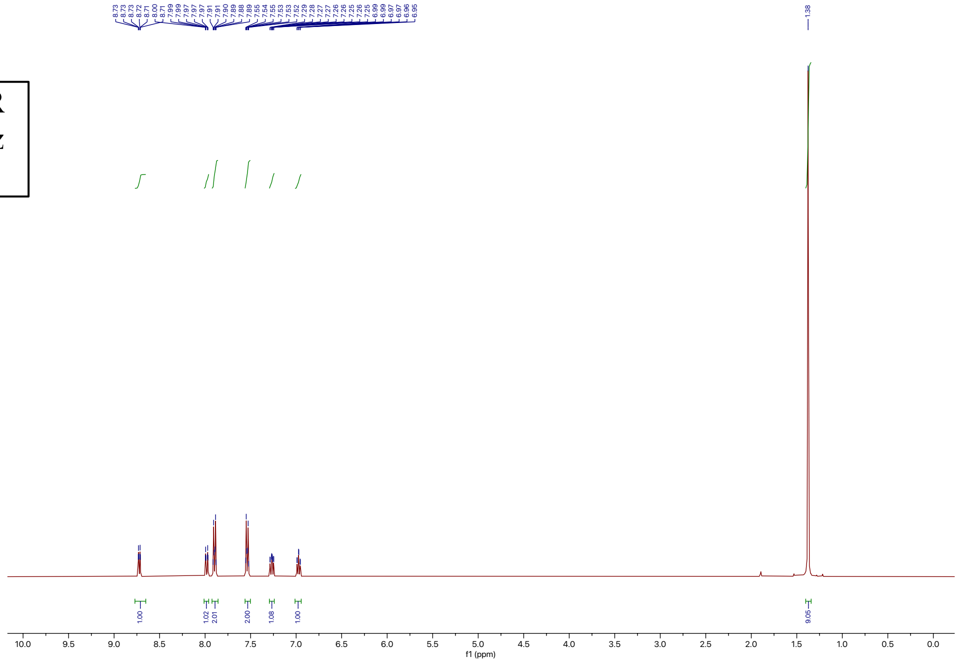

^{13}C-NMR
101 MHz
$CDCl_3$

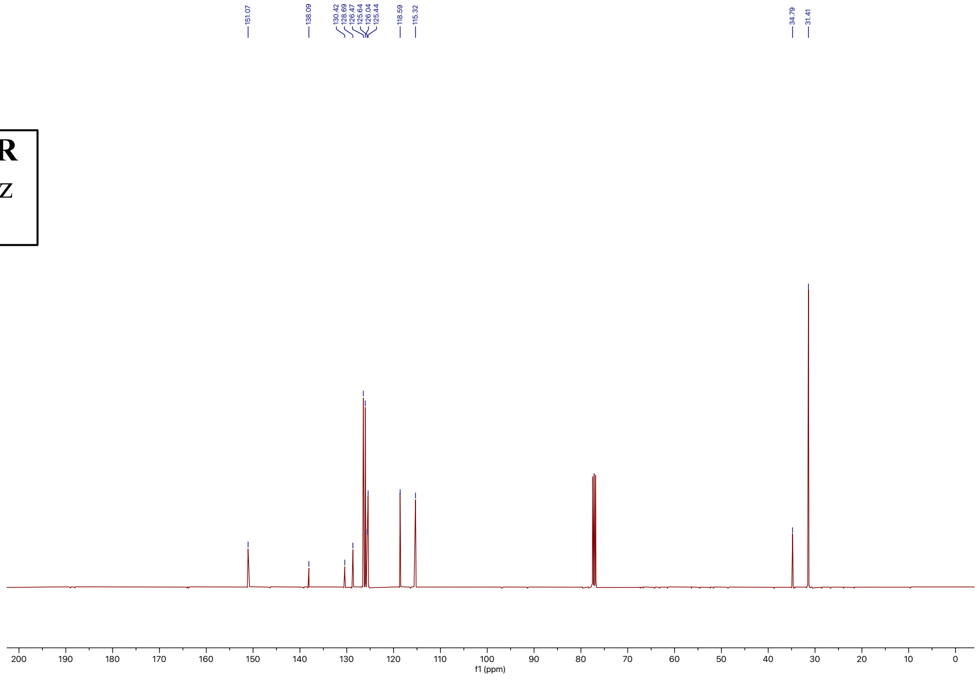

3-(4-Methoxyphenyl)-[1,2,3]triazolo[1,5-a]pyridine (6)

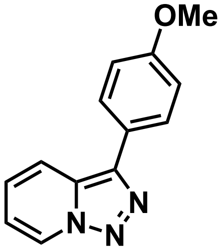

^{1}H-NMR
400 MHz
$CDCl_3$

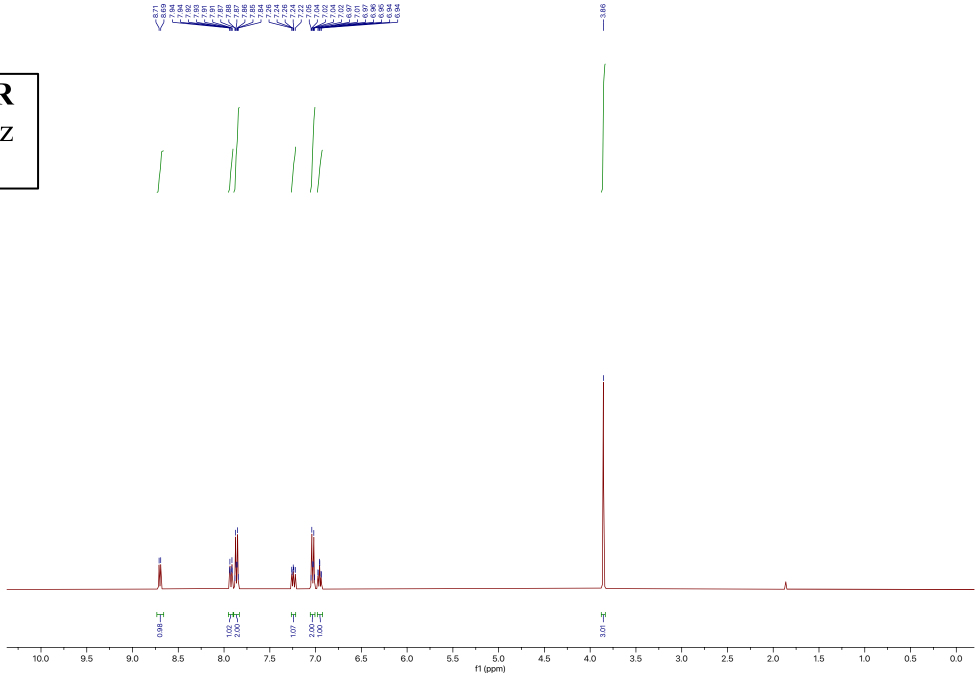

^{13}C-NMR
101 MHz
$CDCl_3$

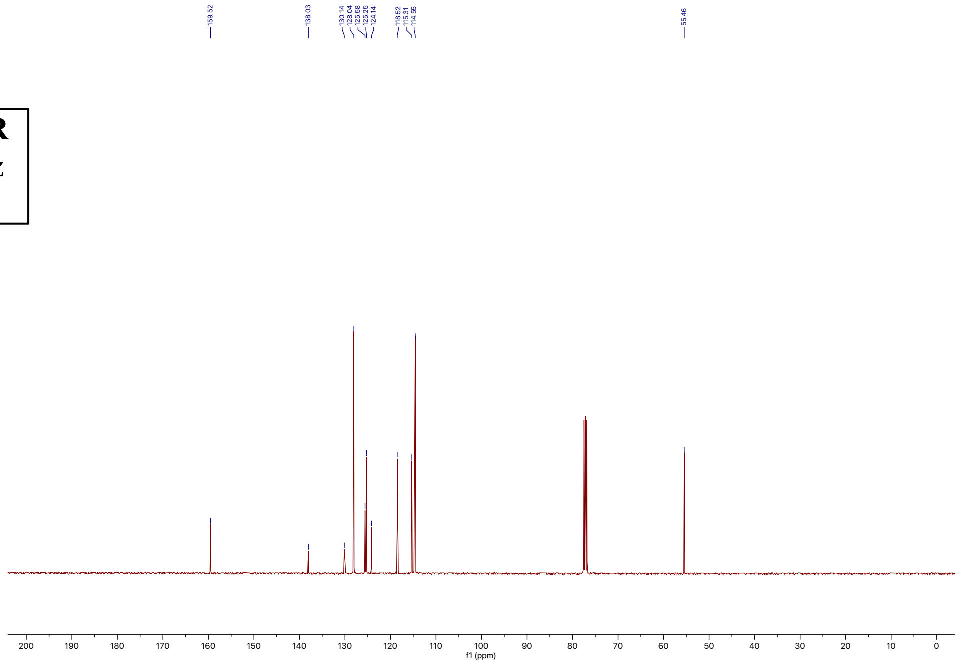

3-(4-(Trifluoromethyl)phenyl)-[1,2,3]triazolo[1,5-a]pyridine (7)

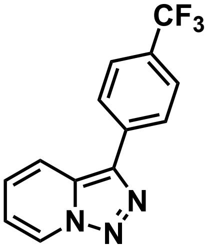

^{1}H-NMR
400 MHz
$CDCl_3$

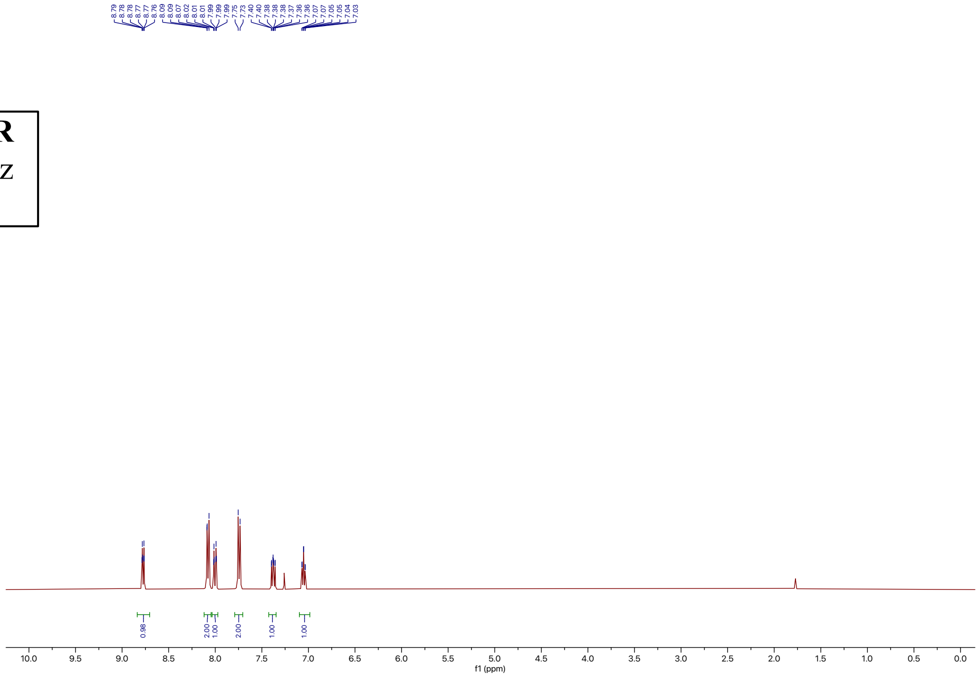

^{13}C-NMR
101 MHz
$CDCl_3$

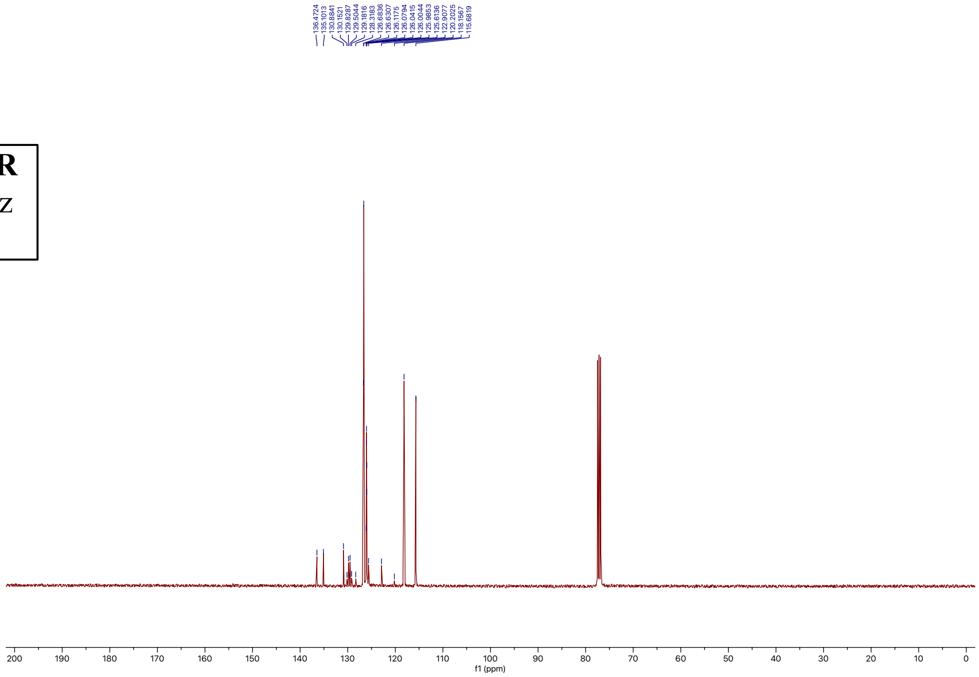

3-(m-Tolyl)-[1,2,3]triazolo[1,5-a]pyridine (8)

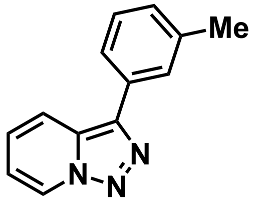

^{1}H-NMR
400 MHz
$CDCl_3$

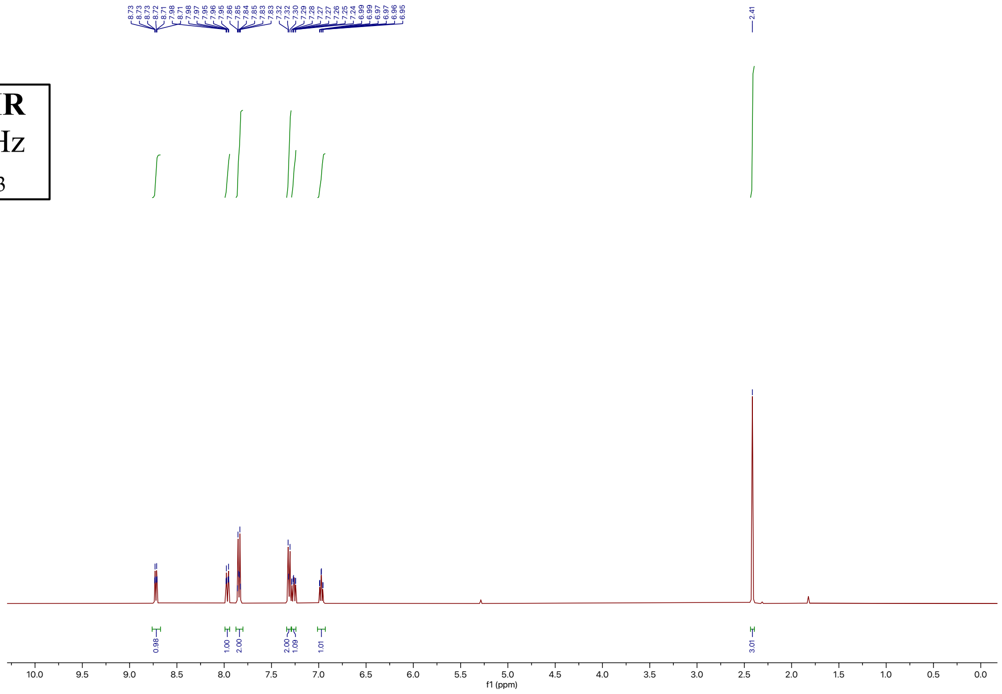

^{13}C-NMR
101 MHz
$CDCl_3$

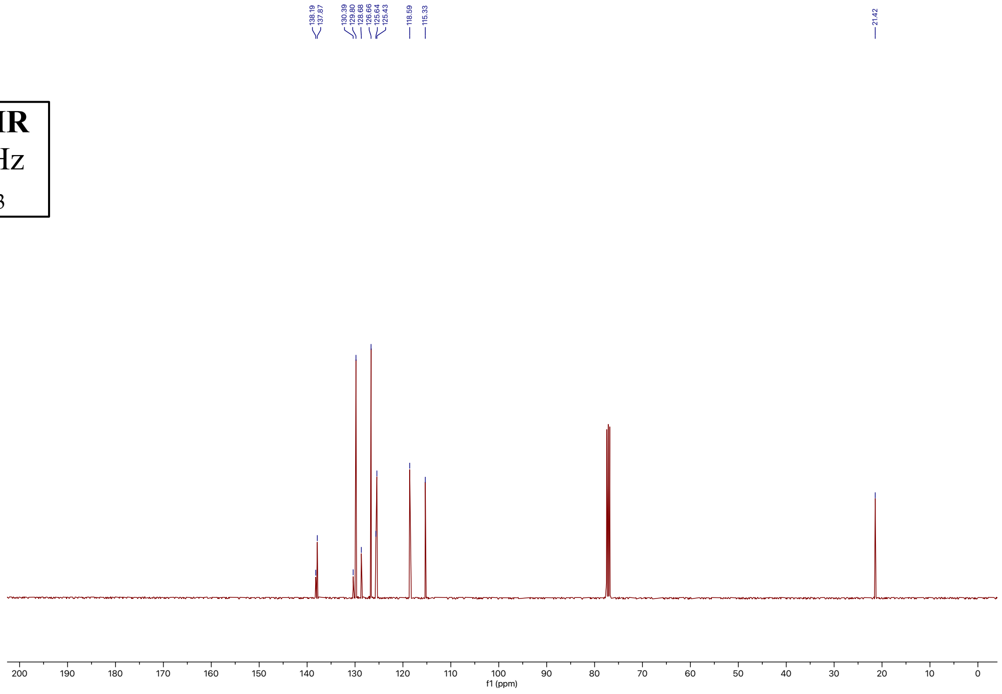

3-(3-Methoxyphenyl)-[1,2,3]triazolo[1,5-a]pyridine (9)

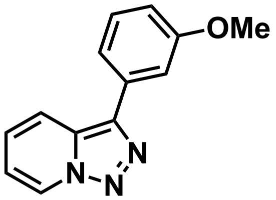

^{1}H-NMR
400 MHz
$CDCl_3$

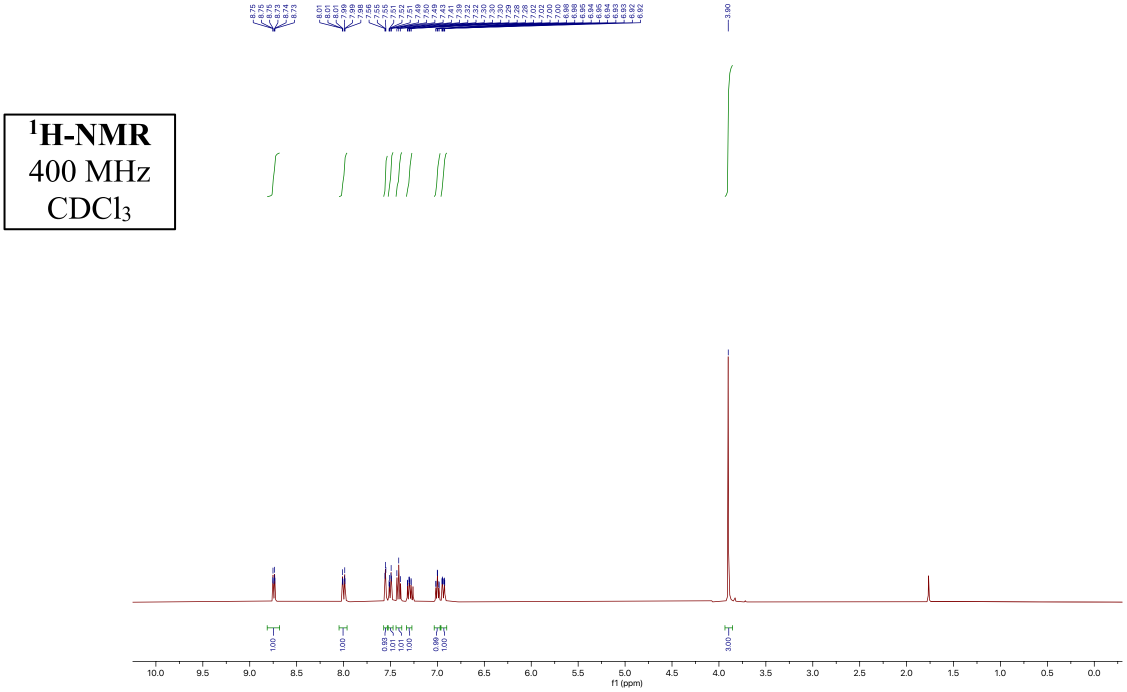

^{13}C-NMR
101 MHz
$CDCl_3$

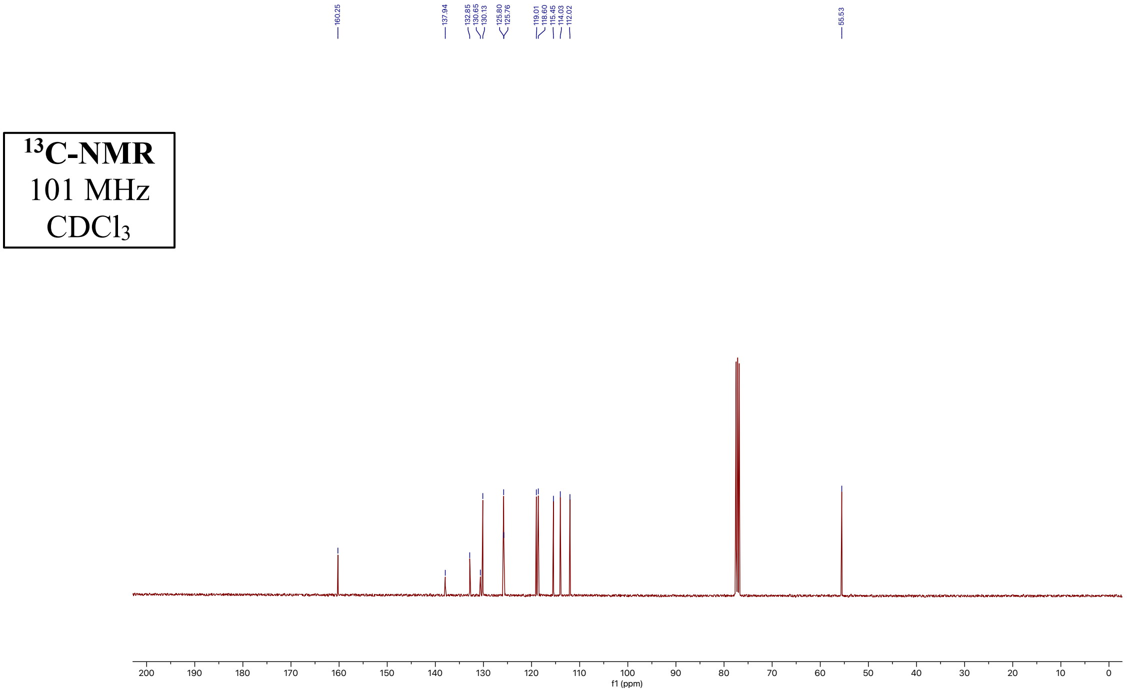

3-(o-Tolyl)-[1,2,3]triazolo[1,5-a]pyridine (10)

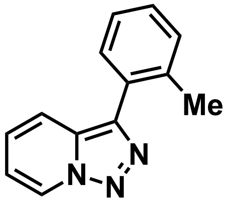

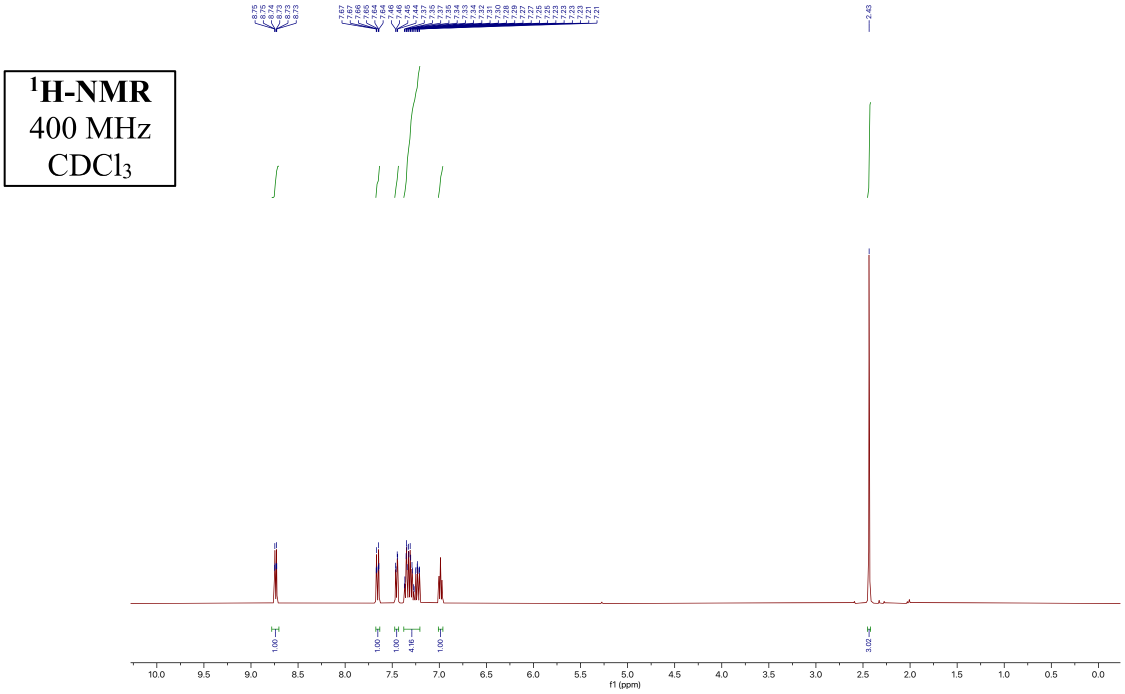

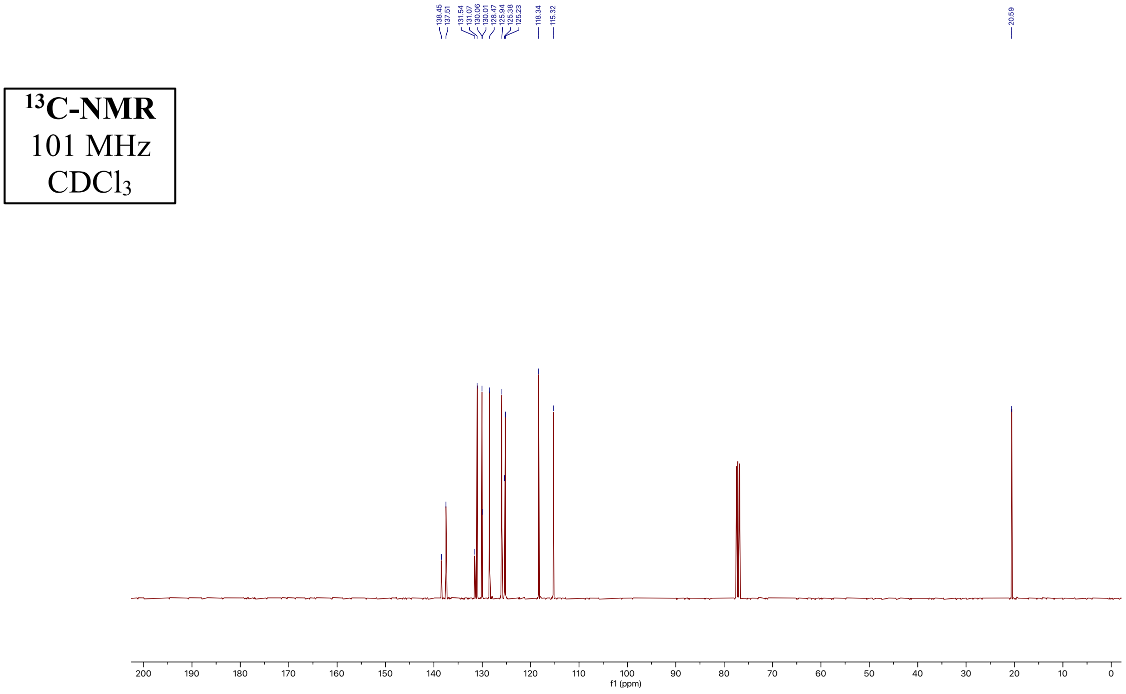

3-(2-Methoxyphenyl)-[1,2,3]triazolo[1,5-a]pyridine (11)

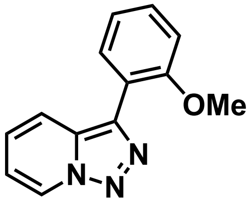

^{1}H-NMR
400 MHz
$CDCl_3$

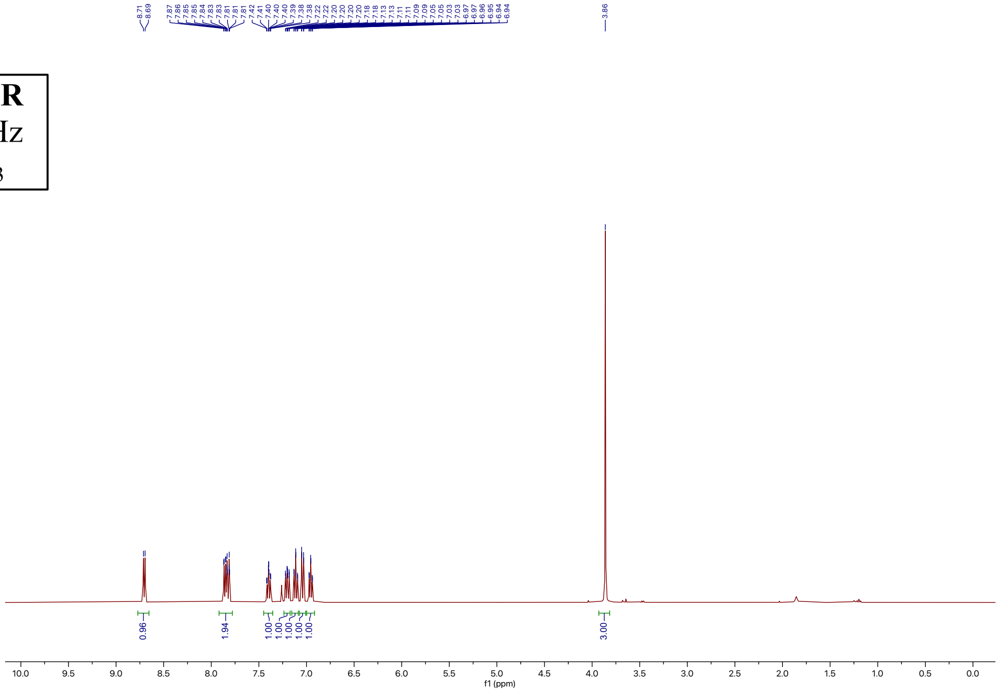

^{13}C-NMR
101 MHz
$CDCl_3$

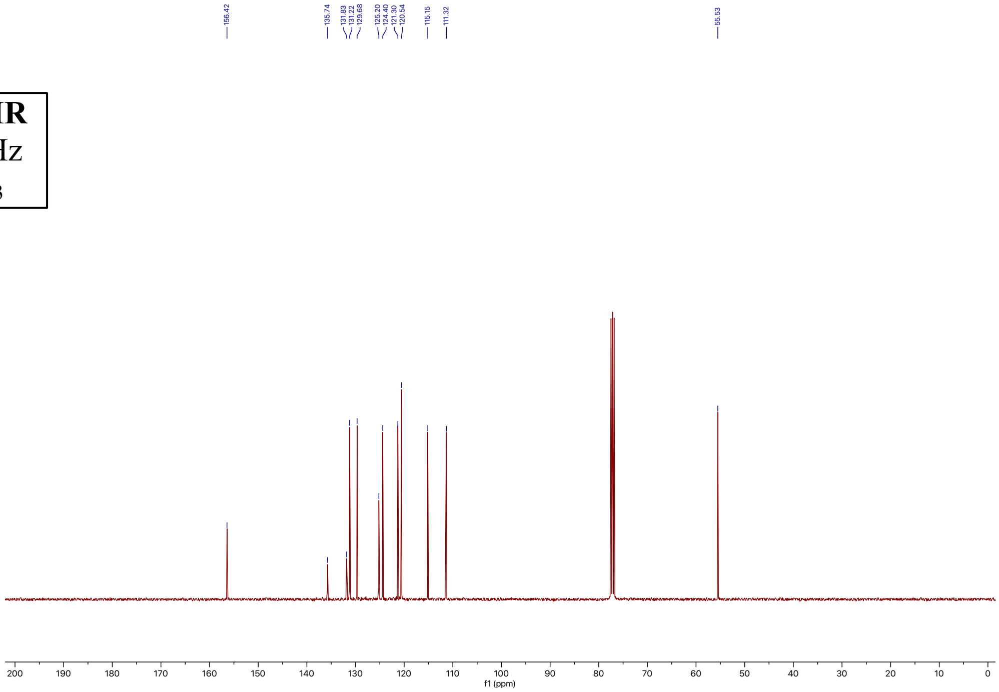

3-(Pyridin-2-yl)-[1,2,3]triazolo[1,5-a]pyridine (12)

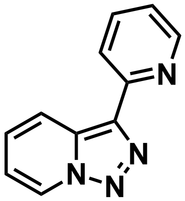

^{1}H-NMR
400 MHz
$CDCl_3$

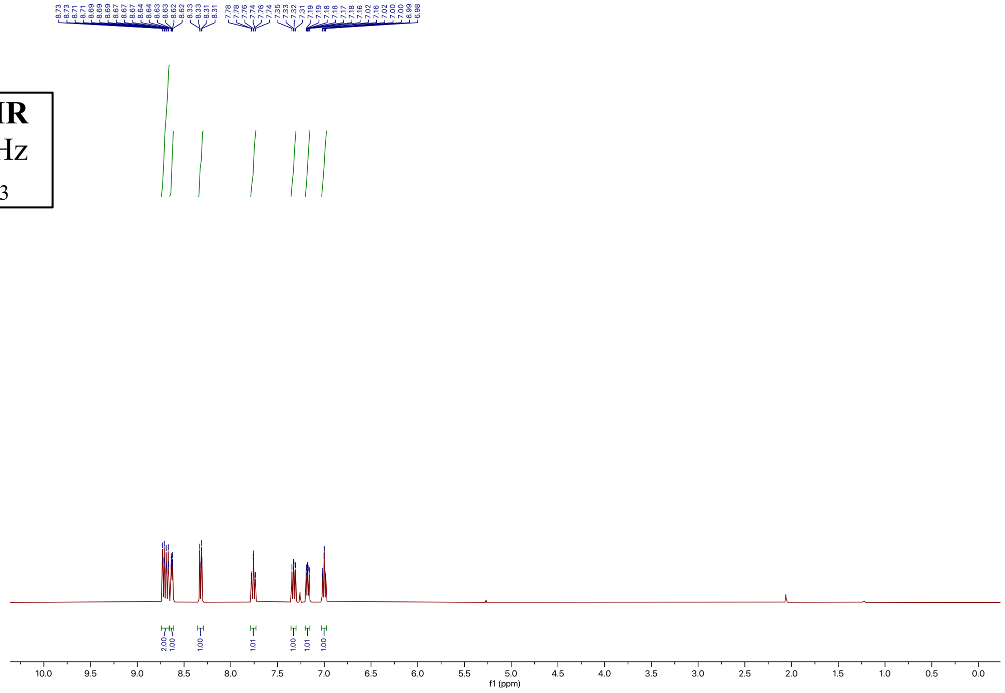

^{13}C-NMR
101 MHz
$CDCl_3$

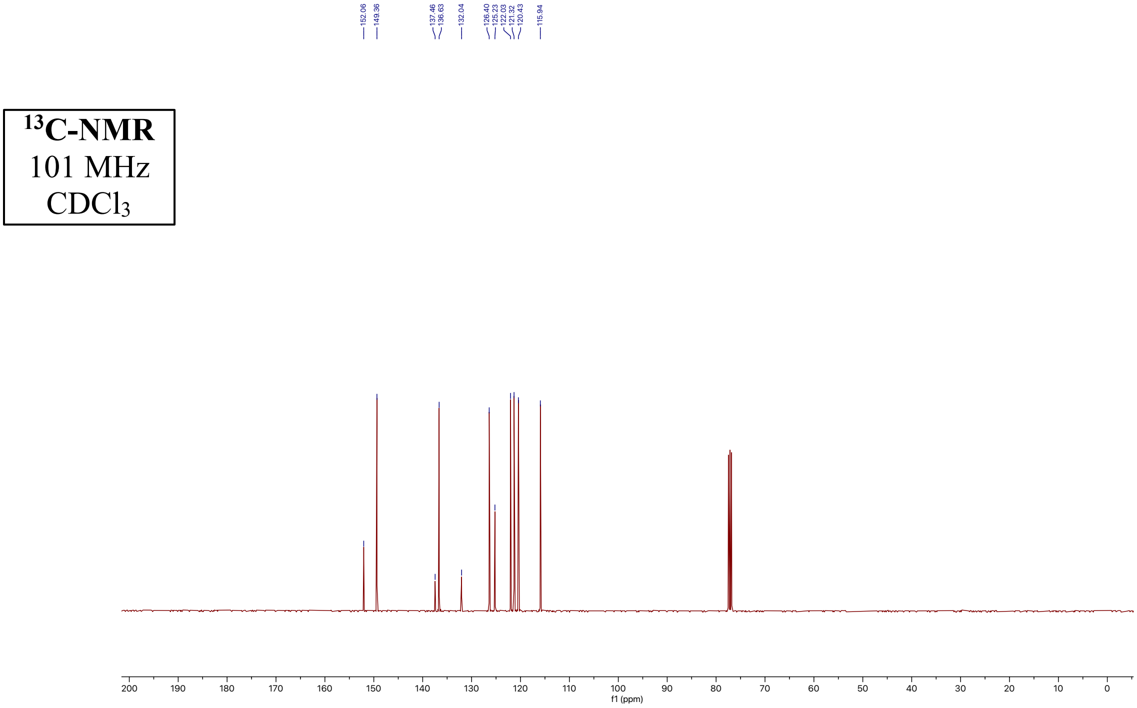

5-Methyl-3-phenyl-[1,2,3]triazolo[1,5-a]pyridine (13)

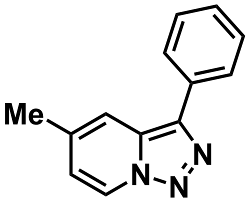

^{1}H-NMR
400 MHz
$CDCl_3$

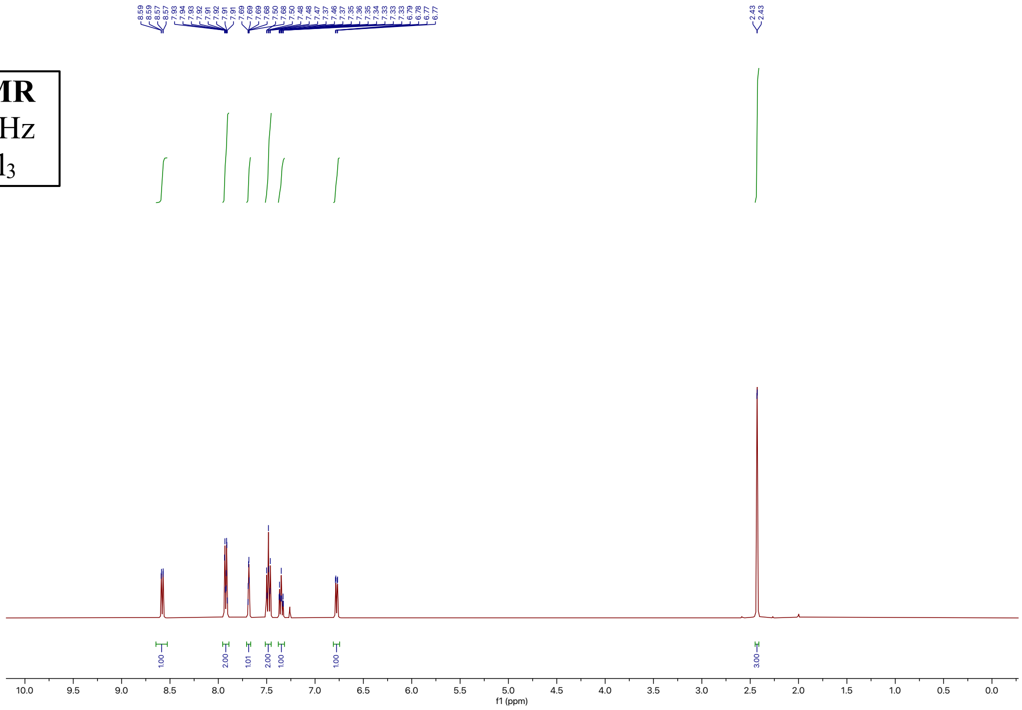

^{13}C-NMR
101 MHz
$CDCl_3$

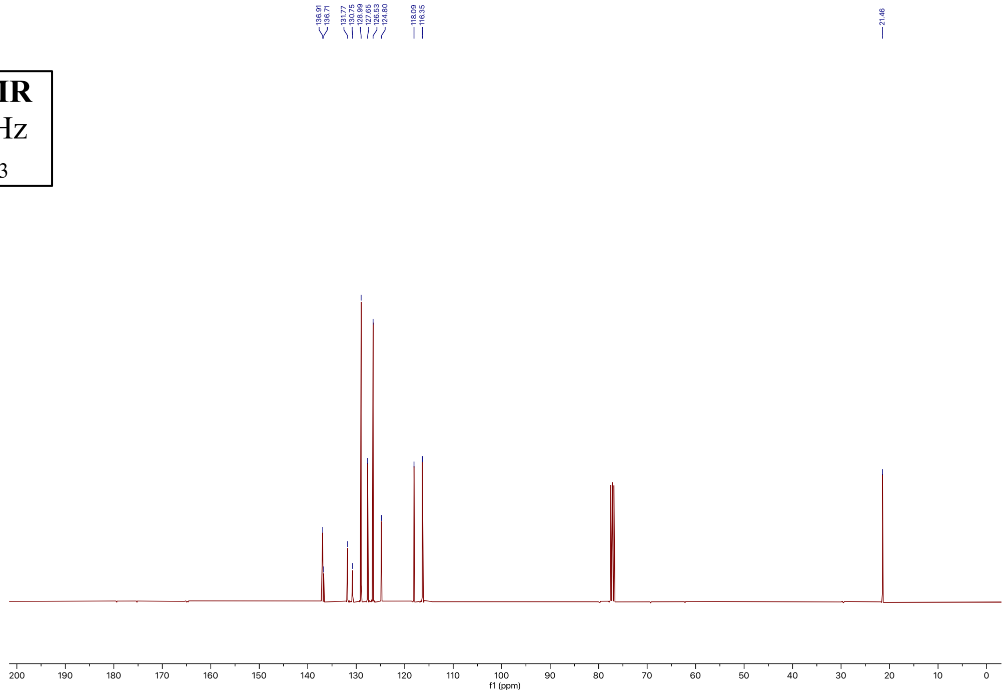

7-Bromo-3-methyl-[1,2,3]triazolo[1,5-a]pyridine (14)

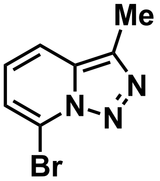

^{1}H-NMR
400 MHz
$CDCl_3$

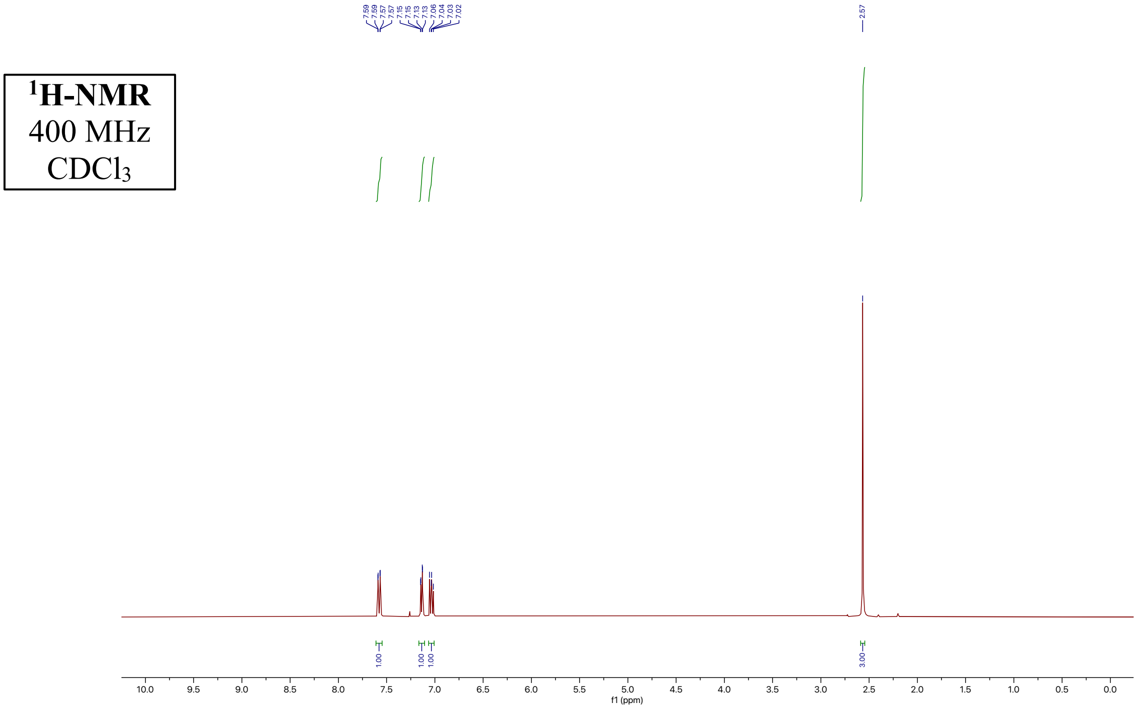

^{13}C-NMR
101 MHz
$CDCl_3$

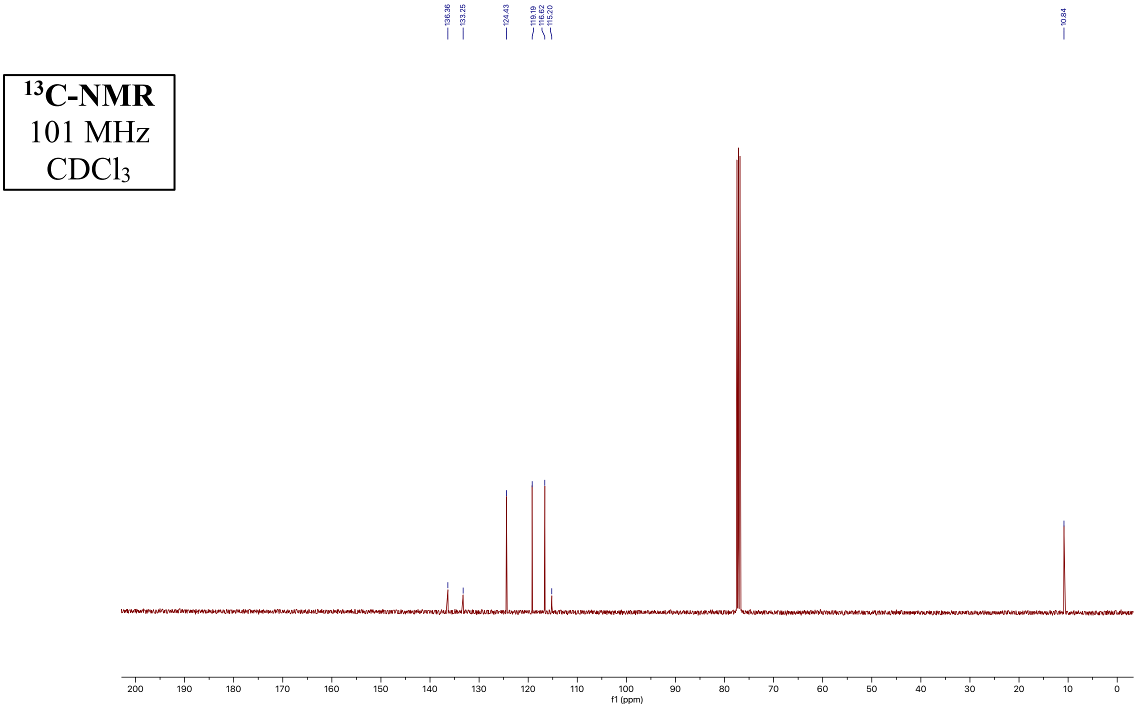

3-Methyl-[1,2,3]triazolo[1,5-a]pyrazine (15)

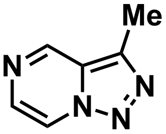

^{1}H-NMR
400 MHz
$CDCl_3$

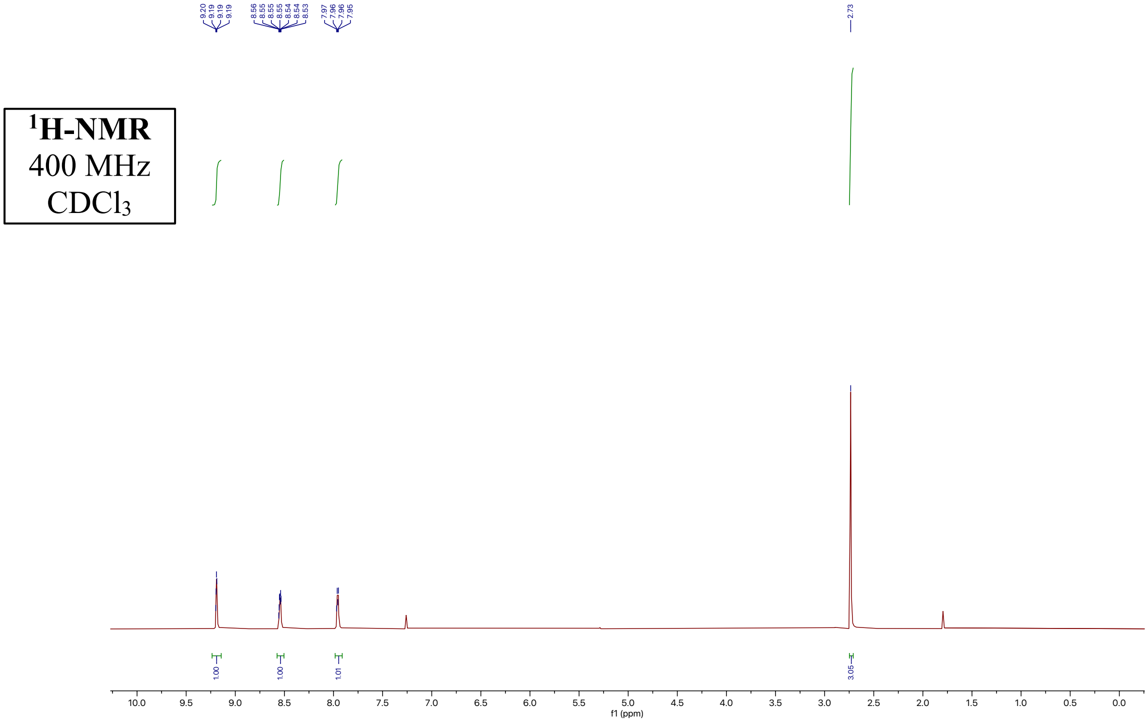

^{13}C-NMR
101 MHz
$CDCl_3$

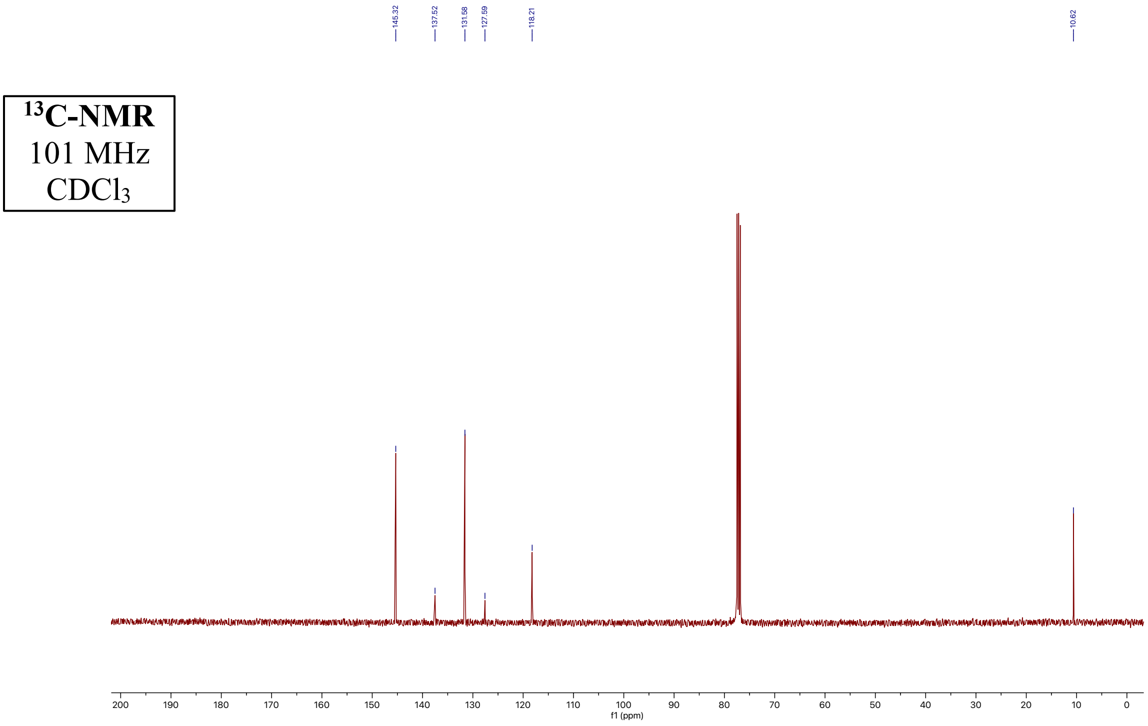

[1,2,3]Triazolo[1,5-a]pyridine (16)

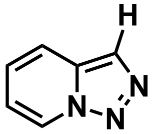

^{1}H-NMR
400 MHz
$CDCl_3$

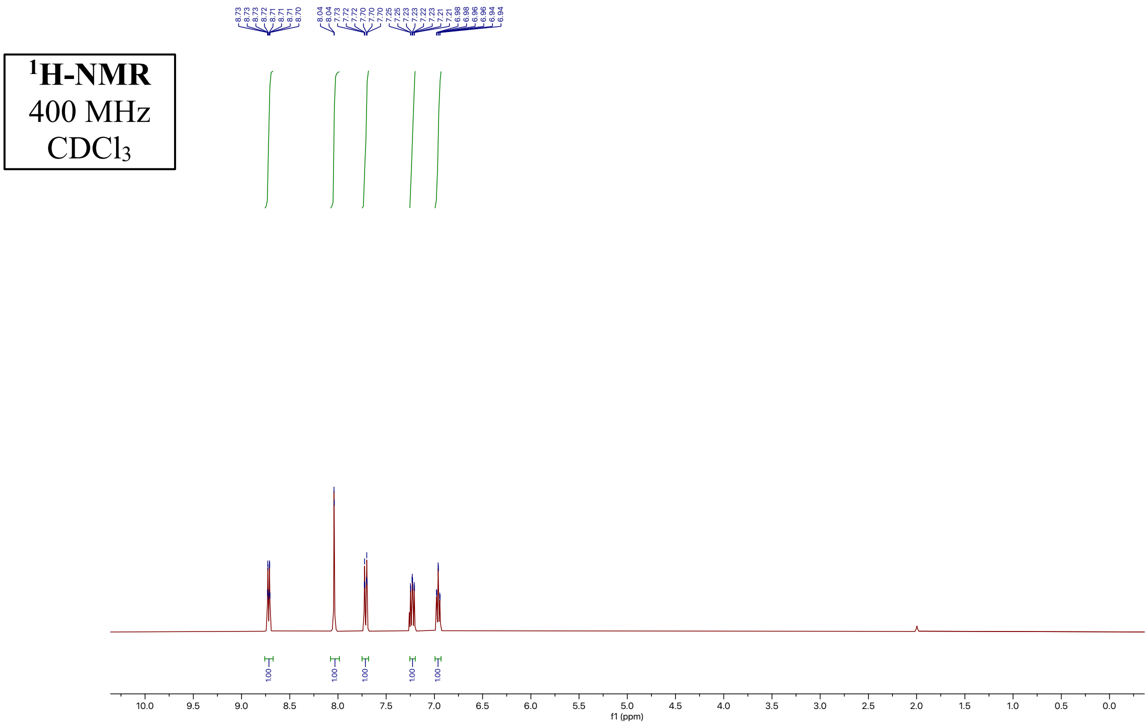

^{13}C-NMR
101 MHz
$CDCl_3$

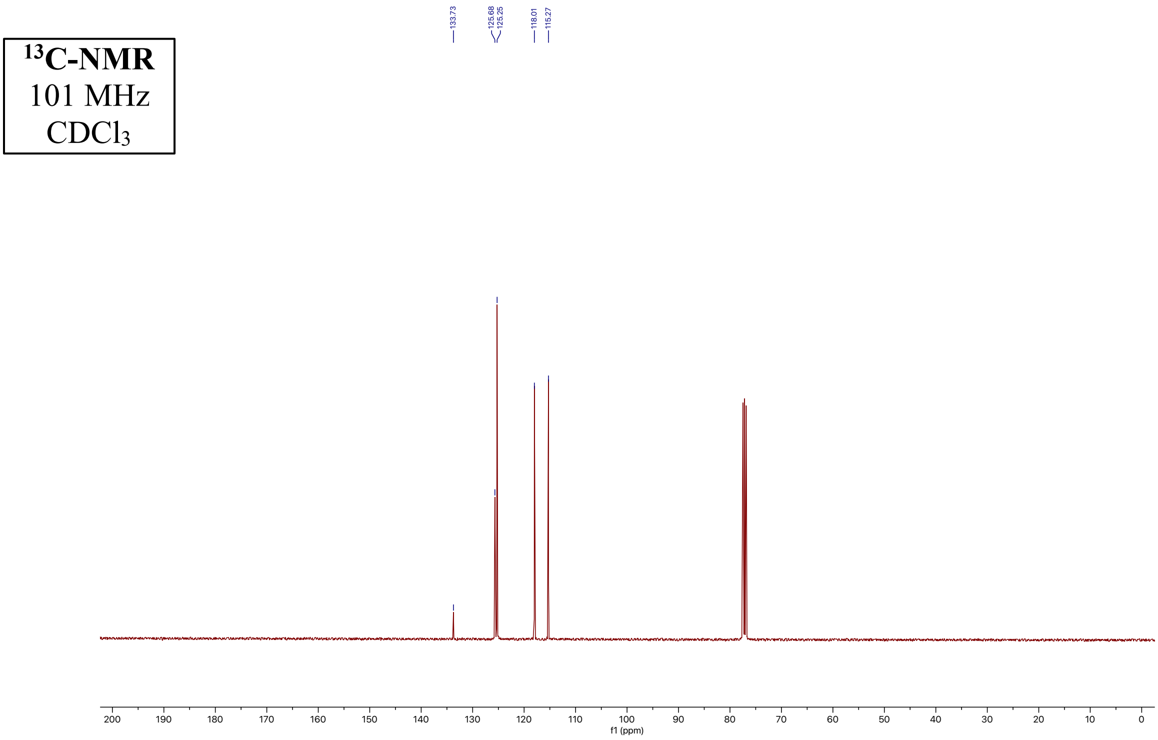

[1,2,3]Triazolo[1,5-b]isoquinoline (17)

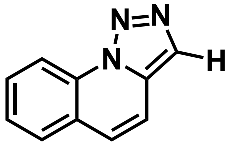

^{1}H-NMR
400 MHz
$CDCl_3$

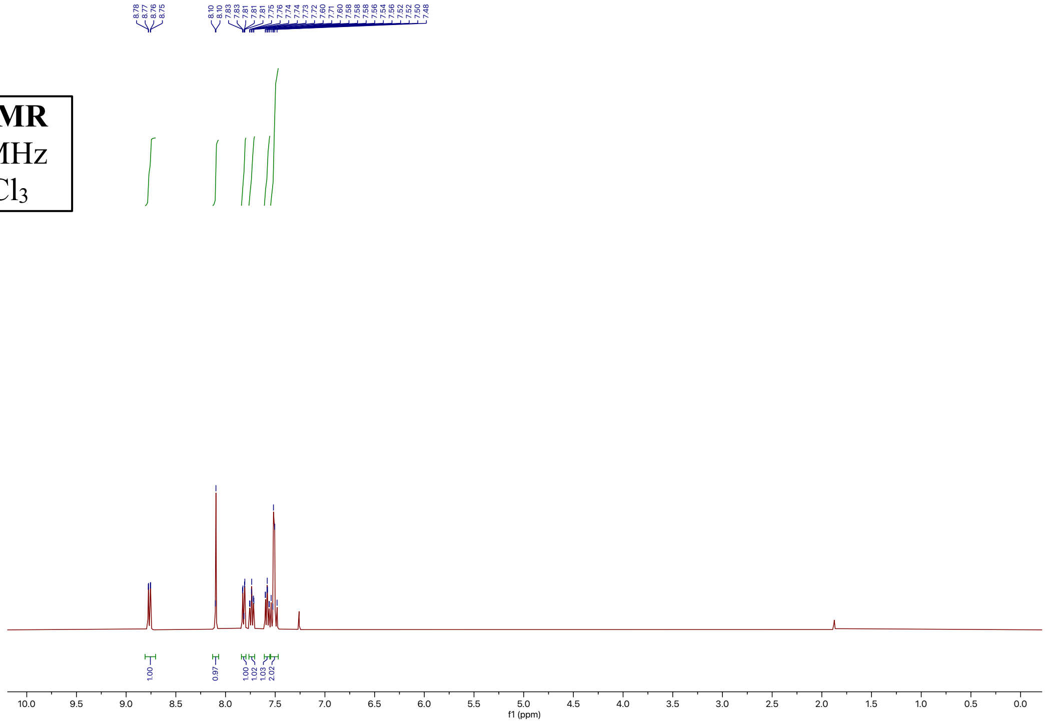

^{13}C-NMR
101 MHz
$CDCl_3$

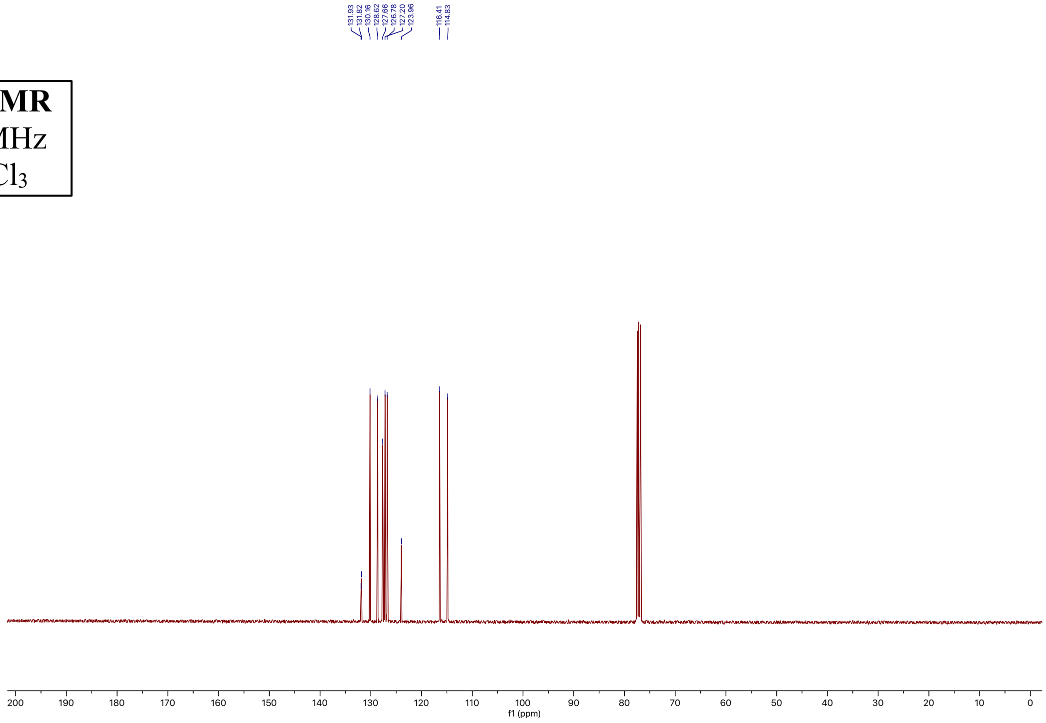

3-Isopropyl-[1,2,3]triazolo[1,5-a]pyridine (18)

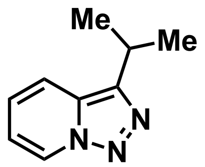

^{1}H-NMR
400 MHz
$CDCl_3$

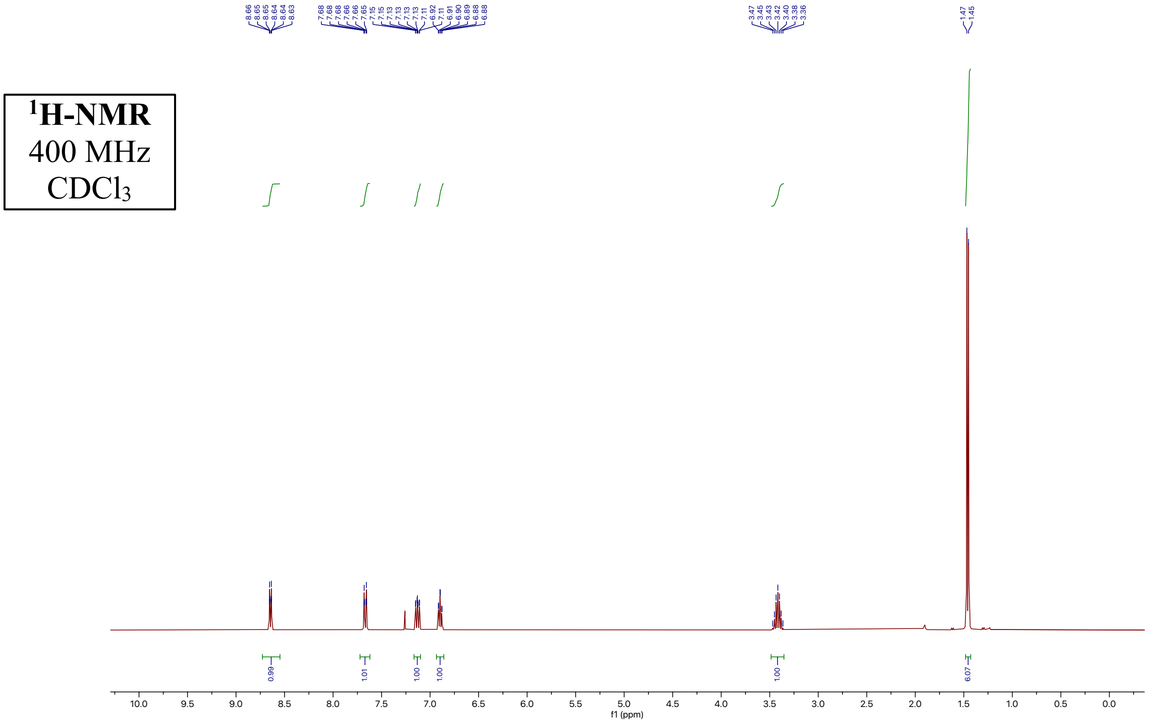

^{13}C-NMR
101 MHz
$CDCl_3$

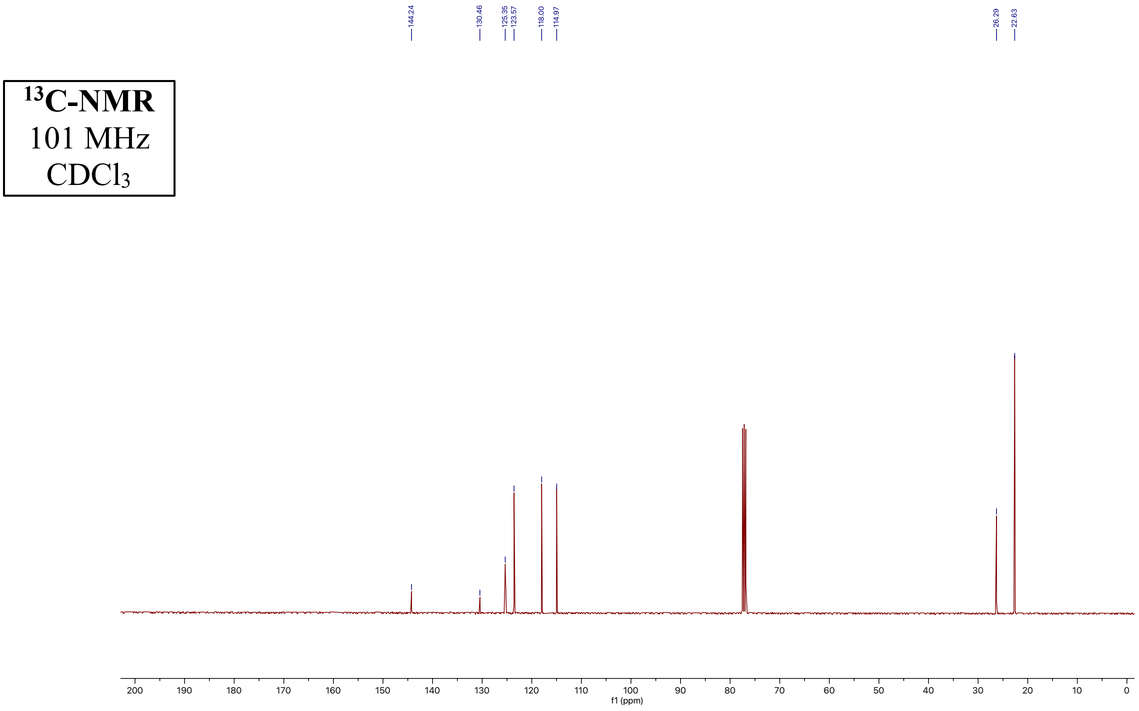

2-Benzhydrylpyridine (20)

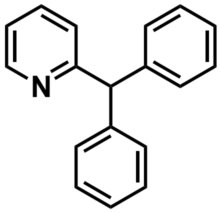

^{1}H-NMR
400 MHz
$CDCl_3$

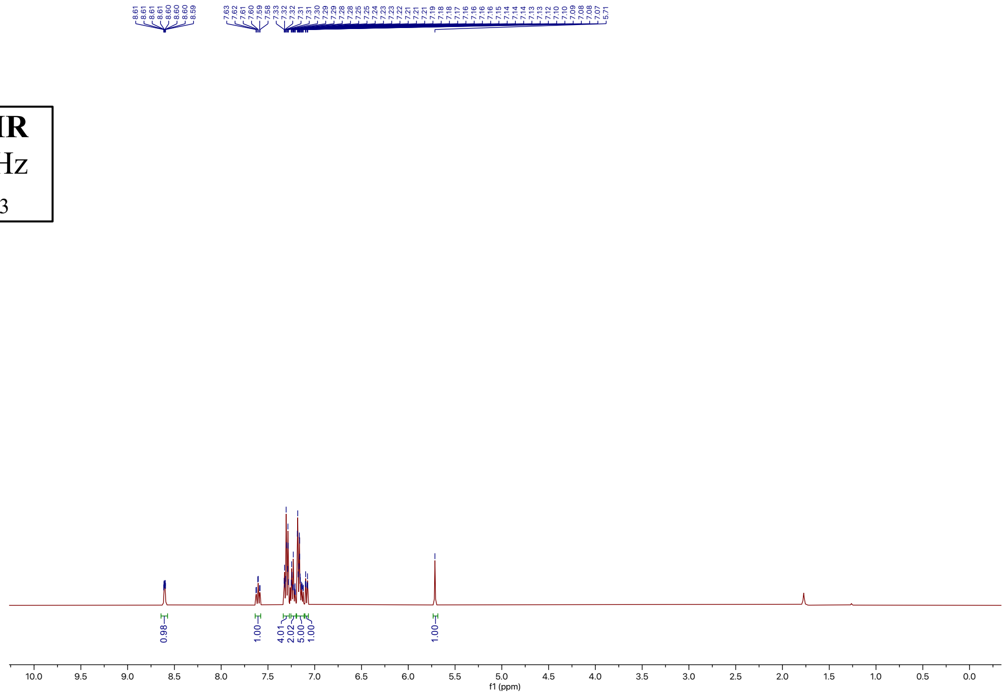

^{13}C-NMR
101 MHz
$CDCl_3$

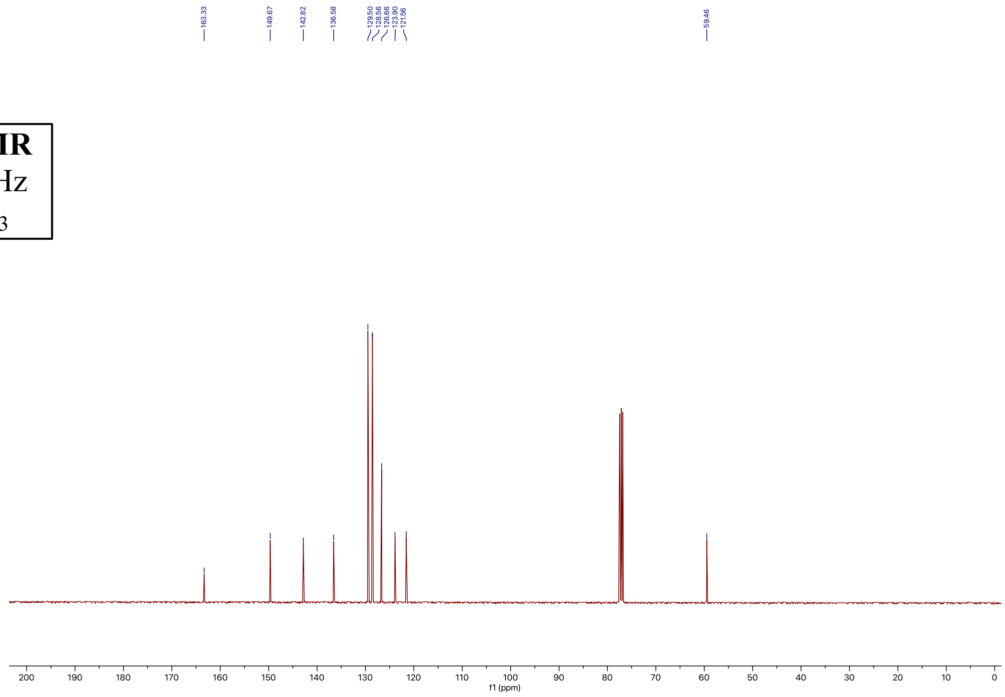

2-(1,2-Diphenylcyclopropyl)pyridine (21)

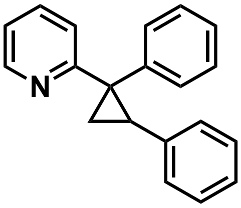

^{1}H-NMR
400 MHz
$CDCl_3$

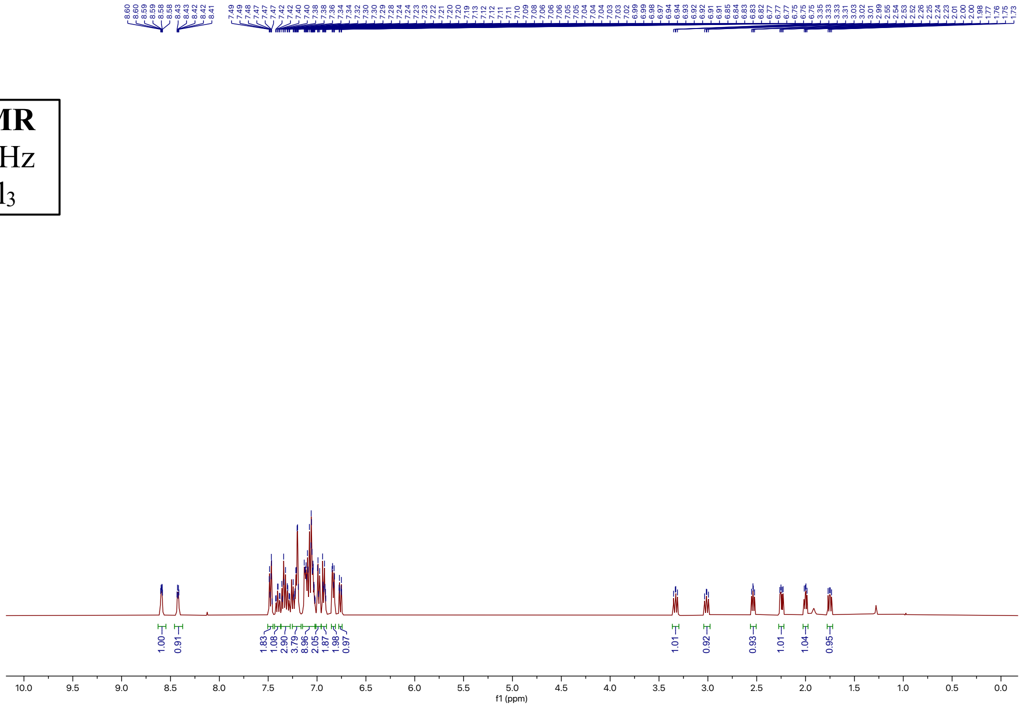

^{13}C-NMR
101 MHz
$CDCl_3$

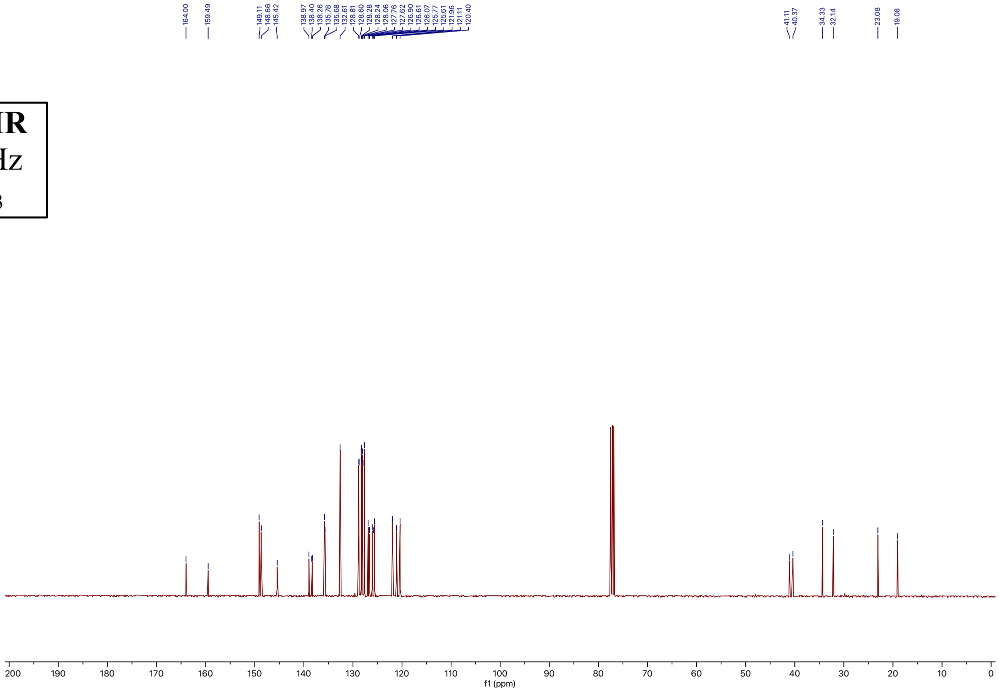

1,3-Diphenylimidazo[1,5-a]pyridine (22)

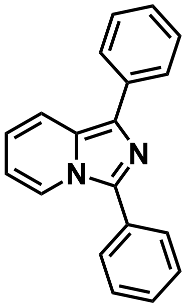

^{1}H-NMR
400 MHz
$CDCl_3$

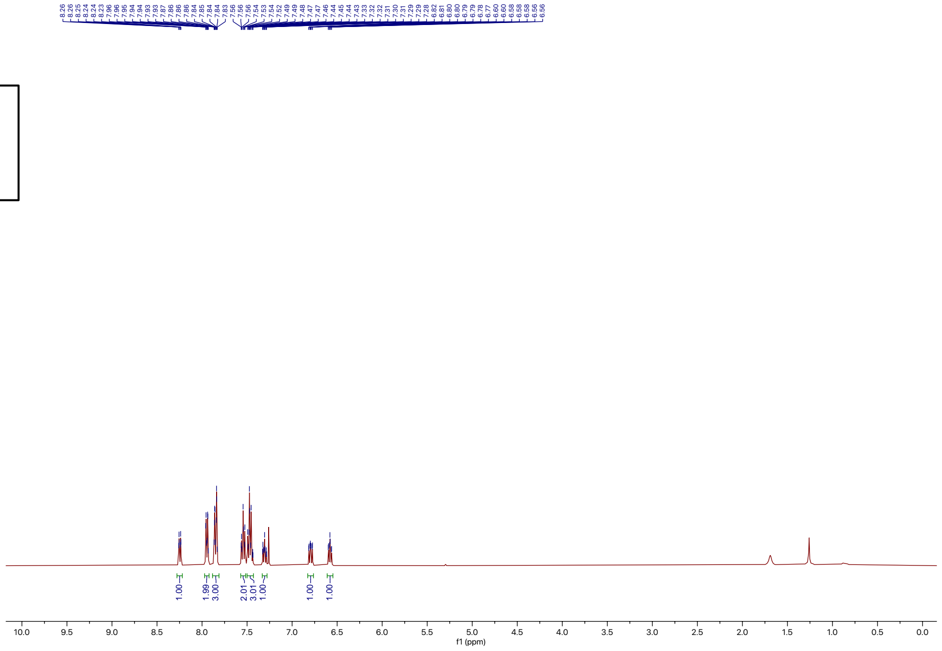

^{13}C-NMR
101 MHz
$CDCl_3$

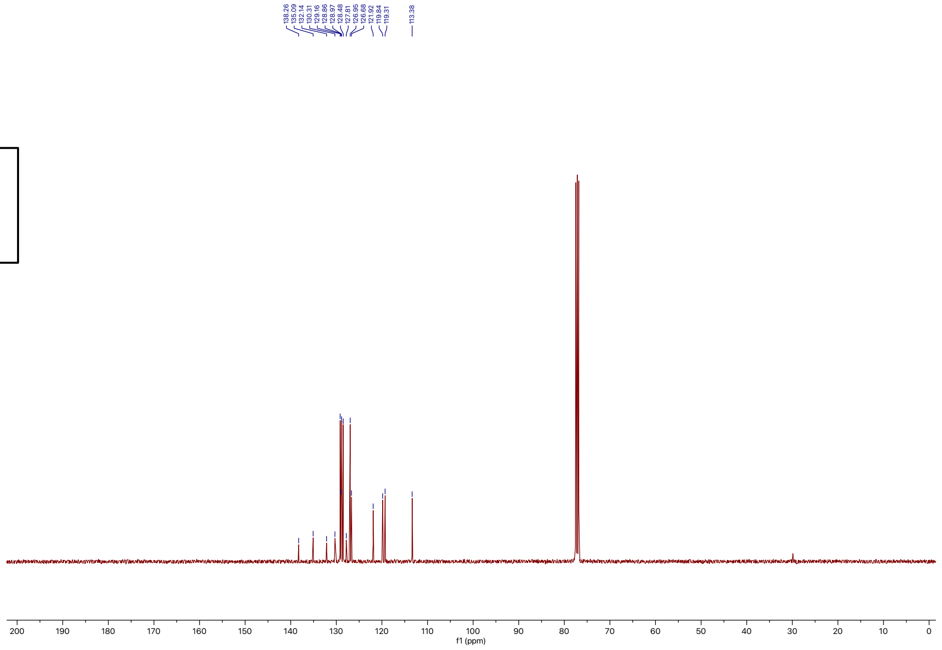

Phenyl(6-phenylpyridin-2-yl)methanone (23)

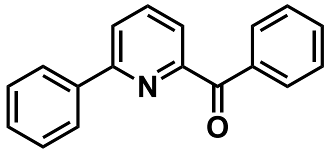

^{1}H-NMR
400 MHz
$CDCl_3$

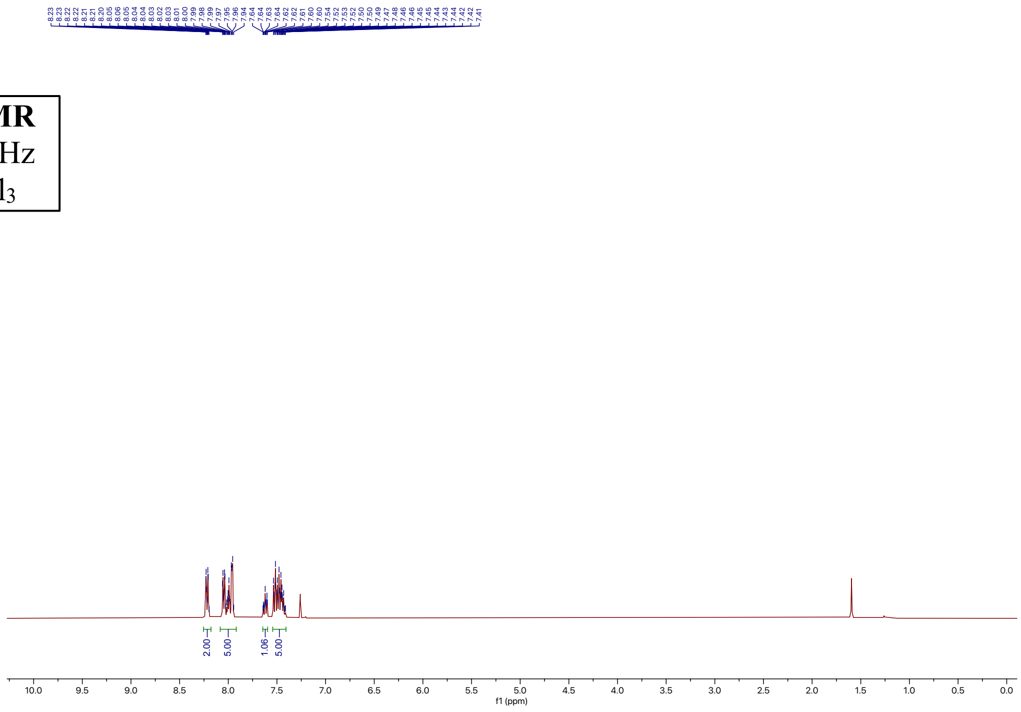

^{13}C-NMR
101 MHz
$CDCl_3$

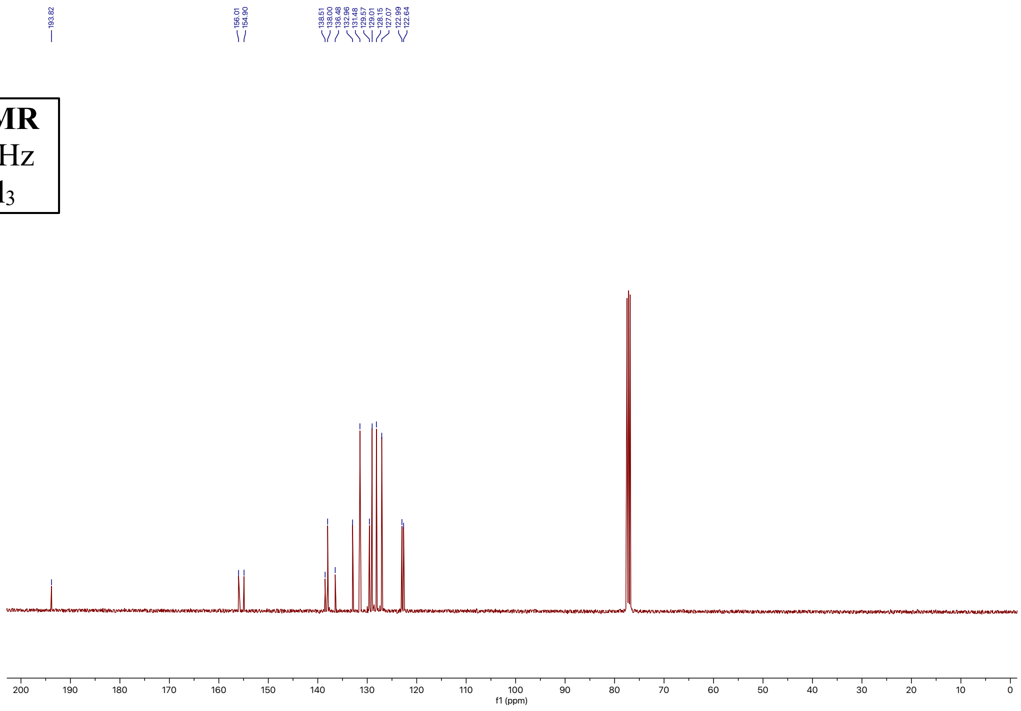

Supplement: Supplementary file 1 [file ol6c00937_si_001.pdf]
